# Probing the prostate tumour microenvironment II: Impact of hypoxia on a cell model of prostate cancer progression

**DOI:** 10.18632/oncotarget.14574

**Published:** 2017-01-10

**Authors:** Claire Tonry, John Armstrong, Stephen Pennington

**Affiliations:** ^1^ Conway Institute of Biomolecular and Biomedical Research, University College Dublin, Belfield, Dublin, Ireland; ^2^ St. Luke's Hospital, Rathgar, Dublin, Ireland

**Keywords:** prostate cancer, tumour microenvironment, biomarkers, proteomics, hypoxia

## Abstract

Approximately one in six men are diagnosed with Prostate Cancer every year in the Western world. Although it can be well managed and non-life threatening in the early stages, over time many patients cease to respond to treatment and develop castrate resistant prostate cancer (CRPC). CRPC represents a clinically challenging and lethal form of prostate cancer. Progression of CRPC is, in part, driven by the ability of cancer cells to alter their metabolic profile during the course of tumourgenesis and metastasis so that they can survive in oxygen and nutrient-poor environments and even withstand treatment. This work was carried out as a continuation of a study aimed towards gaining greater mechanistic understanding of how conditions within the tumour microenvironment impact on both androgen sensitive (LNCaP) and androgen independent (LNCaP-abl and LNCaP-abl-Hof) prostate cancer cell lines. Here we have applied technically robust and reproducible label-free liquid chromatography mass spectrometry analysis for comprehensive proteomic profiling of prostate cancer cell lines under hypoxic conditions. This led to the identification of over 4,000 proteins – one of the largest protein datasets for prostate cancer cell lines established to date. The biological and clinical significance of proteins showing a significant change in expression as result of hypoxic conditions was established. Novel, intuitive workflows were subsequently implemented to enable robust, reproducible and high throughput verification of selected proteins of interest. Overall, these data suggest that this strategy supports identification of protein biomarkers of prostate cancer progression and potential therapeutic targets for CRPC.

## INTRODUCTION

Prostate cancer (PCa) is the second most common cancer in men worldwide. Although the incidence of PCa is high, most men have an indolent form of disease that can be effectively treated with radical prostatectomy, androgen deprivation therapy (ADT), radiotherapy or combinations thereof [1, 2]. ADT plays a central role in the treatment of patients with more advanced PCa, however, over time many patients become resistant to treatment, and develop castration resistant PCa (CRPC) [3]. CRPC generally preludes the onset of metastasis and is therefore the most dangerous and aggressive form of PCa [4]. Previously it was understood that CRPC was ‘androgen independent’ however, recent studies have shown that androgen receptor signaling is actually in some way restored and able to drive PCa progression [5–7].

As with all cancers, the host microenvironment is profoundly altered during tumor growth and this includes becoming hypoxic due to insufficient blood supply. The hypoxic tumour microenvironment correlates with increased tumour aggressiveness, invasiveness and resistance to both radiotherapy and chemotherapy [8]. In PCa, signs of hypoxia and metabolic stress in the prostate tumour tissue are exacerbated following ADT, however, it has been suggested that this hypoxic microenvironment can, in fact, enhance the transcriptional activity of the androgen receptor (AR) [3, 9, 10]. Given the importance of androgen-regulated proteins in PCa development and progression, it is anticipated that further characterization of the role of hypoxia and androgen sensitivity in PCa progression may provide further insight into the mechanisms that drive aggressive, treatment resistant CRPC [11].

As a model platform for investigation of the tumour microenvironment in CRPC the androgen-sensitive LNCaP cell line as well as two androgen-independent sub-lines – LNCaP-abl and LNCaP-abl-Hof - were chosen. Unlike other commonly used cell line models for aggressive PCa (PC3 and DU145), these cell lines express the androgen receptor, which plays a critical role in the evolution from androgen dependent to androgen independent tumour growth in CRPC [12–15]. The LNCaP-abl cell line was initially established after long-term culturing in androgen-depleted medium. Consistent with the CRPC phenotype, LNCaP-abl cells grow much more rapidly than androgen dependent LNCaP cells in hormone-depleted medium and are insensitive to treatment with 5-alpha dihydrotestosterone (DHT) [14, 15]. The LNCaP-abl-Hof cell line was generated from LNCaP-abl cells, which were grown as xenografts in nude mice and subsequently re-cultured [16]. Because of these properties, the LNCaP cell line and its androgen independent sublines have been widely considered as a good *in vitro* model of the *in vivo* tumour conditions in patients who receive ADT and subsequently develop CRPC [12, 17, 18].

Currently, mass spectrometry-based (MS) proteomics technology is regarded as the analytical approach, which can yield the most in-depth information regarding protein expression in experimental samples [19, 20]. Liquid chromatography tandem mass spectrometry (LC-MS/MS) is widely used to identify what proteins are expressed in a given biological sample, and provide a measurement of their abundance. Reproducibility is an essential requirement for MS-based proteomic investigations to ensure that any observations made from the resulting data are truly reflective of pre-defined experimental conditions (drug treatment etc.). The reproducibility and validity of any MS-based proteomics investigation is highly dependent upon the rigour in which the entire workflow – sample preparation, MS analysis, data analysis and biological interpretation of the data – is undertaken [21].

This work described here was undertaken to continue a previous study that was aimed at investigating the impact of glucose deprivation in aggressive PCa (manuscript in press). The primary objective of this study was to utilize mass spectrometry to comprehensively compare the proteome of androgen-independent and androgen-sensitive cell lines under both hypoxic and normoxic conditions. Hypoxic conditions were achieved by treatment of the LNCaP, LNCaP-abl and LNCaP-abl Hof cell lines with dimethyloxalylglycine (DMOG). Rigorous workflows were implemented for identification and verification of protein expression changes attributable to the hypoxic status and/or androgen sensitivity of the cell lines. Each stage of the investigative process was carefully planned to ensure that (i) observed changes in protein expression were not influenced by any experimental or technical bias (ii) potential biological and/or clinical significance was established for any identified proteins of interest and (iii) verification of selected protein of interest could be performed in a robust, reproducible and high throughput manner.

LC-MS/MS analysis led to the identification of a number of candidate proteins that were assembled into panels of putative protein biomarkers of androgen sensitivity and hypoxia for further verification. In addition, these data highlight a number of therapeutic targets, which could be of potential clinical significance for CRPC. Although a cell line model was used, many identified proteins of interest were validated externally using data acquired from tumour tissue and blood samples from patients with PCa. As such, this data provide strong evidence to suggest that the robust, unbiased experimental strategiy employed here can support identification of protein biomarkers of PCa progression and potential therapeutic targets for CRPC.

## RESULTS

### Inducing hypoxia in PCa cell lines

A prolyl hydroxylase inhibitor - dimethyloxalylglycine (DMOG) – was used to induce hypoxia like conditions in the PCa cell lines. Prolyl hydroxylases are central to oxygen-sensing pathways and previous studies have shown that DMOG can be effectively used as a means of mimicking hypoxia through activation of the HIF pathway under non-hypoxic conditions (21% O2) [22]. Cells were incubated in 1mM DMOG for 8 hours to allow for investigation of protein changes that may be reflective of an acute response to hypoxic conditions. Cells were also treated for 24 hours as it has preiously been reported that ‘prolonged’ exposure to hypoxia (chronic hypoxia) induces changes in protein and/or gene expression that differ to those elicited by acute hypoxia. Such ‘adaptive changes’ could play a role in the progression of androgen independent disease [3]. Hypoxic-like conditions were confirmed in all cell lines by assessment of Hif-1 α expression in the LNCaP, LNCaP-abl (Abl) and LNCaP-abl-Hof (Hof) cell lines after 8 hour and 24 hour treatment with DMOG. Western blot analysis revealed increased Hif-1 α expression in cell lines that were treated with DMOG at both timepoints, in contrast to ‘control’ cells (treated with DMSO) (Figure 2A). In all cases, Hif-1 α expression was greater at the 8-hour time point and appeared to be reduced slightly at 24 hours. It was observed that treatment with DMOG had a greater effect on Hif-1 α expression in the androgen-independent cell lines (Abl and Hof) at both time points as opposed to the androgen sensitive (LNCaP) cell line. Indeed, Hif-1 α appeared to be lowly expressed in the LNCaP control cells that had not been treated with DMOG. These observations were common across western blot analysis of all three biological replicates of the three cell lines.

**Figure 1:**
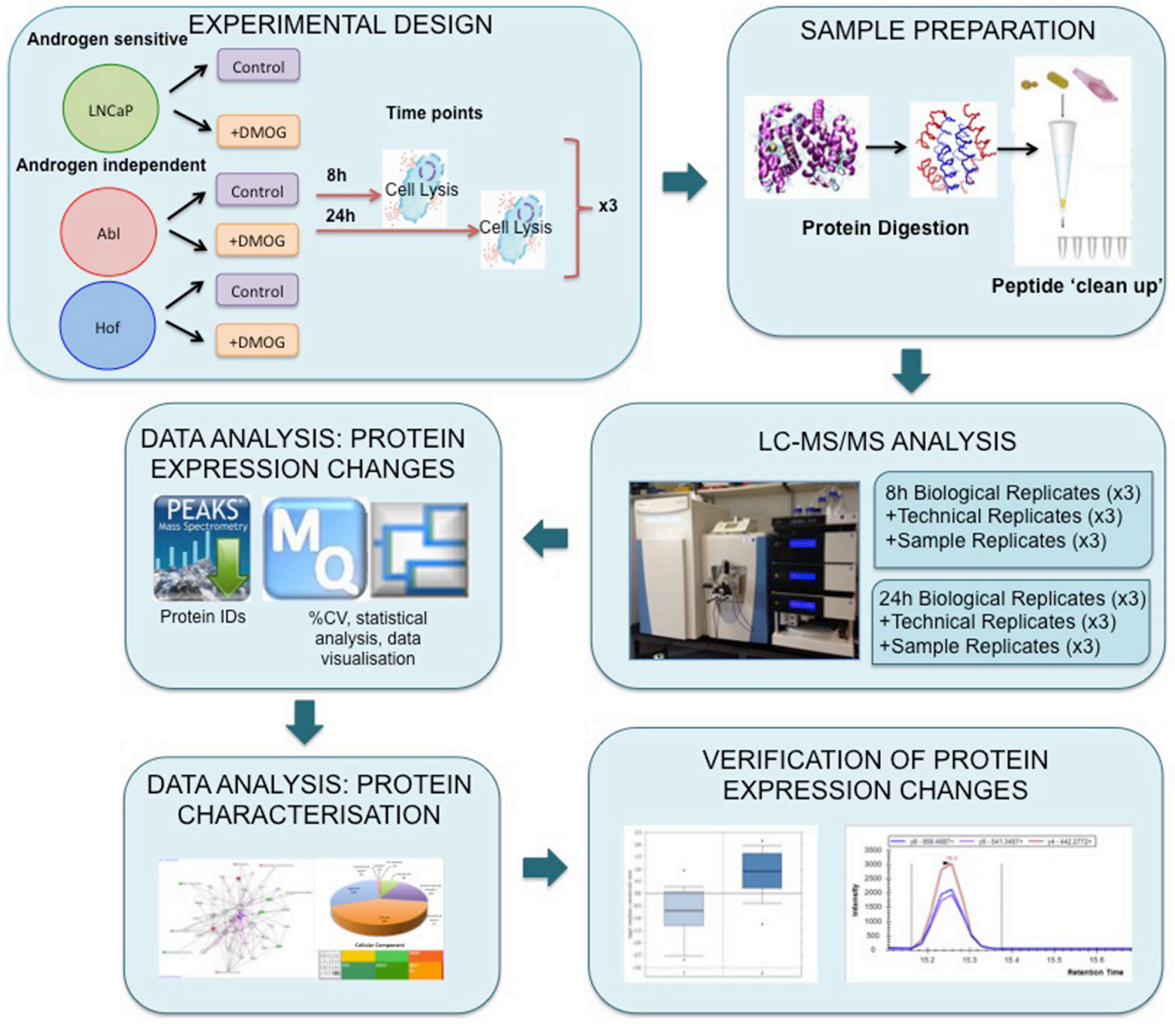
Experimental workflow for proteome scale analysis of the impact of hypoxia in prostate cancer cells Androgen sensitive (LNCaP) and androgen independent (Abl and Hof) cell lines were treated with dimethyloxaloyglycine (DMOG) for 8 h and 24 h. Lysed cells were digested with trypsin and LysC and peptides were purified using C-18 stage tips. Samples prepared from both time points were analysed via LC-MS/MS on a Q-Exactive mass spectrometer with technical replicates (TR) and sample replicates (SR) analysed throughout each run. Data analysis was performed using PEAKS, MaxQuant and Perseus software. Subsequent in silico biological interrogation and validation of protein expression changes was done using PANTHER, IPA and SurvExpress software. MRM assays were designed to further evaluate prioritized proteins of interest.

**Figure 2:**
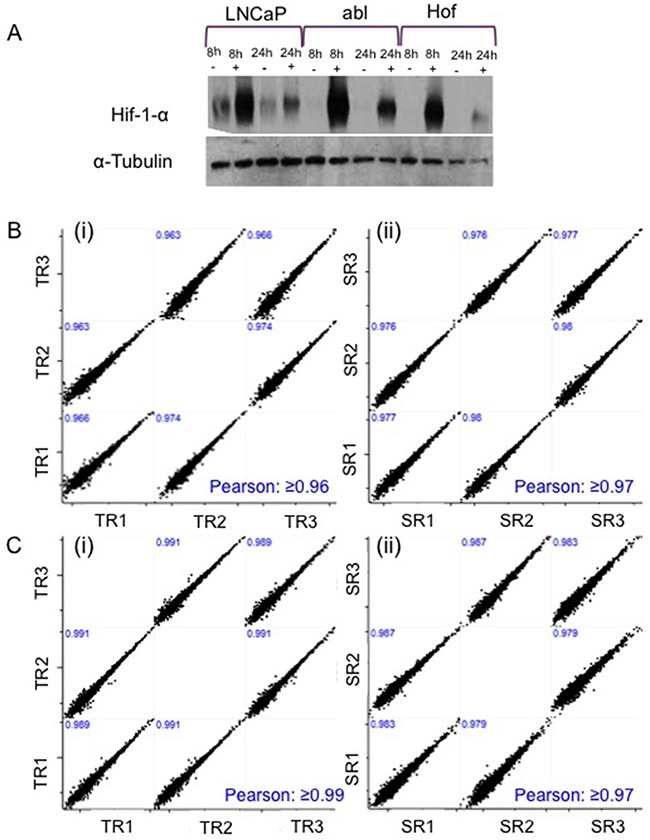
Confirmation of Hypoxic Conditions and Technical Reproducibility Hypoxic conditions following treatment with DMOG were confirmed with expression of Hif-1 alpha in all cell lines after 8 h and 24 h incubation **A**. Sample and Technical reproducibility of the LC-MS/MS analysis was confirmed with scatter plots showing Pearson Correlation values ≥0.9 at both 8 hours **B**. and 24 hours **C**.

### Proteomic analysis of the effect of hypoxic conditions

**Figure 3:**
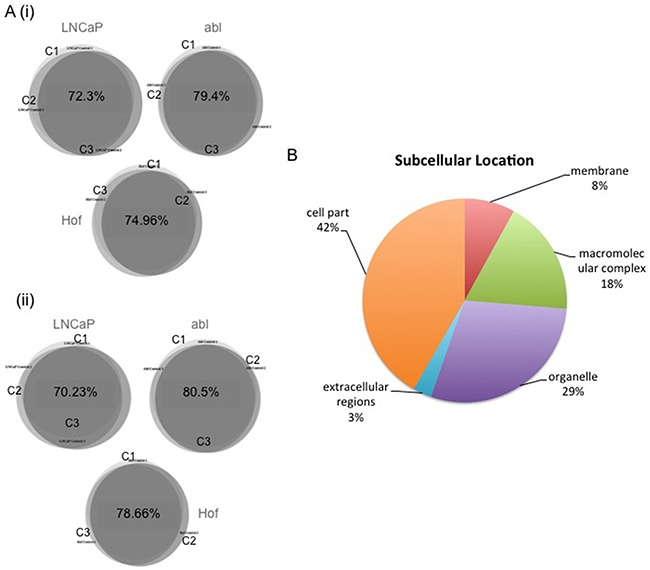
Biological Reproducibility and Subcellular Location of Identified Proteins Biologcal replicates (x3) were generated for all cell lines incubated in DMOG and DMSO (control) for 8 and 24 hours. Biological reproducibility was established with at least 70% overlap in the proteins identified in replicate samples for each control cell line at 8 **A(i)**. hour and 24 hour time points **A(ii)**. Identified proteins spanned a range of subcellular locations **B**.

LC-MS/MS-based analysis was performed on three biological replicates of each cell line – treated and untreated with DMOG for 8 hours and 24 hours - using a Q-Exactive mass spectromter (Figure 1). Analysis of the LC-MS/MS data acquired for the sample replicates (SR) and technical replicates (TR) show high reproducibility with Pearson Correlation values of >0.98 (Figure 2B). Furthermore, there was at least 70% overlap in proteins identified for each biological replicate at both the 8 hour and 24-hour time points – thereby demonstrating excellent biological reproducibility overall (Figure 3A). For both time points over 4,000 proteins were identified across all samples. The subcellular localization of these proteins spanned a variety of cellular compartments including the ‘membrane’ (8%), ‘macromolecular complex’ (18.3%), ‘organelle’ (29.10%) extracellular regions (2.9%) and ‘cell part’ (41.7%) (Figure 3B).

**Figure 4:**
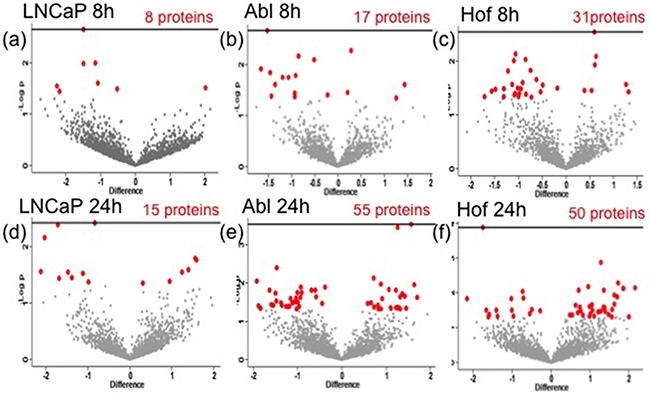
Significantly changing proteins as result of hypoxia in PCa cell lines Student's t-test analysis (p<0.05) was performed on each cell line to determine the effects of DMOG (hypoxia) treatment on protien expression. The volcano plots indicate the statistically significantly changed proteins (red) identified from Student's t-test of each cell line after 8h **a-c**. and 24h **d-f**. treatment with DMOG.

In-depth statistical analysis of the protein expression data was performed using MaxQuant and Perseus software. For each of the individual cell lines, Student's t-tests were performed to assess the number of proteins showing a significant change as result of hypoxic conditions. Figure 4 shows the volcano plots, which reflect this analysis for each cell line. The full list of names and accession numbers of those proteins showing a significant change in each cell line, at each time point, are indicated in Supplementary Data Table 1. In agreement with results observed in the Western blot analysis, hypoxia appears to have had a greater influence on protein expression for androgen-independent Abl and Hof cells, as compared to the androgen sensitive LNCaP cell line i.e. the LNCaP cell line showed the fewest number of significantly changing proteins at both time points. There was no overlap between significantly changing proteins between all cell lines at 8 hours, however, at 24 hours there were 2 proteins (‘U1 small nuclear ribonucleorprotein 70kDa’ and ‘serine/arginine-rich splicing factor 2’) commonly down regulated in LNCaP and Hof cell lines after treatment with DMOG. If anything, some overlap would have been expected between LNCaP and Abl as the Abl cells are directly derived from the parental LNCaP cell line, whereas the Hof cell line was generated after transplant of Abl cells into a mouse model. This lack of overlap, and the unprecedented overlap observed between LNCaP and Hof would indicate that the effects of ‘hypoxia’ may not be cell line specific and may in fact be treatment specific.

**Table 1:** Significant changes in protein expression occurring under hypoxic conditions

Gene	Protein Name	Accession Number	Fold Change	Networks	Location	Type(s)	Biomarker Application(s)	Drug(s)
**LNCaP 8 Hour**								
**STEAP1**	six transmembrane epithelial antigen of the prostate 1	Q9UHE8	+2.023	3	Plasma Membrane	transporter		
**ADI1**	acireductone dioxygenase 1	Q9BV57	-2.265	1	Nucleus	enzyme		
**BRD4**	bromodomain containing 4	O60885	-1.083	6	Nucleus	kinase		
**GSTZ1**	glutathione S-transferase zeta 1	O43708	-1.160	2	Cytoplasm	enzyme		
**MB**	myoglobin	P02144	-1.494	4	Cytoplasm	transporter	diagnosis, safety, unspecified application	
**SLC25A3**	solute carrier family 25 (mitochondrial carrier; phosphate carrier), member 3	Q00325-2	-1.498	5	Cytoplasm	transporter		
**LNCaP 24 Hour**								
**SND1**	staphylococcal nuclease and tudor domain containing 1	Q7KZF4	+1.398	1	Nucleus	enzyme	unspecified application	
**CD59**	CD59 molecule, complement regulatory protein	P13987	+1.554	1	Plasma Membrane	other		
**NOL10**	nucleolar protein 10	Q9BSC4	+1.243		Nucleus	other		
**PUM1**	pumilio RNA-binding family member 1	Q14671-4	+1.589	2	Cytoplasm	other		
**ARPC3**	actin related protein 2/3 complex, subunit 3, 21kDa	O15145	-1.689		Cytoplasm	other		
**EIF3B**	eukaryotic translation initiation factor 3, subunit B	P55884	-2.035	4	Cytoplasm	translation regulator		
**MRPS6**	mitochondrial ribosomal protein S6	P82932	-1.116	3	Cytoplasm	other		
**PSMD14**	proteasome 26S subunit, non-ATPase 14	O00487	-1.380	1	Cytoplasm	peptidase		
**SNRNP70**	small nuclear ribonucleoprotein 70kDa (U1)	P08621-2	-1.717	1	Nucleus	other		
**SRSF2**	serine/arginine-rich splicing factor 2	Q01130	-1.479	1	Nucleus	transcription regulator		
**Abl 8 Hour**								
**RAB5C**	RAB5C, member RAS oncogene family	P51148	+1.251	1	Cytoplasm	enzyme		
**RPL21**	ribosomal protein L21	P46778	+1.437	1	Cytoplasm	other		
**ADRM1**	adhesion regulating molecule 1	Q16186	-1.666	1	Plasma Membrane	other	unspecified application	
**RPS7**	ribosomal protein S7	P62081	-1.524	1	Cytoplasm	other		
**PURA**	purine-rich element binding protein A	Q00577	-1.463	1	Nucleus	transcription regulator		
**TUBA4A**	tubulin, alpha 4a	P68366	-1.439	1	Cytoplasm	other		60+
**CDKN2AIP**	CDKN2A interacting protein	Q9NXV6	-1.358	1	Nucleus	transcription regulator		
**WDR82**	WD repeat domain 82	Q6UXN9	-1.193	1	Nucleus	other		
**HMGN1**	high mobility group nucleosome binding domain 1	P05114	-1.069	1	Nucleus	transcription regulator	unspecified application	
**Abl 24 Hour**								
**AGRN**	agrin	O00468-6	+1.099	1	Plasma Membrane	other		
**ALDH18A1**	aldehyde dehydrogenase 18 family, member A1	P54886-2	+1.449	1	Cytoplasm	kinase		
**ATP5E**	ATP synthase, H+ transporting, mitochondrial F1 complex, epsilon subunit	P56381	+1.059		Cytoplasm	other		
**DRAP1**	DR1-associated protein 1 (negative cofactor 2 alpha)	Q14919	+1.422	2	Nucleus	transcription regulator		
**GUK1**	guanylate kinase 1	Q16774	+1.324	3	Cytoplasm	kinase		
**MARS2**	methionyl-tRNA synthetase 2, mitochondrial	Q96GW9	+1.266	5	Cytoplasm	enzyme		
**NUP85**	nucleoporin 85kDa	Q9BW27	+1.283	9	Cytoplasm	other		
**PDSS2**	prenyl (decaprenyl) diphosphate synthase, subunit 2	Q86YH6	+1.370		Cytoplasm	enzyme		
**RPL13**	ribosomal protein L13	P26373	+1.223	1	Nucleus	other		
**SCO2**	SCO2 cytochrome c oxidase assembly protein	O43819	+1.223	1	Cytoplasm	other		
**SERF2**	small EDRK-rich factor 2	P84101-4	+1.110	1	Other	other		
**SLC25A22**	solute carrier family 25 (mitochondrial carrier: glutamate), member 22	Q9H936	+1.565	8	Cytoplasm	transporter		
**SPINT2**	serine peptidase inhibitor, Kunitz type, 2	O43291	+1.698	1	Extracellular, Space	other	diagnosis	
**UTP6**	UTP6, small subunit (SSU) processome component, homolog (yeast)	Q9NYH9	+1.639	4	Nucleus	other		
**GAPDH**	glyceraldehyde-3-phosphate dehydrogenase	P04406	-1.033	2	Cytoplasm	enzyme	diagnosis, unspecified application	
**ALDOA**	aldolase A, fructose-bisphosphate	P04075	-1.600	1	Cytoplasm	enzyme	unspecified application	
**ATP6V1F**	ATPase, H+ transporting, lysosomal 14kDa, V1 subunit F	Q16864	-1.485		Cytoplasm	enzyme		
**COG3**	component of oligomeric golgi complex 3	Q96JB2	-1.022	7	Cytoplasm	transporter		
**CTBP1**	C-terminal binding protein 1	Q13363-2	-1.921	1	Nucleus	enzyme		
**GTF2I**	general transcription factor IIi	P78347-2	-1.214	1	Nucleus	transcription regulator		
**KLC1**	kinesin light chain 1	Q07866-8	-1.631	2	Cytoplasm	other		
**NAP1L1**	nucleosome assembly protein 1-like 1	P55209-2	-1.005	1	Nucleus	other		
**NDRG1**	N-myc downstream regulated 1	Q92597	-1.885	1	Nucleus	kinase		
**NRP1**	neuropilin 1	O14786	-1.121	1	Plasma Membrane	transmembrane receptor	diagnosis, efficacy	
**PAK2**	p21 protein (Cdc42/Rac)-activated kinase 2	Q13177	-1.477	2	Cytoplasm	kinase		
**PLOD1**	procollagen-lysine, 2-oxoglutarate 5-dioxygenase 1	Q02809	-1.834	1	Cytoplasm	enzyme		
**POFUT1**	protein O-fucosyltransferase 1	Q9H488	-1.501		Cytoplasm	enzyme		
**PPFIA2**	protein tyrosine phosphatase, receptor type, f polypeptide (PTPRF), interacting protein (liprin), alpha 2	O75334-5	-1.373		Plasma Membrane	phosphatase		
**PSIP1**	PC4 and SFRS1 interacting protein 1	O75475	-1.359	2	Nucleus	other	disease progression	
**TKFC**	triokinase/FMN cyclase	Q3LXA3	-1.263	6	Cytoplasm	kinase		
**TPP2**	tripeptidyl peptidase II	P29144	-1.157	2	Cytoplasm	peptidase		
**UBA6**	ubiquitin-like modifier activating enzyme 6	A0AVT1	-1.182		Cytoplasm	enzyme		
**Hof 8 Hour**								
**HNRNPR**	heterogeneous nuclear ribonucleoprotein R	O43390	+1.265	1	Nucleus	other		
**PIGT**	phosphatidylinositol glycan anchor biosynthesis, class T	Q969N2-5	+1.317	3	Cytoplasm	enzyme		
**MIF**	macrophage migration inhibitory factor (glycosylation-inhibiting factor)	P14174	-1.092	1	Extracellular Space	cytokine	diagnosis, prognosis, response to therapy	
**COPS6**	COP9 signalosome subunit 6	Q7L5N1	-1.710	1	Nucleus	other		
**GSTK1**	glutathione S-transferase kappa 1	Q9Y2Q3	-1.003		Cytoplasm	enzyme		
**MYDGF**	myeloid-derived growth factor	Q969H8	-1.019		Extracellular Space	cytokine		
**NONO**	non-POU domain containing, octamer-binding	Q15233	-1.303	1	Nucleus	other		
**PRKCSH**	protein kinase C substrate 80K-H	P14314-2	-1.481	2	Cytoplasm	enzyme		
**RPS12**	ribosomal protein S12	P25398	-1.086	1	Cytoplasm	other		
**SCP2**	sterol carrier protein 2	P22307	-1.133	1	Cytoplasm	transporter		
**SNRPF**	small nuclear ribonucleoprotein polypeptide F	P62306	-1.068	1	Nucleus	other		
**STX3**	syntaxin 3	Q13277-2	-1.061	1	Plasma Membrane	transporter		
**TCEB1**	transcription elongation factor B (SIII), polypeptide 1 (15kDa, elongin C)	Q15369	-1.565	1	Nucleus	transcription regulator		
**WDR1**	WD repeat domain 1	O75083	-1.305	1	Extracellular Space	other		
**Hof 24 Hour**								
**ACTR3**	ARP3 actin-related protein 3 homolog (yeast)	P61158	+1.279	2	Plasma Membrane	other	diagnosis	
**DNMT1**	DNA (cytosine-5-)-methyltransferase 1	P26358	+1.399	2	Nucleus	enzyme	diagnosis	7
**MFN2**	mitofusin 2	O95140	+1.480	1	Cytoplasm	enzyme	unspecified application	
**ACAT2**	acetyl-CoA acetyltransferase 2	Q9BWD1	+1.633	4	Cytoplasm	enzyme		
**CBR4**	carbonyl reductase 4	Q8N4T8	+1.704		Cytoplasm	enzyme		
**DCTN1**	dynactin 1	Q14203-3	+1.063	1	Cytoplasm	other		
**ESYT1**	extended synaptotagmin-like protein 1	Q9BSJ8	+1.107	5	Cytoplasm	other		
**KNTC1**	kinetochore associated 1	P50748	+1.218	2	Nucleus	other		
**MRPL46**	mitochondrial ribosomal protein L46	Q9H2W6	+2.009	1	Cytoplasm	other		
**MRPS36**	mitochondrial ribosomal protein S36	P82909	+1.731	2	Cytoplasm	other		
**NDUFA5**	NADH dehydrogenase (ubiquinone) 1 alpha subcomplex, 5	Q16718	+1.365		Cytoplasm	enzyme		
**PCMT1**	protein-L-isoaspartate (D-aspartate) O-methyltransferase	P22061	+1.368	2	Cytoplasm	enzyme		
**PIGS**	phosphatidylinositol glycan anchor biosynthesis, class S	Q96S52-2	+1.053	3	Cytoplasm	enzyme		
**RPLP1**	ribosomal protein, large, P1	P05386	+1.866	2	Cytoplasm	other		
**SCO1**	SCO1 cytochrome c oxidase assembly protein	O75880	+1.356		Cytoplasm	other		
**SUGT1**	SGT1 homolog, MIS12 kinetochore complex assembly cochaperone	Q9Y2Z0-2	+2.169	2	Nucleus	other		
**TBCE**	tubulin folding cofactor E	Q15813	+1.293		Cytoplasm	other		
**PMPCB**	peptidase (mitochondrial processing) beta	O75439	+1.637		Cytoplasm	peptidase		
**ACTN4**	actinin, alpha 4	O43707	-1.374	2	Cytoplasm	transcription regulator		
**EIF3D**	eukaryotic translation initiation factor 3, subunit D	O15371	-1.012		Cytoplasm	other		
**KPNA4**	karyopherin alpha 4 (importin alpha 3)	O00629	-1.590	1	Nucleus	transporter		
**PRKAR2A**	protein kinase, cAMP-dependent, regulatory, type II, alpha	P13861	-1.741	1	Cytoplasm	kinase		
**PSMD13**	proteasome 26S subunit, non-ATPase 13	Q9UNM6	-1.665	2	Cytoplasm	peptidase		
**RALY**	RALY heterogeneous nuclear ribonucleoprotein	Q9UKM9-2	-1.400	1	Nucleus	other		
**RECQL**	RecQ helicase-like	P46063	-1.581	1	Nucleus	enzyme		
**SNRNP70**	small nuclear ribonucleoprotein 70kDa (U1)	P08621-2	-1.603	1	Nucleus	other		
**SRSF2**	serine/arginine-rich splicing factor 2	Q01130	-2.152	1	Nucleus	transcription regulator		
**TBCA**	tubulin folding cofactor A	O75347	-1.009		Cytoplasm	other		

To further elucidate the changes caused by the DMOG-induced hypoxic microenvironment, proteins identified as statistically significant within each cell line following 8 hour and 24 hour treatment with DMOG (Student's t-test analysis) were analysed using Ingenuity Pathway Analysis (IPA) software. For all cell lines, at both time points, it was possible to identify molecules associated with both cancer metastasis and HIF signaling in the respective network maps generated from the IPA analysis. This analysis also highlighted a number of deregulated molecules that have previously been reported as a cancer biomarker (Table 1).

### The loss of androgen sensitivity measured by proteomic analysis

**Figure 5:**
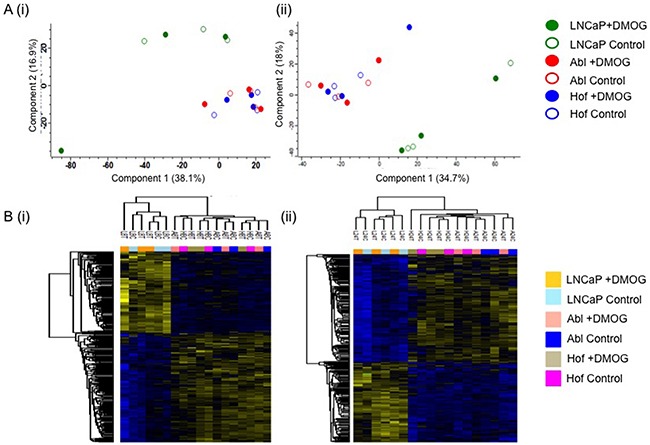
Proteomic Characterisation of Androgen Sensitive and Androgen Independent PCa cell lines The androgen sensitive (LNCaP) and androgen independent (Abl and Hof) cell lines show clear differences in protein epression, irrespective of hypoxic conditions. Principal Component Analysis revealed clear separation between Androgen Sensitive and Androgen Independent PCa cell lines at both 8 hour **A(i)**. and 24 hour **A(ii)**. time points. ANOVA (p≤0.05) revealed 321 and 531 significantly changing proteins at 8 hours **B(i)**. and 24 hours **B(ii)**. respectively.

The cell lines used for this study – LNCaP, Abl and Hof – represent a model of PCa progression from a less aggressive androgen sensitive phenotype (LNCaP) to a more aggressive androgen independent (CRPC) phenotype (Abl and Hof). The androgen independent cell lines are considered to be ‘insesnitive’ to androgen signaling as they are able to proliferate in hormone depleted media and their rate of proliferation is not increased by treatment with physiological levels of androgen 5α dihydrotestosterone (DHT) [14]. Therefore, unbiased analysis of these cell lines following incubation in both hypoxic and normoxic conditions by LC-MS/MS also allowed us to make observations on the molecular impact of androgen sensitivity. Principal Component Analysis (PCA) of all samples separated the androgen-sensitive LNCaP cell line from the androgen-independent Abl and Hof cell lines at both time points (Figure 5A). At the 8 hour time-point 321 proteins showed a significant change in expression across all samples (ANOVA p≤0.05), while expression of 531 proteins was found to be significantly changed after 24 hours incubation with DMOG, across all samples (ANOVA p≤0.05) (Figure 5B). Of these, 216 significantly changing proteins were commonly identified at both the 8-hour and 24 hour time points. As observed in our previous study, which investigated the effects of low glucose conditions in PCa cell lines, a number of proteins that play a role in the adaptive metabolic response of cancer cells to the tumour microenvironment were among those with a significant change in expression between androgen sensitive (LNCaP) and the androgen independent (Abl and Hof) cell lines. In both studies the protein L-lactate dehydrogenase A chain was found to be up regulated in the androgen independent cell lines while contrastingly, the protein L-lactate dehydrogenase B chain was up regulated in the androgen sensitive cell line. Two proteins with known involvement in the TCA cycle (‘Isocitrate dehydrogenase ([NADP], mitochondrial’ and ‘Oxogluterate dehydrogenase’) were also up-regulated in the androgen independent cell lines as opposed to the androgen sensitive cell line. Contrastingly the protein Enolase, which is involved in glycolysis was down-regulated in the androgen independent cell line as opposed to the androgen sensitive cell lines. Again this is in agreement with observations made in our previous study that investgated protein expression changes in LNCaP, Abl and Hof cell lines under low glucose conditions (manusctipt in press). In fact, more than half of the proteins identified from this analysis are in common with those identified as being significantly deregulated between androgen sensitive and androgen independent PCa cell lines in the low glucose study. As such, there is evidence to suggest that the observations made here on protein expression changes can reliably be attributed to loss of androgen sensitivity in PCa cell lines and are likely to be of biological relevance to the development of CRPC.

All proteins identified as significantly changing through ANOVA (p≤0.05) analysis of all samples at both time points were uploaded onto IPA for analysis. The most significant changes between the three cell lines (treated and control) were observed after 24 hours. Comparative analysis of the cell lines at this time point indicated that the AMPK, IL-3 and Androgen Signaling pathways were the most differentially regulated between androgen sensitive (LNCaP) and androgen independent (Abl and Hof) cell lines. The proteins associated with these pathways, which contribute most significantly to the deregulation between androgen sensitive and androgen independent cell lines, are indicated in Table 2. As indicated in this table, a number of these proteins have previously been reported as a cancer biomarker and a number of these molecules are also targetable by various therapeutic agents (Table 2). Aside from those molecules associated with the AMPK, IL-3 and AR-Signaling pathways, there were a number of other molecules identified from both the IPA and Perseus analysis that show a deregulation between androgen sensitive and androgen independent cell lines. A number of these can also be targeted by a number of therapeutic agents and/or combinations of therapeutic agents (Table 3). Almost all of these therapeutic agents are currently FDA approved or under investigation for use in cancer therapy. Notably, of the 12 currently FDA approved therapeutic drugs for PCa, mitoxantrone and docetaxel were found to be therapeutic targets for one of the significantly changed proteins identified in this study – TOP2A (Table 3). In these data TOP2A, which has previously been associated with PCa progression, is significantly up regulated in the androgen sensitive (LNCaP) cell line and down regulated in the androgen independent (Abl and Hof) cell lines at 8 hours. This up or down-regulation was found to be maintained, and even marginally accentuated, after treatment with DMOG (Table 3).

**Table 2:** Deregulated pathways between androgen independent and androgen sensitive PCa cell lines

Cell Line			Abl Control	Abl +DMOG	Hof Control	Hof +DMOG	LNCaP Control	LNCaP +DMOG				
Symbol	Entrez Gene Name	Accession	Exp Log Ratio	Exp Log Ratio	Exp Log Ratio	Exp Log Ratio	Exp Log Ratio	Exp Log Ratio	Location	Type(s)	Biomarker Application(s)	Drug(s)
**AMPK**												
**ACACA**	acetyl-CoA carboxylase alpha	Q13085	-1.086	-0.432	-0.627	-0.801	1.494	1.323	Cytoplasm	enzyme		
**AK4**	adenylate kinase 4	P27144	-0.594	-0.185	-0.701	-0.614	1.375	0.914	Cytoplasm	kinase		
**GNAS**	GNAS complex locus	P63092-3	0.271	1.189	0.44	0.86	-1.438	-0.679	Plasma Membrane	enzyme	unspecified application	
**GYS1**	glycogen synthase 1 (muscle)	P13807-2	-1.222	-0.537	-0.835	0.008	1.077	1.222	Cytoplasm	enzyme		
**HLTF**	helicase-like transcription factor	Q14527	1.144	0.928	0.549	0.3	-0.79	-1.203	Nucleus	transcription regulator	diagnosis	
**PFKL**	phosphofructokinase, liver	P17858	0.061	0.426	1.163	1.181	-0.752	-0.271	Cytoplasm	kinase		
**PFKP**	phosphofructokinase, platelet	Q01813	-1.143	-0.343	-0.912	-0.585	1.246	1.291	Cytoplasm	kinase		
**PPAT**	phosphoribosyl pyrophosphate amidotransferase	Q06203	-0.899	-0.632	-0.68	-0.289	1.272	1.163	Cytoplasm	enzyme		5
**PPM1G**	protein phosphatase, Mg2+/Mn2+ dependent, 1G	O15355	-0.795	-0.844	-0.934	-0.261	0.998	1.203	Nucleus	phosphatase		
**PPP2R2A**	protein phosphatase 2, regulatory subunit B, alpha	P63151	-1.209	-0.509	-0.817	-0.072	1.313	1.391	Cytoplasm	phosphatase		
**IL-3**												
**CHP1**	calcineurin-like EF-hand protein 1	Q99653	0.497	0.945	0.673	0.261	-1.355	-0.798	Cytoplasm	transporter		
**CRKL**	v-crk avian sarcoma virus CT10 oncogene homolog-like	P46109	-1.441	-0.17	-0.3	-0.577	0.656	0.998	Cytoplasm	kinase		
**PPP3CA**	protein phosphatase 3, catalytic subunit, alpha isozyme	Q08209-2	-0.876	-0.791	-0.648	-0.463	1.08	0.977	Cytoplasm	phosphatase		4
**PRKCD**	protein kinase C, delta	Q05655	-0.888	-0.927	-0.89	-0.733	1.14	1.328	Cytoplasm	kinase		1
**PRKD1**	protein kinase D1	Q15139	-0.643	-0.419	-1.239	-0.262	1.088	1.05	Cytoplasm	kinase		
**STAT3**	signal transducer and activator of transcription 3 (acute-phase response factor)	P40763	0.475	0.813	1.102	0.834	-0.722	-1.191	Nucleus	transcription regulator	diagnosis,efficacy,prognosis,response to therapy	
**AR Signalling**												
**CALR**	calreticulin	P27797	0.783	0.6	0.69	1.128	-1.598	-1.297	Cytoplasm	transcription regulator	unspecified application	
**DNAJB1**	DnaJ (Hsp40) homolog, subfamily B, member 1	P25685	-0.872	-0.94	-0.328	-0.775	1.352	1.08	Nucleus	other		
**GNA11**	guanine nucleotide binding protein (G protein), alpha 11 (Gq class)	P29992	0.902	0.922	0.144	0.907	-0.564	-1.485	Plasma Membrane	enzyme		
**GNAS**	GNAS complex locus	P63092-3	0.271	1.189	0.44	0.86	-1.438	-0.679	Plasma Membrane	enzyme	unspecified application	
**GNB2L1**	guanine nucleotide binding protein (G protein), beta polypeptide 2-like 1	P63244	-0.541	-0.008	-1.076	-0.546	1.002	1.255	Cytoplasm	enzyme		
**PRKCD**	protein kinase C, delta	Q05655	-0.888	-0.927	-0.89	-0.733	1.14	1.328	Cytoplasm	kinase		1
**PRKD1**	protein kinase D1	Q15139	-0.643	-0.419	-1.239	-0.262	1.088	1.05	Cytoplasm	kinase		

**Table 3:** Biomarkers and therapeutic targets for Androgen Sensitivity

Average Fold Change Across All Samples														
Drug Target Molecule	Accession Number	LNCaP 8h Control	LNCaP 8h DMOG	LNCaP 24h Control	LNCaP 24 DMOG	Abl 8h Control	Abl 8h DMOG	Abl 24h Control	Abl 24h DMOG	Hof 8h Control	Hof 8h DMOG	Hof 24h Control	Hof 24h DMOG	Number of Drugs
**TOP2A**	P11388	+0.979	+1.548			-0.313	-0.766			-0.360	-0.393			20+
**PARP1**	P09874	+1.050	+1.793	+1.043	+0.884	-0.691	-0.701	-0.878	-0.967	-0.482	-0.498	-0.787	-0.322	8
**CAT**	P04040	-1.102	-1.491	-1.633	-1.200	+0.113	+0.214	+0.541	+0.486	+0.957	+0.919	+0.778	+1.012	1
**TXN**	P10599	+1.007	+0.830	+0.849	+1.133	-0.513	-0.912	-1.138	-0.572	-0.658	-0.773	-0.688	-0.599	1
**ABCC1**	P33527			+1.345	+1.154			-0.754	-0.335			-0.598	-0.318	1

### Selection of proteins of interest for further verification

A large number of proteins, which show a significant change in expression as consequence of androgen sensitivity and/or hypoxic conditions, were identified in this study. As such, the findings reported here can potentially be used for (i) the identification of biomarkers that would provide an indication (at an early stage) of the emergence of androgen independent PCa and (ii) the identification of potential pathways of interest and/or therapeutic targets which may be of value in elucidating the adaptive response of both non-aggressive (androgen sensitive) and aggressive (androgen independent) PCa to hypoxic conditions. In answer to these questions, unbiased analysis of the LC-MS/MS data highlighted a number of significantly differentially expressed proteins (Figure 4-5, Tables 1-3). Instead of arbitrarily selecting a handful of significant proteins to further evaluate by traditional antibody-based techniques, we sought to evaluate as many as possible by multiple reaction monitoring (MRM), a more high-throughput and cost-effective approach that does not rely on the availability of antibodies. All significant proteins were categorized based on their association with either androgen sensitivity (AS) or hypoxia (Hx) and consolidated into panels for further verification. The prioritization of proteins to be included in both panels was based on them having being measured with a coefficient variation (CV) less than 20% in the LC-MS/MS analysis – as determined based on SR and TR measurements. This resulted in a list of 110 proteins in the AS panel and 147 proteins in the Hx panel. Fifty-one of the proteins shortlisted for the AS panel were common to proteins shortlisted for an AS panel in the previously described ‘low glucose’ study, selected under the same criteria (Supplementary Data Table 2). Within the Hx panel, 4 of the 147 proteins have previously been identified and classified as a biomarker for hypoxia; ‘macrophage migration inhibitory factor’, ‘aldolase A, fructose-bisphosphate’, ‘Inositol 1,4,5-trisphosphate receptor type’ and ‘N-myc downsteam regulated 1’.

### Biological interrogation of selected proteins of interest

**Figure 6:**
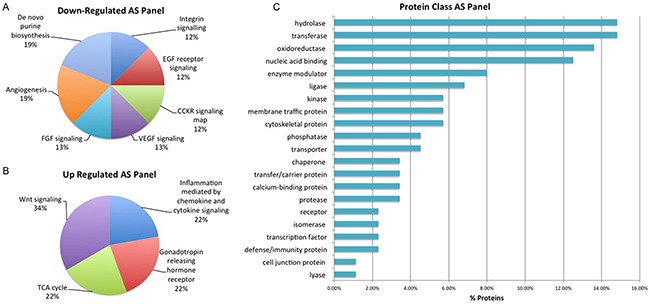
Biological Interrogation of Androgen Sensitivity (AS)-Associated Proteins of Interest Panther analysis was performed on proteins selected based on their association with androgen sensitivity in this study. Pathways associated with cancer progression were represented by both up and down-regulated protiens of interest **A-B**. The majority of proteins were classified as hydrolases, transferases and oxidoreduuctase **C**.

**Figure 7:**
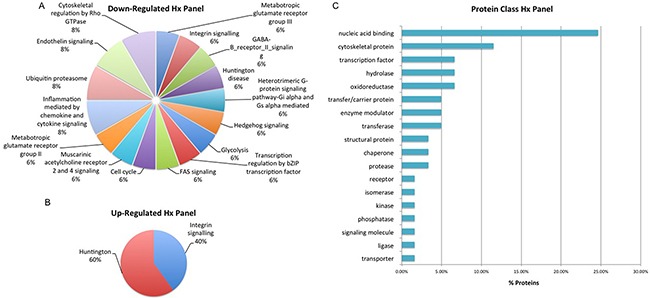
Biological Interrogation of Hypoxia (Hx)-Associated Proteins of Interest Panther analysis was performed on proteins selected based on their association with hypoxia in this study. The majority of down-regulated proteins map to a large number of signaling pathways **A**. while up-regulated protiens were associated to the Integrin Signalling and Huntington pathways **B**. The majority of proteins were classified as nucleic acid binding proteins **C**.

Selected proteins of interest were further characterized using annotation or prediction results from various bioniformatic platforms. PANTHER analysis was used to identify which proteins are associated with the cell membrane (2% AS), organelle (38% AS, 37% Hx), macromolecular complex (17% AS, 15% Hx) and cell part (43% AS, 48% Hx) while SignalP (version 4.3) and Phobius software was used to identify ‘secreted’ proteins. In the AS panel 17 ‘secreted’ proteins were identified while in the Hx panel 15 ‘secreted’ proteins were identified. Some of the ‘secreted’ proteins from the AS and Hx panels – CATH, CD59, CALR and MYO6 – have previously been identified in serum-based PCa biomarker discovery experiments within our own research group and by others [23, 24, 25]. As described previously, IPA analysis highlighted the significant proteins within this dataset that have previously been reported as a cancer biomarker and/or drug target. In total, the AS panel contains 12 biomarker/drug target proteins and the Hx panel contains 10. Significant proteins were also compared with exosome identifications in the Vesiclepedia and ExoCarta databases. Cellular communication through exosomes or other extracellular vesicles is thought to have profound biological effects as these vesicles not only mediate classical receptor-ligand interactions, but also deliver factors that are thought to promote cancer progression [26]. All proteins in the Hx panel and 108 (98%) of the proteins in the AS panel were found in known exosome proteomes of human-origin samples analyzed by experimental methods that include mass spectrometry. Within the AS protein panel, the most highly represented ‘up-regulated’ pathways were those associated with Wnt signaling, the TCA cycle, Gonadotropin releasing hormone receptor activity and inflammation (Figure 6). This is not surprising considering that angiogenesis and altered regulation of the cell cycle are considered to be hallmarks of cancer metastasis [27]. The most highly represented ‘down-regulated’ pathways are those associated with purine synthesis, angiogenesis, FGF signaling and VEGF signaling (Figure 6). In addition, the majority of proteins within this panel are classified as hydrolase, transferase, oxidoreductase and nucleic acid binding proteins (Figure 6). Such protein types are likely to have a role in some of the other key events associated with cancer progression, including focal adhesion, extracellular matrix-receptor interactions and immune cell recruitment [27]. For the Hx panel, the most highly represented up-regulated pathways include those associated with Huntington disease and integrin signaling, (Figure 7), while the most highly represented ‘down-regulated’ pathways were those related to cytoskeletal regulation, endothelin signaling and ubiquitination (Figure 7). The majority of proteins were classified as nucleic acid binding proteins (Figure 7). These data indicate that features of the tumor itself (androgen sensitivity) and specific aspects of the tumor microenvironment (hypoxia) evoke marked alterations in signaling activity in PCa cell lines.

A data mining analysis of the mRNA expression of selected proteins using the Oncomine gene expression array datasets revealed that, in previous studies with large clinical cohorts, 11 proteins (7 AS and 4 Hx) are significantly differentially expressed between cancerous and non-cancerous prostate tissue. Furthermore, analysis of multi-cancer statistics conducted by multiple different groups indicates that 10 proteins (7 AS and 3 Hx) show a statistically significant association with PCa, as opposed to other cancer types.

### External validation of selected proteins of interest

**Figure 8:**
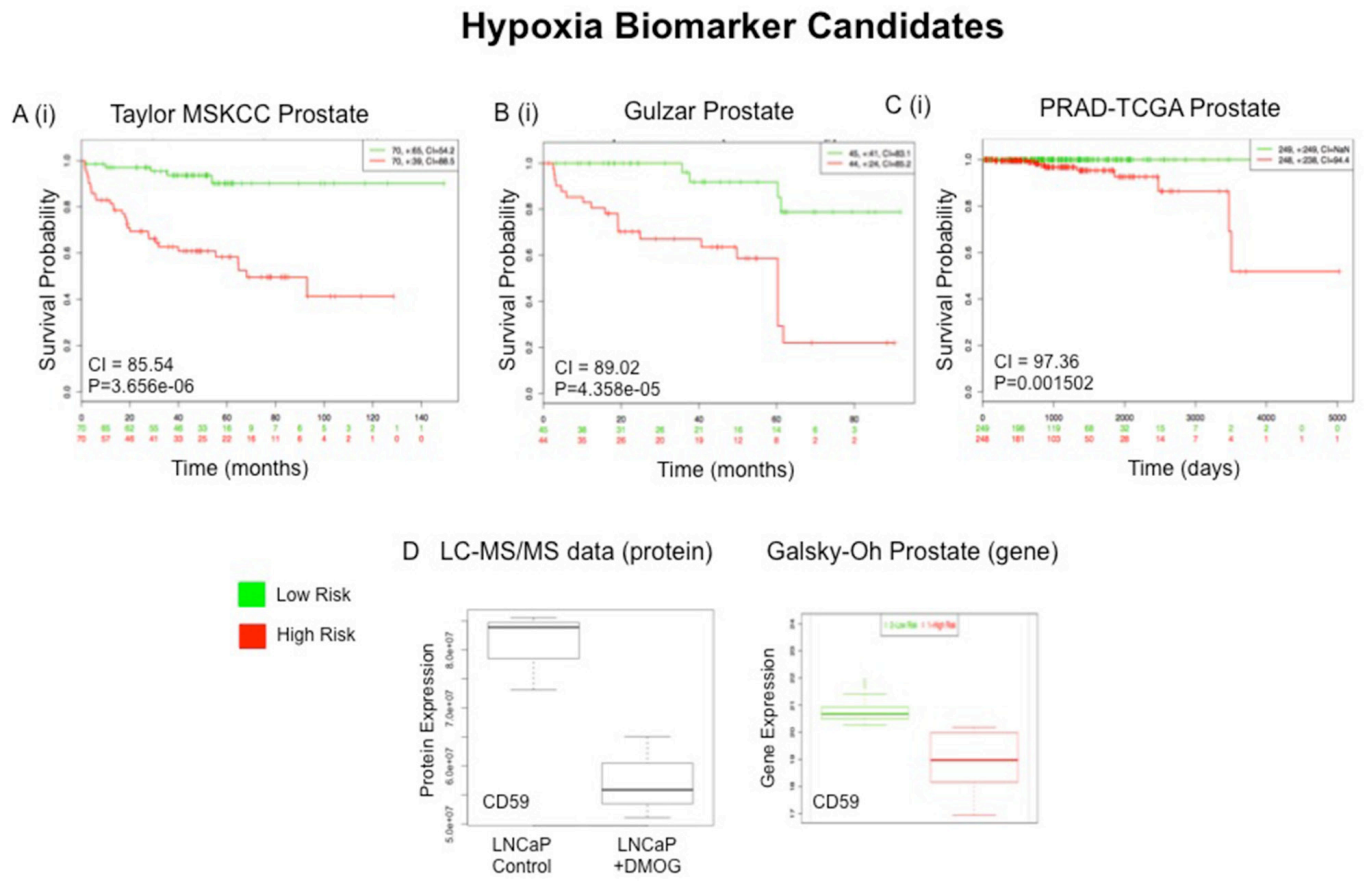
External Validation of Hypoxia (Hx) Protein Panel Top scoring proteins in the Hx protein panel (n=29) were queried through the Taylor MSKCC **A**, Gulzar **B** and PRAD-TCGA **C** prostate databases using the SurvExpress bioinformatics resource. The Prognostic value of all 29 proteins was assessed **A(i)-C(i)**. Gene expression changes of CD59, which were measured in whole blood samples in the Galsky-Oh prostate database, was compared to proteomic expression changes of CD59 as measured by LC-MS/MS analysis **D**.

To evaluate the potential prognostic capabilities of selected proteins, SurvExpress [28] was used to analyse a selection of significant proteins from both panels. The SurvExpress bioinformatics resource has previously been applied in research for the external validation of the prognostic value of panels of genes/proteins [29]. It includes data from 8 different prostate cancer datasets containing a total of 1723 samples. The 8 prostate cancer datasets described in this section were established by independent gene-based investigations of PCa progression using clinical samples (both blood and tumour tissue) by various research groups (Table 4). Clinical endpoints for these studies included disease recurrence, Gleason grade, disease stage, PSA levels and overall survival. For this study we availed of the datasets that contained a minimum of 30 samples, allowing validation of the protein panels in 6 independent clinical datasets containing a combined total of 1,673 samples (Table 4). It has been reported that molecular signatures of tumour response to hypoxia would be useful for stratifying patients based on disease prognosis [30]. As such, the ‘hypoxia-associated’ proteins identified in this study were evaluated for their potential association with PCa prognosis. A prioritized selection of 26 Hx proteins was identified according to crieteria described in the Materials and Methods and searched through the SurvExpress bioinformatics resource. All 26 proteins were identified in 3 of the 6 databases - Taylor MSKCC prostate (140 samples) [31], Gulzar Prostate (98 samples) [32] and PRAD-TCGA-Prostate adenocarcinoma (497 samples) [33] (Table 5). Of interest, down regulation of the protein CD59 glycoprotein (CD59) was found to be significantly associated with increased risk of PCa recurrence and PCa metastasis in both the Galsky-Oh [34] and Kollmeyer-Jenkins [35] PCa databases. In agreement with this, the LC-MS/MS analysis of PCa cell lines reported here, revealed a significant decrease in expression of CD59 glycoprotein in the androgen sensitive LNCaP cell line after 24 hour treatment with DMOG (Figure 8).

**Table 4:** SurvExpress Analysis of Top Scoring Hx and AS proteins

Database	Samples	Clinical Data	Matching Genes (Hx)	CI (Hx)	Survival ROC (Hx)	Matching Genes (AS)	CI (AS)	Survival ROC (AS)
**Taylor MSKCC Prostate**	140	Recurrence, Gleason, Stage	26/26	85.54	0.83	51/51	89.13	0.90
**Galsky Oh- Prostate - GSE45705**	61	Survival	1/26	54.41	0.66	1/51	53.75	0.56
**Sboner Rubin Prostate GSE16560**	281	Gleason	20/26	66.31	0.74	41/51	70.07	0.81
**Gulzar-Prostate-GSE40272**	98	Recurrence	26/26	89/02	0.90	51/51	99.83	1.02
**Kollmeyer-Jenkins Prostate GSE10645-GPL5858**	596	Survival, Age, PSA, Stage, Grade	1/26	61.06	0.68	3/51	72.74	0.82
**PRAD - TCGA - Prostate adenocarcinoma June 2016**	497	Survival	26/26	97.36	0.93	50/51	99.95	0.98

**Table 5:** Top Scoring Proteins in Hypoxia Panel

Top Proteins: Hypoxia Panel										
Accession Number	Protein IDs	t-test difference	%CV SR	%CV TR	Where Significant	Biomarker/Drug Target	SignalP/Phobius	ExoCarta/Vesiclepedia	Oncomine	Score
**P14174**	MIF_HUMAN	-1.092	3.7	23.2	Hof 8 Hour	BM		✔	✔	4
**Q969N2-5**	PIGT_HUMAN	1.317	5.6	24.7	Hof 8 Hour	BM	✔	✔		3
**P04075**	ALDOA_HUMAN	-1.600	1.4	2.1	Abl 24 Hour		✔	✔		3
**P05114**	HMGN1_HUMAN	-1.069	20.3	33.1	Abl 8 hour	BM		✔	✔	3
**Q14573**	ITPR3_HUMAN	-0.966	5.0	4.5	Hof 24 Hour			✔	✔	3
**O00468-6**	AGRIN_HUMAN	1.099	10.7	1.0	Abl 24 Hour		✔	✔		2
**Q9H488**	OFUT1_HUMAN	-1.501	14.9	2.2	Abl 24 Hour		✔	✔		2
**O43291**	SPIT2_HUMAN	1.698	4.0	7.3	Abl 24 Hour		✔	✔		2
**Q02809**	PLOD1_HUMAN	-1.834	6.5	7.6	Abl 24 Hour		✔	✔		2
**Q10471**	GALT2_HUMAN	0.604	13.0	8.3	Hof 8 Hour		✔	✔		2
**Q7L5N1**	CSN6_HUMAN	-1.710	11.3	9.8	Hof 8 Hour		✔	✔		2
**P13987**	CD59_HUMAN	1.554	8.5	10.0	LNCaP 24 Hour		✔	✔		2
**Q03252**	LMNB2_HUMAN	-0.852	1.1	10.0	Hof 8 Hour		✔	✔		2
**Q5T653**	RM02_HUMAN	-2.183	9.0	11.0	LNCaP 8 Hour		✔	✔		2
**Q969H8**	CS010_HUMAN	-1.019	3.0	11.0	Hof 8 Hour		✔	✔		2
**P43304**	GPDM_HUMAN	0.202	28.3	15.9	Abl 8 hour		✔	✔		2
**P14314-2**	GLU2B_HUMAN	-1.481	2.8	18.4	Hof 8 Hour		✔	✔		2
**Q7Z7H5**	TMED4_HUMAN	0.596	11.5	19.3	Hof 8 Hour		✔	✔		2
**A0AVT1**	UBA6_HUMAN	-1.182	14.9	1.9	Abl 24 Hour	BM		✔		2
**Q7KZF4**	SND1_HUMAN	1.398	2.2	4.1	LNCaP 24 Hour	BM		✔		2
**Q12792**	TWF1_HUMAN	-0.939	21.0	5.8	Abl 24 Hour	BM		✔		2
**P12277**	KCRB_HUMAN	-0.991	14.3	7.3	Abl 24 Hour	BM		✔		2
**P02144**	MYG_HUMAN	-1.494	12.8	7.6	LNCaP 8 Hour	BM		✔		2
**P13861**	KAP2_HUMAN	-1.741	8.6	10.8	Hof 24 Hour	BM		✔		2
**Q9NP58-4**	ABCB6_HUMAN	0.553	8.6	6.9	Hof 24 Hour	BM, DT		✔		2
**P26358**	DNMT1_HUMAN	1.399	18.3	7.6	Hof 24 Hour			✔	✔	2
**Q92597**	NDRG1_HUMAN	-1.885	26.9	19.3	Abl 24 Hour			✔		2

**Figure 9:**
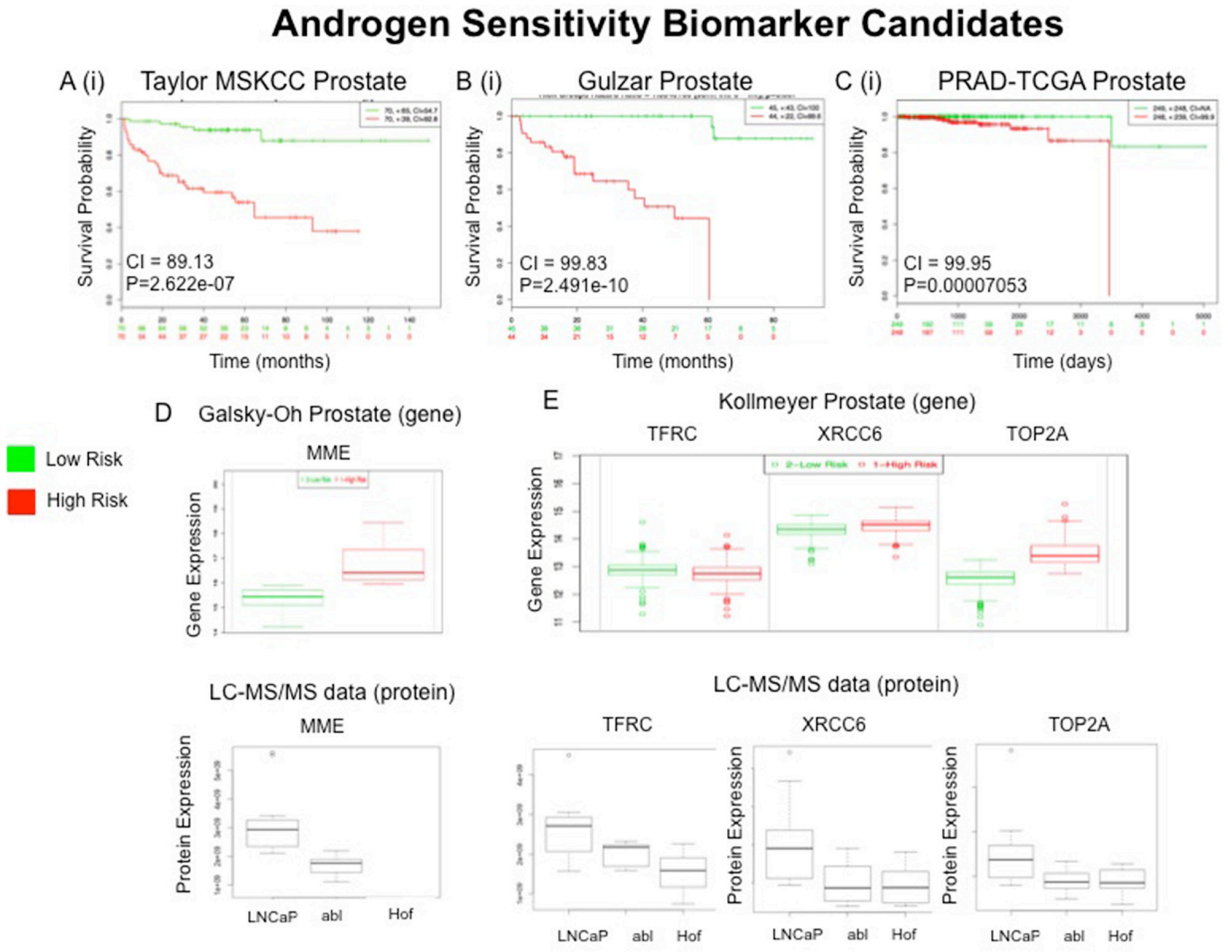
External Validation of Androgen Sensitivity (AS) Protein Panel Top scoring proteins in the AS protein panel (n=51) were queried through the Taylor MSKCC **A**, Gulzar **B** and PRAD-TCGA **C** prostate databases using the SurvExpress bioinformatics resource. The Prognostic value of all 51 proteins was assessed **A(i)-C(i)**. Gene expression changes of MME, which was measured in whole blood samples in the Galsky-Oh prostate database, were compared to proteomic expression changes of MME as measured by LC-MS/MS analysis **D**. Gene expression changes of TFRC, XRCC6 and TOP2A, which were measured in tissue samples in the Kollmeyer prostate database, were compared to proteomic expression changes of TFRC, XRCC6 and TOP2A as measured by LC-MS/MS analysis **E**.

For the AS panel a total of 51 proteins were prioritized and searched through the SurvExpress bioinformatics resource (Table 5). All 51 of the top ranked proteins were identified in 2 of the 6 databases - Taylor MSKCC prostate (140 samples) [31] and Gulzar Prostate (98 samples) [32] (Table 4). The protein MME was found to have a strong association with risk of CRPC in the Galsky-Oh database. The Galsky-Oh database reports an increase in MME expression in association with high risk PCa while our data indicate an increased MME expression in the androgen sensitive (LNCaP) cell line as opposed to the androgen independent (Abl and Hof) cell lines. The proteins TFRC, XRCC6 and TOP2A were found to be strongly associated with risk of PCa recurrence and metastasis in the Kollmeyer-Jenkins database. In the associated study it was reported that XRCC6 and TOP2A expression was increased in high risk PCa, while the LC-MS/MS dataset acquired in this study showed increased expression of XRCC6 and TOP2A in the androgen sensitive cell line. In the Kollmeyer-Jenkins database it was also reported that TFRC expression was increased in high risk PCa, while the LC-MS/MS data reported here revealed increased expression of TOP2A in the androgen independent cell lines (Figure 9).

### Label-free normalisation of MRM data

In order to observe true changes in protein expression between samples as result of experimental conditions it is important to normalize analytical samples based on their total protein concentration. Although samples analysed in this study were zip-tipped prior to analysis with zip-tips that have a maximum peptide capacity of 5 μg, this does not provide sufficient confirmation that an equal amount of protein will be injected onto the mass spectrometer for each individual sample. Generally, such variability is corrected for by measuring ‘house-keeping’ proteins such as GAPDH or alpha-Tubulin. However, it is entirely feasible that the expression of traditionally measured house-keeping proteins, as with all proteins, can be altered as result of exposure to various external stimuli [36, 37] – Indeed GAPDH is known to be differentially expressed under hypoxic conditions [34]. In 2014 Eisenberg et al published an analysis of expression data from the Human BodyMap (HBM) 2.0 project, which includes publically available RNAseq data (GEO accession number G3E30611, HBM) of 16 normal human tissue types – including prostate. From this analysis Eisenberg et al identified 3804 ‘house-keeping’ genes which exhibited a uniform expression level across all tissues [38]. The full list of housekeeping genes is publicly available and was downloaded as a reference source for the identification of suitable housekeeping proteins for appropriate normalization of MRM data in our verification studies. To select suitable proteins against which to normalize for total protein concentration the LC-MS/MS data was assessed to identify proteins that were identified in all cell lines under both control and hypoxic conditions with CV less than 20%. These were cross-referenced against the list of housekeeping genes identified by Eisenberg et al. The 17 most abundant proteins that were commonly identified at both time points and in the housekeeping gene databse were used for MRM-based normalization measurements.

### MRM design for evaluation of selected proteins of interest

As a strategy to verify the changes observed from label-free LC-MS/MS-based analysis conducted for this study, multiple reaction monitoring (MRM) offers the advantage of allowing multiplexed, high throughput measurement of large numbers of proteins (up to 100) without the need for antibodies [39, 40]. MRM assays for all selected proteins were designed using Skyline (version 3.5) software. The process of selecting peptides generated by tryptic digestion of each target protein is of critical importance to MRM assay design. It is crucial to select peptides with favorable mass spectrometry properties as this will determine the sensitivity of the assay [41]. Proteotypic peptides for each protein were therefore selected according to pre-defined criteria (see Materials and Methods). Proteins were also searched in Peptide Selector and SRM Atlas to identify peptides for which MRM assays have previously been developed. The aim of these assays was to validate the significant changes in protein expression observed from analysis of the discovery data. The assays were thus designed to measure those peptides which show a significant change as result of either hypoxia or androgen sensitivity. This was determined based on one-way ANOVA analysis of measured LFQ intensity for all identified peptides. As a means of predicting which peptides would be detectable in the triple quadrupole mass spectrometer (Agilent 6490), peptides that had previously been identified in the similarly designed Q-Tof mass spectrometer (6550) were given priority. Implementation of these selection criteria ensures that efforts to verify changes in protein expression by MRM are based on peptide measurements that actually reflect protein changes observed in the discovery phase and are likely to provide high quality, reproducible data. Transition lists for the Hx and AS panels are shown in Tables 3 and 4 of the Supplementary Data.

## DISCUSSION

In this study we have applied a robust, large-scale mass-spectrometry based approach to elucidate and characterize the proteome of androgen sensitive and androgen independent PCa cell lines under hypoxic and normoxic conditions. The resulting data represents one of the largest datasets of proteins identified from label-free LC-MS/MS analysis of PCa cell lines. All interpretations of the significant changes in protein expression reported here are permissible by the excellent reproducibility observed between the sample replicates, technical replicates and biological replicates that were analysed as part of our experimental workflow (Figure 2-3). The quality of these data can therefore be attributed to the investment made in careful planning of the experimental workflow at all stages throughout this study – a fundamental requirement for translating any potential discoveries into clinical relevance [42].

Hypoxic stress is one of the most common stressors encountered within the PCa tumour microenvironment and ultimately results in the transformation of cancer cells into a more aggressive phenotype [43]. The hypoxic tumour microenvironment is known to correlate with increased tumour invasiveness, metastasis and resistance to radiotherapy and chemotherapy which, consequently, makes it a poor prognostic indicator for patients with PCa [8, 44]. Although Hif-1 α is generally used as a biomarker for hypoxia, it is an unstable protein and it's expression level may not always directly correlate with hypoxia. Thus, in order to predict the efficacy of therapies which are directly impacted by the oxygen content of the tumour microenvironment, such as radiation therapy, there is a need for alternative or complimentary hypoxia biomarkers [43]. As with most biomarker-related studies, it is now widely accepted that panels of proteins offer more value than individual proteins in predicting disease outcomes and this has been reflected in the reported efforts to define a signature of hypoxia in cancer cells. The hypoxia signatures which have been reported to date show great variety but little commonality [30]. This may be down to the extreme spatial and temporal heterogenesis in tissue oxygen levels, which cannot be accurately captured with current analytical methods [45]. Buffa *et al* conducted a meta-analysis of all studies relating to the identification of hypoxia biomarkers and subsequently compiled a list of the top-ranked common genes identified across all selected studies [46]. A number of these proteins - n-myc downstream regulated gene 1, aldolase A and macrophage migration inhibitory factor – were identified in this study and are included in the panel of hypoxia-related proteins that were short-listed for further verification.

In PCa, the evolution from a clinically localized, hormone-naïve state to a castrate resistant phenotype is largely attributed to inappropriate restoration of androgen signaling activity [47]. In this study, it was observed that approximately 300 and 500 proteins were significantly changed between the androgen sensitive and androgen independent cell lines at 8 hour and 24 hour time points, respectfully. Pathway analysis of the significantly changing proteins revealed that the top deregulated signaling pathways were the AMP-activated protein kinase (AMPK) signaling pathway, the Interleukin-3 (IL-3) signaling pathway and androgen receptor (AR) signaling pathway (Table 2). The primary role of AMPK signaling is to orchestrate the global metabolic adaption of cells to energy crisis and oxidative stress [48]. There is accumulating evidence to suggest that PCa tumour cells may increase their resistance to stress by up-regulating AMPK activity [49]. Targeting of the AMPK signaling pathway has therefore been extensively explored and it has been shown that activation of AMPK by metformin causes a decrease in AR protein levels through suppression of mRNA expression and promotion of AR protein degradation [50, 51]. However, overexpression of AR can delay AMPK activation and increase PCa cellular resistance to metformin treatment which suggests that AR suppresses AMPK signaling-mediated growth inhibition through a negative feedback loop [51]. In fact there have been studies which report AR-mediated PCa cell growth via AMPK [52, 53]. Thus it is not surprising that AMPK and AR make up two of the top three deregulated pathways between androgen sensitive and androgen independent cell lines and our data is therefore in agreement with the many previous studies which advocate targeting these pathways as a strategy for treatment of CRPC [9, 50, 54–56]. Furthermore, our analysis identifies the proteins that contribute most significantly to this deregulation and these proteins may have potential use as either biomarkers or therapeutic targets for PCa. The third pathway that was identified as being significantly deregulated between androgen sensitive and androgen independent PCa cell lines was the IL-3 signaling pathway. The main role of IL-3 is to support differentiation and proliferation of various immune cells, thereby giving it a significant role in the immune response [57]. It has been shown that signals emanated by IL-3 are crucial for tumor cell proliferation and migration and that blockage of IL-3 can prevent both tumor angiogenesis and growth [58]. Indeed there is evidence to suggest that targeting the IL-3 receptor within BCR-ABL-driven lymphoid leukemias represents a viable therapeutic strategy [59]. As such, the proteins identified here which contribute to the significant deregulation of the IL-3 signaling pathway may also be worthy of further investigation as both biomarkers and therapeutic targets for CRPC.

External validation of proteins of interest in the Hx and AS panels appear to indicate strong correlation between their respective gene expression and PCa progression (Table 4). Previous work has demonstrated that panels of MRM assays can effectively contribute to biological characterisation of molecular subtypes of cancer [60]. The preliminary in silico validation of selected proteins identified in this study also suggests greater prognostic value in the multiplexed measurement of protein panels as opposed to individual protein measurements. One of the more notable datasets included in this validation was that of the Galsky-Oh PCa database. The Galsky-Oh database is the only to have come from a study in which blood samples from patients with CRPC were used in the analysis. This is a particularly relevant dataset to this study for two important reasons: (i) the main objective of the study we have reported here was to investigate features of the tumour microenvironment that may lead to the development of CRPC, and (ii) the comprehensive dataset we have acquired should support the identification of molecular signatures of hypoxia and/or loss of androgen sensitivity that ultimately could be used to stratify patients. De-regulated expression of the proteins MME, TFRC, XRCC6, TOP2A and CD59 were found to be significantly associated with increased risk of PCa recurrence in the Galsky-Oh study and also identified as significantly changed in expression between androgen sensitive and androgen independent cell lines (MME, TFRC, XRCC6 and TOP2A) and as result of hypoxia (CD59). Proteins that are detectable in blood are potentially the most useful in a clinical diagnostic setting. Therefore, it is highly promising that some of the proteins identified here have previously been detected and measured in blood samples from patients with CRPC.

TOP2A is a widely used target of existing anti-cancer drugs and it's expression is often used as a cancer cell marker because of it's role in cell proliferation [61]. Indeed, it is the only protein identified in our dataset that is targeted by currently FDA approved drugs for PCa. Etoposide in combination with estramustine was previously recommended by the National Comprehensive Cancer Network (NCCN) as a standard of care for treatment of CRPC. Other TOP2A poisons, such as mitoxantrone and doxorubicin are also occasionally prescribed to treat CRPC. However, treatment of PCa with TOP2A poisons has been shown to provide only palliative benefits and current clinical data has not yet determined the most beneficial use of targeting TOP2A in patients with advanced PCa [62, 63]. Our data would suggest that, while the association of TOP2A expression and advanced PCa cannot be disputed (Figure 9), it may not be directly related to the androgen sensitivity of PCa cells. A similar observation has been made previously in which it was observed that although cells which showed high levels of TOP2A were highly proliferative and associated with metastasis in PCa, cancer stem cells were actually enriched in PCa cells which showed low/negligible levels of TOP2A. As such, these researchers argue that therapies targeted against TOP2A^neg^ cells may be of greater benefit to patients with advanced PCa [64].

Although cell lines represent the most reductionist model of cancer available the simplicity and accessibility of PCa cell lines permits the easy reproduction of research findings in different laboratories. They are also considered an extremely useful model for identifying prospective gene/protein targets in a fast and efficient manner – a key requirement for this work [65]. We do, however recognize that accurately epotimizing the *in vivo* tumour microenvironment in a 2D cell culture system is effectively impossible. The Previously, we have applied workflows similar to those described here for proteomic profiling of the PCa tumour microenvironment using laser capture microdissected regions of patient tumour tissue [66]. Reassuringly, there is significant overlap between changing proteins identified in this cell-based study and previous tissue-based studies (data not shown), thus providing confidence that our cell line system represents a faithful model of the *in vivo* tumour microenvironment. External validation of a number of the proteins in both the Hx and AS panels using publicly availably databases has shown that, although a cell line model was used for this study, the proteins identified as significant are of clinical relevance and, importantly, have previously been measured in both tissue and blood samples from patients.

Biological and clinical evaluation of proteins selected for the As and Hx panels have provided a strong indication that the proteins identified in this study are worthy of further mechanistic and functional investigation. Notably, the role of the androgen signaling and HIF signaling pathways in regulating these proteins should be verified. For example, it would be of interest to investigate the extent to which the addition of androgen (and other manipulations) to the cell lines will alter the expression of AS proteins. Likewise, the true relvence of Hx proteins identified in this study to *in vivo* tumour hypoxia is worthy of further investigation. This could be achieved by analysis of the expression of Hx proteins in various models of hypoxic conditions such as cell growth under low O_2_, treatment with cobolt chloride [67] or treatment with desferrioxamine [68]. To facilitate these important studies and others like it, high throughout multiplexed MRM assays were designed as a means of verifying of protein expression changes under varyious experimental conditions. Successful studies have previously been undertaken by our group in which MRM assays were deigned for the measurement of protein panels with potential prognostic value for PCa and other inflammatory diseases [69–71]. Here MRM assays were primarily designed to measure proteotypic peptides that showed a significant fold change between either androgen independent and androgen sensitive PCa cell lines or as result of hypoxia, To control for potential variances in total protein concentration the acquired LC-MS/MS discovery data was used to identify the most appropriate housekeeping proteins for normalization of the MRM measurements. These MRM assays provide a label-free, experiment-specific means of normalizing the MRM data that will be acquired during the evaluation of AS and Hx proteins. When applied as part of well-designed experiments, these robustly designed assays will provide a reliable means of investigating the functional role of Hx and AS proteins in CRPC.

Thus far the large dataset acquired in this study has highlighted some of the major proteomic changes occurring as result of hypoxia and/or androgen sensitivity in PCa cell lines and allowed us to (i) gain further biological insight into the PCa tumor microenvironment, (ii) identify potential protein biomarkers that may be indicative of treatment resistant PCa (CPRC) and/or hypoxia and (iii) identify potential drug targets for therapeutic intervention in PCa. Moreover, the findings reported here appear to be consistent with genomic data from independent cohorts of clinical (blood and tissue) samples. The results described will be confirmed by further analysis in both cell line and clinical samples using the MRM assays designed as part of this study.

**Table 6:** Top Scoring Proteins in Androgen Sensitivity Panel

Top Proteins: Androgen Sensitivity Panel										
Accession Number	Protein IDs	%CV SR	%CV TR	Where Significant	AIvAS	BM/DT	Signal IP/Phobius	ExoCarta/Vesiclepedia	Oncomine	Score
**Q8NBS9**	TXND5_HUMAN	5.2	16.3	8 Hour AS v AI	Up		✔	✔	✔	4
**P27797**	CALR_HUMAN	4.4	5.1	8 Hour AS v AI, AR	Up	BM	✔	✔		4
**P11388**	TOP2A_HUMAN	6.8	7.0	AS v AI (IPA)	Down	BM, DT		✔	✔	4
**Q05655**	KPCD_HUMAN	35.0	40.6	8 Hour AS v AI, IL-3, AR	Down	DT		✔		3
**P07237**	PDIA1_HUMAN	2.4	0.7	8 Hour AS v AI	Up		✔	✔		3
**Q92820**	GGH_HUMAN	10.4	2.9	8 Hour AS v AI	Up		✔	✔		3
**P07099**	HYEP_HUMAN	27.1	3.9	24 Hour AS v AI	Up		✔	✔		3
**P49321**	NASP_HUMAN	13.1	5.2	8 Hour AS v AI	Up		✔	✔		3
**O00116**	ADAS_HUMAN	6.2	5.3	8 Hour AS v AI	Up		✔	✔		3
**Q8TEM1**	PO210_HUMAN	5.4	7.9	8 Hour AS v AI	Up		✔	✔		3
**O75795**	UDB17_HUMAN	6.9	8.2	8 Hour AS v AI	Up		✔	✔		3
**P30533**	AMRP_HUMAN	18.2	8.5	24 Hour AS v AI	Up		✔	✔		3
**Q9UM54-6**	MYO6_HUMAN	4.9	3.2	8 Hour AS v AI	Up			✔	✔	3
**P27144**	KAD4_HUMAN	18.3	4.3	AMPK	Down			✔	✔	3
**P13807-2**	GYS1_HUMAN	47.5	19.6	AMPK	Down			✔	✔	3
**Q08209-2**	PP2BA_HUMAN	8.6	5.2	IL-3	Down	DT		✔	✔	3
**P02786**	TFR1_HUMAN	5.8	1.1	8 Hour AS v AI	Down			✔		2
**O95573**	ACSL3_HUMAN	5.9	2	24 Hour AS v AI	Down			✔		2
**O60313**	OPA1_HUMAN	6.6	2.6	8 Hour AS v AI	Down			✔		2
**P12956**	XRCC6_HUMAN	2.8	2.7	8 Hour AS v AI	Down			✔		2
**Q16762**	THTR_HUMAN	10.0	2.9	8 Hour AS v AI	Down			✔		2
**Q16222-3**	UAP1_HUMAN	15.1	3.0	8 Hour AS v AI	Down			✔		2
**Q14739**	LBR_HUMAN	15.2	3.0	8 Hour AS v AI	Up			✔		2
**Q8WVV9-5**	HNRLL_HUMAN	4.1	3.1	8 Hour AS v AI	Up					2
**P21333-2**	FLNA_HUMAN	10.1	4.2	8 Hour AS v AI	Down			✔		2
**Q9H2U2**	IPYR2_HUMAN	6.3	4.4	8 Hour AS v AI	Up			✔		2
**P17858**	PFKAL_HUMAN	20	4.6	AMPK	Up			✔		2
**P08473**	NEP_HUMAN	5.1	4.7	8 Hour AS v AI	Down			✔		2
**Q6PKG0**	LARP1_HUMAN	6.3	5.0	8 Hour AS v AI	Down			✔		2
**Q9Y678**	COPG1_HUMAN	8.7	5.6	8 Hour AS v AI	Up			✔		2
**P33121**	ACSL1_HUMAN	5.6	5.9	8 Hour AS v AI	Up			✔		2
**O75607**	NPM3_HUMAN	15.6	6.0	24 Hour AS v AI	Down			✔		2
**P55060-3**	XPO2_HUMAN	1.4	6.1	8 Hour AS v AI	Down			✔		2
**Q53H82**	LACB2_HUMAN	9.8	6.2	8 Hour AS v AI	Up			✔		2
**Q6DD88**	ATLA3_HUMAN	2.5	6.3	8 Hour AS v AI	Up			✔		2
**P07195**	LDHB_HUMAN	4.3	7.0	8 Hour AS v AI	Down			✔		2
**Q13228**	SBP1_HUMAN	8.7	7.0	8 Hour AS v AI	Up			✔		2
**Q9UBQ7**	GRHPR_HUMAN	6.9	7.1	24 Hour AS v AI	Down			✔		2
**Q13813-3**	SPTN1_HUMAN	6.4	7.2	8 Hour AS v AI	Up			✔		2
**Q1KMD3**	HNRL2_HUMAN	5.0	7.6	8 Hour AS v AI	Up			✔		2
**O15020-2**	SPTN2_HUMAN	6.1	7.7	8 Hour AS v AI	Up			✔		2
**Q6NVY1**	HIBCH_HUMAN	15.4	7.8	8 Hour AS v AI	Up			✔		2
**P49915**	GUAA_HUMAN	5.6	8.4	8 Hour AS v AI	Down			✔		2
**Q96HC4**	PDLI5_HUMAN	10.4	8.6	8 Hour AS v AI	Down			✔		2
**P08133**	ANXA6_HUMAN	0.9	10.6	8 Hour AS v AI	Up			✔		2
**P35580**	MYH10_HUMAN	1.7	10.8	8 Hour AS v AI	Down			✔		2
**P35573**	GDE_HUMAN	10.3	11.0	8 Hour AS v AI	Up			✔		2
**Q14938-5**	NFIX_HUMAN	4.1	11.9	8 Hour AS v AI	Up			✔		2
**Q9H2U1**	DHX36_HUMAN	14.8	13.1	8 Hour AS v AI	Down			✔		2
**O15355**	PPM1G_HUMAN	20.2	15.2	AMPK	Down			✔		2
**P30837**	AL1B1_HUMAN	3.6	15.8	8 Hour AS v AI	Down			✔		2
**Q05639**	EF1A2_HUMAN	18.1	16.1	24 Hour AS v AI	Down			✔		2

## MATERIALS AND METHODS

### Cell culture

The PCa cell lines, LNCaP, LNCaP-abl (Abl) and LNCaP-abl-Hof (Hof) were gifted to the Irish Prostate Cancer Research Consortium, Dublin, Ireland from the laboratory of Professor Helmut Klocker (Department of Urology, University of Innsbruck, Austria). Culturing of the above cell lines was conducted in a class II laminar flow cabinet. Cells were maintained in T175cm2 flasks with ventilation (Starsted) in a 5% CO2 humidified atmosphere at 37°C. LNCaP cells were maintained in Advance RPMI 1640 media (GIBCO Life Technologies) and supplemented with 10% foetal calf serum (FCS) (Sigma-Aldrich), 2μM/ml L-Glutamine (GIBCO Life Technologies), 50 unit/ml Penicillin and 50μg/ml Streptomycin (GIBICO Life Technologies). Abl and Hof cells were maintained in Advance RPMI 1640 media supplemented with 10% charcoal stripped FCS (Sigma-Aldrich), 2μM/ml L-Glutamine (GIBCO Life Technologies), 50 unit/ml Penicillin and 50μg/ml Streptomycin (GIBICO Life Technologies). For the three cell lines media was changed every 3-4 days.

### Simulation of hypoxia in PCa cell lines

LNCaP, Abl and Hof cell lines were seeded into 10 cm^2^ culture dishes and grown to 70-80% confluence. For each cell line, media was removed and replaced with appropriate media supplemented with 1mM dimethyloxaloglycine (DMOG; Cambridge Bioscience) or 1mM dimethyl sulfoxide (DMSO; Sigma Aldrich) as a control. Cells were incubated in treated media for either 8 or 24 hours prior to cell lysis. To reduce analytical variation and false positive results while ensuring sensitivity and reproducibility, three independent biological replicates were generated for this proteomic study.

### Cell lysis and western blot analysis

Adherent cells were washed twice with ice-cold PBS and removed from cell culture plates by scraping. Cells were transferred into eppendorf tubes and spun for 5 min at 3,000 *g* at 4°C. PBS was removed and cell pellet was lysed by sonication in 100 μl 1% sodium dodecyl sulfate (SDS; Sigma Aldrich). Samples were heated at 95°C for 5 min to encourage denaturation, and subsequently centrifuged at 14,000 *g* for 10 min at 4°C to remove cell debris. Protein concentration of cell lystates was measured by BCA assay according to manufacturer's instructions (Pierce). A 2 μg stock solution of Bovine Serum Albumin (BSA) (Sigma-Aldrich) was used to prepare a series of standards (2000, 1500, 1000, 750, 500, 250, 125, 25, 0 μg/ μl) which were diluted to appropriate concentrations with ddH_2_O. 200 μl of BCA working solution (50:1 BCA reagent A: BCA reagent B) was added to 25 μl of each sample/standard in a 96 well plate. Each sample was measured in triplicate and absorbance was read at 540nm on a SpectraMax M2 [D05029] microplate reader (Molecular Devices). For western blot analysis of Hif-1α expression, 30 μg whole cell lysate was diluted 1:1 with SDS sample buffer (400 mM Tris-HCL [pH 6.8], 30% glycerol, 10% SDS, 0.2 M DTT, 0.02% Coomassie blue G-250) and samples were denatured by heating at 95°C for 5 min. Protein samples were separated by SDS-PAGE using in house made 12% polyacrylamide gels (Protogel-National Diagnostics) run on Mini-PTOTEAN ® Tetra Handcast Systems (Bio-Rad). Denatured samples were loaded into the wells of 4% stacking gels using a micropipette. 4 μl of Precision Plus Protein dual colour marker (Bio-Rad) was loaded into the first lane of each gel to allow determination of relative molecular weight of immunoblotted proteins. Electrophoresis was undertaken using 1 X running buffer (0.025 M Tris, 0.192 M glycine and 1% SDS) at 40 V initially, and 140 V once samples had passed into the resolving gel layer. Following electrophoresis, separated proteins were transferred onto PVDF transfer membranes (PerkinElmer) using a wet mini-transfer apparatus (Bio-Rad). Electrophoretic transfer was carried out on ice in transfer buffer at a constant voltage of 100 V for 90 min. PVDF membranes were incubated for 120 min at room temperature in blocking buffer containing 5% non-fat milk (Sigma-Aldrich), 0.05% Triton 100 in PBS. After blocking, membranes were incubated overnight at 4°C with primary antibody diluted as required in 5% non-fat milk blocking buffer. Membranes were then washed 5 times for 5 min in washing buffer (0.05% Triton 100 in PBS; PBS-T) and incubated for 90 min at 4°C (dark) with the anti-mouse horseradish peroxidase (HRP) conjugated secondary antibody (Santa Cruz) diluted 1:1000 in 5% non-fat milk blocking buffer. Membranes were then washed 5 times for 5 min in washing buffer and incubated in ECL Western Blotting Substrate (Medical Supply). Protein expression was detected by exposure on Fuji medical X-Ray films (Fujifilm Global) for between 1 and 10 mins. Primary antibodies used for immunoblot analysis were Hif-1 α antibody (#610958 BD Biosciences – diluted 1:500) and α-Tubulin (#5546 Santa Cruz Biotechnology – diluted 1:1000). The secondary antibody used was anti-mouse IgG (heavy and light chain) HRP conjugated (#7074 Cell Signalling Technology).

### Sample preparation for nLC-MS/MS analysis

Whole cell lysates were prepared for nLC-MS/MS analysis according to the filter aided sample preparation (FASP) method as described by Wisniewski et al (AK 49). Briefly, 50 μg cell lysate proteins were reduced through boiling (95°C for 5 min) with DTT in a final concentration of 0.1 M. 200 μl UA buffer (8 M Urea, 0.1 M Tris-HCL, pH 8.5) was added to each sample, and samples were transferred to 30,000 MQCO Vivacon 500 spin filters (Sartorious) and centrifuged at 14,000 *g* for 40 min, 21°C. Bound proteins were alkylated through 5 min incubation of spin filters in 0.05 M iodoacetamide (IAA) followed by centrifugation at 14,000 *g* for 30 min, 21°C. Spin filter membranes were then washed three times through addition of UB (8 M Urea, 0.1 M Tris/HCL, pH 8.0) and centrifugation at 14,000 *g* for 40 min, 21°C. For maximum protein identifications, sample protein was digested with both Lys-C (Wako Chemicals GmbH) and Trypsin (Promega) enzymes. Proteins were initially digested overnight with Lys-C (enzyme: substrate 1:50) in a wet chamber. Digestion was completed by a 3-hour incubation with Trypsin (enzyme: substrate 1:100) in a thermomixer set to 37°C, 600 rpm. Digestion was stopped by acidification of samples through addition of trifluoroacetic acid (TFA) to a final concentration of 1%. Peptide material from digested cell lysates were purified using C18 resin ZipTips ® (Millipore). Each ZipTip contains C18 resin packed into a 10 μl pipette tip with a loading capacity of 5 μg protein/peptide for tip. This allows for purification of peptide material of molecular weight between 0-50 kDa. For purification of cellular peptides, the C18 resin was activated with 10 μl acetonitrile (x10). The resin was then equilibrated by pipetting 10 μl 0.5% trifluoroacetic acid (TFA) (x10). Peptides were then bound to the resin by pipetting 15 μl of digested sample through the resin (x10). Bound peptides were eluted into fresh eppendorfs in 25 μl Elution Buffer [70% acetonitrile, 0.1% TFA] (x2). This process was repeated four times for each sample to ensure maximum yield of purified peptide for nLC-MS/MS analysis. Eluted peptides were dried down under vacuum for approximately 1 hour at 30 °C and re-suspended in 30 μl Buffer A [3% CAN, 0.1% formic acid] to allow for ≈3 μg peptide per 5 μl injection on the Q-Exactive mass spectrometer.

### nLC-MS/MS analysis

Samples were analysed by nano-flow reverse phase LC using a Q-Exactive mass spectrometer connected online to an Ultimate Ultra3000 chromatography system (both Thermo Fisher Scientific) as described [72, 73]. Briefly, dried peptides were reconstituted in 0.01 % TFA and 5 µl of each sample were loaded onto an analytical column (150 mm length, 75 µm inside diameter) packed in house with 1.9 µm ReprosilAQ C18 (Dr Maisch GmbH). Peptides were separated using a 130-minute linear gradient from 4 % to 32 % acetonitrile at a flow rate of 250 nl/min. The mass spectrometer was operated in data-dependent acquisition mode with a top-12 MS/MS scanning approach. For protein label-free identification and quantification, tandem mass spectra and peptide fragments of the 12 most abundant peaks were acquired in the linear ion trap by peptide fragmentation using higher energy collisional dissociation (HCD). A 2300 V potential was applied to column with a capillary temperature of 320 °C. Samples generated for each time point were analysed in two separate experimental runs in a randomized order which included three biological replicates for each sample, sample replicates generated pre-digestion (SR) and technical replicates digested post-sample preparation (TR).

### Data processing and statistical analysis

PEAKS (version 7) software was used to determine the number of peptides and proteins identified in each sample. Files generated from nLC-MS analysis (.d) were directly uploaded onto the PEAKS software and database searching was performed using ‘HumanUniprot’ database[39,704 protein sequences] (downloaded 01/11/2013) with the following search parameters applied: enzyme: trypsin, maximum missed cleavages: 2, species: Homo Sapiens, variable modifications: oxidation methionine, 4-hydroxynonenal (4-HNE), lysine acetylation at N and C termini, amidation, ammonia loss at N and C termini, precursor ion tolerance: 10 ppm, product ion tolerance: 0-3 and maximum variable modifciations per peptide: 3. The false discovery rate (FDR) was set to 0.1%. The raw LC-MS/MS data was then processed using MaxQuant computational proteomics platform version 1.4.1.2. Raw files were directly imported into the software and protein identifications were generated by processing the data through the in-built Andromeda search engine matched, against the Uniprot/Swissprot database [40.452 protein sequences] (downloaded 29/07/2014) with FDR set to 0.5%. Additional search parameters were as follows, enzyme: trypsin, allow up to two missed cleavages, species: Homo sapiens, fixed modification: carbimidomethylated cysteine, variable modification: oxidation methionine, minimum peptide length: 7 amino acids. The precursor mass tolerance window was set to 6 ppm and product mass tolerance was 20 ppm. A minimum of 2 peptides was required to confirm protein identification. This search provided a full list (.txt format) of peptide and protein identifications along with their respective label-free quantitation (LFQ) intensities. Further data processing and statistical analysis was performed by uploading this .txt file into Perseus (version 1.5.0.15), provided as part of the MaxQuant software solution package (www.maxquant.org). Here, the data were filtered to remove all protein contaminants, reverse-phase proteins, and those proteins only identified by site – an automated data processing feature of the Perseus software. The software was then used for imputation, normalization, PCA, hierarchical clustering and statistical analysis of the data. Briefly, data for analysis was transformed to a log2 scale and missing values were imputed with constant values to allow the assignment of the presence or absence of proteins between conditions. All statistical t-tests, to distinguish proteins differentially expressed between conditions, were performed with a p value threshold of 0.05. For hierarchical clustering, Euclidean distances were applied using logarithmised intensities after z-score normalisation of the statistically significant data. Outputs from Andromeda processing of the raw LC-MS/MS data gave information on the label-free quantification (LFQ) intensity of all measured peptides. One-way ANOVA analysis of the peptide dataset provided a critical value of 1.82 indicating that a fold change in LFQ intensity greater than 1.82 or less than-1.82 represented a statistically significant change in expression. Principal component analysis was undertaken on logarithmised values only. Differentially expressed proteins were further analysed using Ingenuity Pathway Analysis Knowledge Database (Ingenuity Systems) to map statistically significant proteins to the pathways and biological processes in which they were enriched.

### Bioinformatic analysis

Selected proteins of interest were further characterized using annotation or prediction results from Uniprot, PANTHER, exosome databases, SignalIP and Phobius. To prioritise which AS proteins should be further validaded, all 110 proteins within the AS panel were scored between 1 and 5 based on their previous association as an AS biomarker in our previous studies (+1), whether they have previous association as a biomarker or drug target (based on IPA analysis) (+1), if they are classified as a secreted protein (+1) and if they were identified in the Oncomine databases (+1). Proteins with a score ≥2 (n=51) were validated using the SurvExpress bioinformatics platform. Similarly, Hx proteins were scored between 1 and 5 based on whether they matched with hypoxia biomarkers previously reported in the literature (+1), if they have previous association as a biomarker/drug target (+1), if they are classified as ‘secreted’ based on SignalIP and Phobius analysis (+1) and if they were identified in the Oncomine databases (+1) (Table 5). Proteins with a score ≥2 (n=26) were validated using the SurvExpress bioinformatics platform.

### MRM design and data analysis

MRM assay design was performed using Skyline software (MacCoss laboratory, Washington DC version 3.5). Raw LC-MS/MS data from Q-Exactive analysis of the experimental lystates was used to generate spectral librraies in Skyline. Proteotypic peptides with associated spectral library data were selected for all proteins of interest according to the following criteria: no missed cleavages or ‘ragged ends’, sequence length between 7-25 amino acids. Peptide sequences with reactive (C) or methionine (M) residues were avoided. Where possible, peptides that were identified in the discovery (nLC-MS/MS) analysis were prioritized. SRM atlas – which acts as a public repository of developed MRM assays – was also used to guide selection of proteotypic peptides. Between 2 and 3 peptides were selected for each protein initially with 4 - 5 transitions selected per peptide. For all transitions, precursor ions with a charge state of 2 were selected with product ions limited to singly charged y ions. In order to minimize potential interferences, ions with m/z close to the precursor ions or an m/z >1,000 were excluded.

## SUPPLEMENTARY MATERIALS TABLES




