# Supplementary material for: Probing the prostate tumour microenvironment II: Impact of hypoxia on a cell model of prostate cancer progression
Source: Oncotarget. 2017 Jan 10;8(9):15307–37. doi: 10.18632/oncotarget.14574 (PMC5362488; doi:10.18632/oncotarget.14574)
Supplement: Supplementary file 1 [file oncotarget-08-15307-s001.pdf]

Probing the prostate tumour microenvironment II: Impact of hypoxia on a cell model of prostate cancer progression

SUPPLEMENTARY TABLES

Supplementary Table 1: Significantly Changing Proteins as Result of Hypoxic Conditions

See Supplementary File 1

Supplementary Table 2: Common Significantly Changed Proteins Between Androgen Sensitive and Androgen Independent Cell Lines at 8 Hour and 24 Hour Time Points

See Supplementary File 1

Supplementary Table 3: MRM Transition List for Hx Protein Panel

See Supplementary File 1

Supplementary Table 4: MRM Transition List for AS Protein Panel

See Supplementary File 1
