# Supplementary material for: Probing the prostate tumour microenvironment II: Impact of hypoxia on a cell model of prostate cancer progression
Source: Oncotarget. 2017 Jan 10;8(9):15307–37. doi: 10.18632/oncotarget.14574 (PMC5362488; doi:10.18632/oncotarget.14574)
Supplement: Supplementary file 2 [file oncotarget-08-15307-s002.docx]

Table S.1. Significantly Changing Proteins as Result of Hypoxic Conditions

| 8h | | | 24h | | |
| --- | --- | --- | --- | --- | --- |
| Accession Number | Protein IDs | t-test difference | Accession Number | Protein IDs | t-test Difference |
| LNCaP | | | | | |
| O43708 | MAAI_HUMAN | -1.1596 | O00487 | PSDE_HUMAN | -1.3800 |
| O60885 | BRD4_HUMAN | -1.0829 | O15145 | ARPC3_HUMAN | -1.6893 |
| P02144 | MYG_HUMAN | -1.4941 | O94855 | SC24D_HUMAN | 0.3127 |
| Q00325-2 | MPCP_HUMAN | -1.4984 | P08621-2 | RU17_HUMAN | -1.7169 |
| Q06210-2 | GFPT1_HUMAN | -0.5322 | P13987 | CD59_HUMAN | 1.5543 |
| Q5T653 | RM02_HUMAN | -2.1827 | P55884 | EIF3B_HUMAN | -2.0355 |
| Q9BV57 | MTND_HUMAN | -2.2645 | P78344 | IF4G2_HUMAN | 0.9512 |
| Q9UHE8 | STEA1_HUMAN | 2.0228 | P82932 | RT06_HUMAN | -1.1163 |
|  |  |  | Q01130 | SRSF2_HUMAN | -1.4791 |
|  |  |  | Q14671-4 | PUM1_HUMAN | 1.5892 |
|  |  |  | Q7KZF4 | SND1_HUMAN | 1.3984 |
|  |  |  | Q8NFV4 | ABHDB_HUMAN | -0.9861 |
|  |  |  | Q9BSC4 | NOL10_HUMAN | 1.2431 |
|  |  |  | Q9HCU5 | PREB_HUMAN | -0.8344 |
|  |  |  | Q9P013 | CWC15_HUMAN | -2.1234 |
| Abl | | | | | |
| P05114 | HMGN1_HUMAN | -1.0686 | A0AVT1 | UBA6_HUMAN | -1.1821 |
| P07437 | TBB5_HUMAN | -0.2283 | O00468-6 | AGRIN_HUMAN | 1.0992 |
| P43304 | GPDM_HUMAN | 0.2018 | O14786 | NRP1_HUMAN | -1.1205 |
| P46778 | RL21_HUMAN | 1.4371 | O43291 | SPIT2_HUMAN | 1.6977 |
| P49773 | HINT1_HUMAN | -0.8583 | O43678 | NDUA2_HUMAN | -0.9270 |
| P51148 | RAB5C_HUMAN | 1.2509 | O43819 | SCO2_HUMAN | 1.2227 |
| P61011 | SRP54_HUMAN | -0.5123 | O75223 | GGCT_HUMAN | -0.3849 |
| P62081 | RS7_HUMAN | -1.5242 | O75334-5 | LIPA2_HUMAN | -1.3731 |
| P62633-2 | CNBP_HUMAN | -0.9176 | O75475 | PSIP1_HUMAN | -1.3592 |
| P68366 | TBA4A_HUMAN | -1.4386 | O75934 | SPF27_HUMAN | 0.9124 |
| Q00577 | PURA_HUMAN | -1.4632 | O95456 | PSMG1_HUMAN | -0.9065 |
| Q16186 | ADRM1_HUMAN | -1.6658 | P04075 | ALDOA_HUMAN | -1.5997 |
| Q16222-3 | UAP1_HUMAN | 0.2808 | P04406 | G3P_HUMAN | -1.0329 |
| Q6UXN9 | WDR82_HUMAN | -1.1932 | P09497-2 | CLCB_HUMAN | -0.5866 |
| Q7L014 | DDX46_HUMAN | -0.9321 | P10644 | KAP0_HUMAN | -0.9637 |
| Q9NNW7-2 | TRXR2_HUMAN | -0.9381 | P12277 | KCRB_HUMAN | -0.9909 |
| Q9NXV6 | CARF_HUMAN | -1.3580 | P13807-2 | GYS1_HUMAN | -0.6850 |
|  |  |  | P26373 | RL13_HUMAN | 1.2231 |
|  |  |  | P29144 | TPP2_HUMAN | -1.1574 |
|  |  |  | P30405 | PPIF_HUMAN | 0.8756 |
|  |  |  | P48634 | PRC2A_HUMAN | -1.0722 |
|  |  |  | P54886-2 | P5CS_HUMAN | 1.4487 |
|  |  |  | P55209-2 | NP1L1_HUMAN | -1.0053 |
|  |  |  | P56381 | AT5EL_HUMAN;ATP5E_HUMAN | 1.0585 |
|  |  |  | P78347-2 | GTF2I_HUMAN | -1.2137 |
|  |  |  | P84077 | ARF1_HUMAN;ARF3_HUMAN | 0.8212 |
|  |  |  | P84101-4 | SERF2_HUMAN | 1.1101 |
|  |  |  | Q02543 | RL18A_HUMAN | 0.7276 |
|  |  |  | Q02809 | PLOD1_HUMAN | -1.8338 |
|  |  |  | Q07866-8 | KLC1_HUMAN | -1.6313 |
|  |  |  | Q12792 | TWF1_HUMAN | -0.9394 |
|  |  |  | Q13177 | PAK2_HUMAN | -1.4767 |
|  |  |  | Q13363-2 | CTBP1_HUMAN | -1.9206 |
|  |  |  | Q14919 | NC2A_HUMAN | 1.4221 |
|  |  |  | Q15070-2 | OXA1L_HUMAN | 0.7187 |
|  |  |  | Q16774 | KGUA_HUMAN | 1.3241 |
|  |  |  | Q16864 | VATF_HUMAN | -1.4847 |
|  |  |  | Q3LXA3 | DHAK_HUMAN | -1.2630 |
|  |  |  | Q5VW32 | BROX_HUMAN | -0.6041 |
|  |  |  | Q7Z7F7 | RM55_HUMAN | 1.3234 |
|  |  |  | Q86YH6 | DLP1_HUMAN | 1.3702 |
|  |  |  | Q8IZ81 | ELMD2_HUMAN | 0.8822 |
|  |  |  | Q8TCS8 | PNPT1_HUMAN | 0.6600 |
|  |  |  | Q8TD19 | NEK9_HUMAN | -0.4515 |
|  |  |  | Q8WWY3 | PRP31_HUMAN | 0.8160 |
|  |  |  | Q92597 | NDRG1_HUMAN | -1.8854 |
|  |  |  | Q96GW9 | SYMM_HUMAN | 1.2655 |
|  |  |  | Q96JB2 | COG3_HUMAN | -1.0218 |
|  |  |  | Q9BW27 | NUP85_HUMAN | 1.2829 |
|  |  |  | Q9H488 | OFUT1_HUMAN | -1.5014 |
|  |  |  | Q9H936 | GHC1_HUMAN | 1.5645 |
|  |  |  | Q9NWU5 | RM22_HUMAN | -1.5492 |
|  |  |  | Q9NYH9 | UTP6_HUMAN | 1.6388 |
|  |  |  | Q9UL25 | RAB21_HUMAN | 0.5874 |
|  |  |  | Q9Y2R5 | RT17_HUMAN | 1.2581 |
| Hof | | | | | |
| O43390 | HNRPR_HUMAN | 1.2648 | O00159-3 | MYO1C_HUMAN | 0.7150 |
| O75083 | WDR1_HUMAN | -1.3052 | O00629 | IMA3_HUMAN | -1.5896 |
| P14174 | MIF_HUMAN | -1.0917 | O15371 | EIF3D_HUMAN | -1.0116 |
| P14314-2 | GLU2B_HUMAN | -1.4815 | O43707 | ACTN4_HUMAN | -1.3744 |
| P20700 | LMNB1_HUMAN | -0.5330 | O60220 | TIM8A_HUMAN | 1.8624 |
| P22307 | NLTP_HUMAN | -1.1331 | O75347 | TBCA_HUMAN | -1.0090 |
| P25398 | RS12_HUMAN | -1.0857 | O75439 | MPPB_HUMAN | 1.6371 |
| P26373 | RL13_HUMAN | -0.9118 | O75880 | SCO1_HUMAN | 1.3563 |
| P29144 | TPP2_HUMAN | 0.6320 | O95140 | MFN2_HUMAN | 1.4799 |
| P30050 | RL12_HUMAN | 0.3837 | O95168 | NDUB4_HUMAN | 0.9593 |
| P35573 | GDE_HUMAN | -0.4878 | P05386 | RLA1_HUMAN | 1.8656 |
| P49368 | TCPG_HUMAN | -0.6209 | P08621-2 | RU17_HUMAN | -1.6029 |
| P60953 | CDC42_HUMAN | 0.5369 | P09110 | THIK_HUMAN | 0.6912 |
| P62306 | RUXF_HUMAN | -1.0677 | P13861 | KAP2_HUMAN | -1.7412 |
| Q03252 | LMNB2_HUMAN | -0.8522 | P22061 | PIMT_HUMAN | 1.3678 |
| Q10471 | GALT2_HUMAN | 0.6037 | P26358 | DNMT1_HUMAN | 1.3989 |
| Q13277-2 | STX3_HUMAN | -1.0614 | P46063 | RECQ1_HUMAN | -1.5811 |
| Q15233 | NONO_HUMAN | -1.3033 | P47985 | UCRI_HUMAN | 1.0157 |
| Q15369 | ELOC_HUMAN | -1.5651 | P50748 | KNTC1_HUMAN | 1.2178 |
| Q7L5N1 | CSN6_HUMAN | -1.7100 | P61158 | ARP3_HUMAN | 1.2788 |
| Q7Z739 | YTHD3_HUMAN | -0.9447 | P61586 | RHOA_HUMAN | 0.7483 |
| Q7Z7H5 | TMED4_HUMAN | 0.5958 | P62269 | RS18_HUMAN | -0.7255 |
| Q92783-2 | STAM1_HUMAN | -0.7450 | P82909 | RT36_HUMAN | 1.7315 |
| Q969H8 | CS010_HUMAN | -1.0188 | Q01130 | SRSF2_HUMAN | -2.1522 |
| Q969N2-5 | PIGT_HUMAN | 1.3168 | Q07955 | SRSF1_HUMAN | -0.5411 |
| Q96N67-4 | DOCK7_HUMAN | -0.8407 | Q12797 | ASPH_HUMAN | -0.2779 |
| Q9NUU7 | DD19A_HUMAN | -1.2216 | Q12981 | SEC20_HUMAN | 0.7647 |
| Q9UHK6 | AMACR_HUMAN | -0.1843 | Q14203-3 | DCTN1_HUMAN | 1.0632 |
| Q9Y2Q3 | GSTK1_HUMAN | -1.0032 | Q14573 | ITPR3_HUMAN | -0.9659 |
| Q9Y3I0 | RTCB_HUMAN | -0.9857 | Q14CX7 | NAA25_HUMAN | 0.9847 |
| Q9Y3U8 | RL36_HUMAN | -0.7273 | Q15813 | TBCE_HUMAN | 1.2934 |
|  |  |  | Q16718 | NDUA5_HUMAN | 1.3646 |
|  |  |  | Q53H96 | P5CR3_HUMAN | -0.4881 |
|  |  |  | Q5BKZ1 | ZN326_HUMAN | 1.3877 |
|  |  |  | Q6P587 | FAHD1_HUMAN | 0.9942 |
|  |  |  | Q6UB35 | C1TM_HUMAN | 0.6352 |
|  |  |  | Q8N4T8 | CBR4_HUMAN | 1.7041 |
|  |  |  | Q96DG6 | CMBL_HUMAN | -0.7066 |
|  |  |  | Q96S52-2 | PIGS_HUMAN | 1.0535 |
|  |  |  | Q9BSJ8 | ESYT1_HUMAN | 1.1066 |
|  |  |  | Q9BWD1 | THIC_HUMAN | 1.6329 |
|  |  |  | Q9BZE1 | RM37_HUMAN | 1.5714 |
|  |  |  | Q9H2W6 | RM46_HUMAN | 2.0087 |
|  |  |  | Q9NP58-4 | ABCB6_HUMAN | 0.5533 |
|  |  |  | Q9NUQ7 | UFSP2_HUMAN | 1.5797 |
|  |  |  | Q9NVI7-2 | ATD3A_HUMAN | 0.4907 |
|  |  |  | Q9UKM9-2 | RALY_HUMAN | -1.4004 |
|  |  |  | Q9UNM6 | PSD13_HUMAN | -1.6654 |
|  |  |  | Q9Y2Z0-2 | SUGT1_HUMAN | 2.1688 |
|  |  |  | Q9Y4K1 | AIM1_HUMAN | 0.8207 |

Table S.2. Common Significantly Changed Proteins Between Androgen Sensitive and Androgen Independent Cell Lines at 8 Hour and 24 Hour Time Points

|  |  | 8 Hour | | 24 hour | | %CV | |
| --- | --- | --- | --- | --- | --- | --- | --- |
| Accession Number | Protein IDs | (-)Log ANOVA p value | Intensity | (-) Log ANOVA p value | Intensity | SR | TR |
| O00116 | ADAS_HUMAN | 4.44337 | 5724800000 | 5.17244 | 5298500000 | 19.6 | 2.8 |
| O00410 | IPO5_HUMAN | 2.25843 | 19165000000 | 2.45798 | 12778000000 | 11.9 | 3.8 |
| O00429-3 | DNM1L_HUMAN | 2.0122 | 4497100000 | 1.72545 | 3826400000 | 10.4 | 3.5 |
| O00461 | GOLI4_HUMAN | 6.67656 | 7890400000 | 5.02128 | 6620700000 | 24.0 | 3.5 |
| O14776-2 | TCRG1_HUMAN | 1.58076 | 8419000000 | 1.40425 | 4635700000 | 14.7 | 17.6 |
| O15020-2 | SPTN2_HUMAN | 4.21565 | 7183600000 | 4.49254 | 6054300000 | 6.1 | 7.7 |
| O15042-2 | SR140_HUMAN | 2.47231 | 9483600000 | 2.28198 | 6141500000 | 8.1 | 3.5 |
| O15381 | NVL_HUMAN | 1.39302 | 3105500000 | 3.15715 | 4838800000 | 9.3 | 14.3 |
| O15394 | NCAM2_HUMAN | 1.95859 | 7545700000 | 2.34792 | 4283700000 | 13.2 | 12.9 |
| O15439 | MRP4_HUMAN | 3.75011 | 3156100000 | 5.17954 | 3274800000 | 10.8 | 17.6 |
| O43175 | SERA_HUMAN | 1.7659 | 2.0531E+11 | 2.69436 | 1.468E+11 | 4.9 | 6.7 |
| O43252 | PAPS1_HUMAN | 1.55803 | 5610600000 | 1.45936 | 3304600000 | 30.1 | 8.5 |
| O43399 | TPD54_HUMAN | 1.72536 | 11945000000 | 3.59949 | 10411000000 | 19.8 | 11.2 |
| O60313 | OPA1_HUMAN | 4.29174 | 16048000000 | 3.56662 | 13809000000 | 4.0 | 4.3 |
| O75521-2 | ECI2_HUMAN | 1.52473 | 18122000000 | 2.51804 | 13764000000 | 9.6 | 6.2 |
| O75795 | UDB17_HUMAN | 5.2901 | 61593000000 | 4.76501 | 52337000000 | 8.9 | 7.5 |
| O75955 | FLOT1_HUMAN | 5.29135 | 6463000000 | 5.13249 | 9162600000 | 15.6 | 5.6 |
| O94855 | SC24D_HUMAN | 2.59077 | 3678100000 | 5.73377 | 2177500000 | 25.2 | 21.3 |
| O94874 | UFL1_HUMAN | 2.29343 | 18376000000 | 3.98756 | 15858000000 | 5.1 | 3.7 |
| O94905 | ERLN2_HUMAN | 4.22168 | 15871000000 | 1.79437 | 11833000000 | 16.1 | 3.4 |
| O94973 | AP2A2_HUMAN | 2.76918 | 4265200000 | 2.71793 | 4636200000 | 19.5 | 13.5 |
| O95394 | AGM1_HUMAN | 2.31811 | 5429200000 | 3.28053 | 4939000000 | 10.4 | 4.7 |
| O95433 | AHSA1_HUMAN | 1.41252 | 10849000000 | 1.48209 | 9886100000 | 20.4 | 4.5 |
| O95573 | ACSL3_HUMAN | 3.17305 | 11076000000 | 6.31296 | 8992700000 | 5.9 | 2.0 |
| P00338 | LDHA_HUMAN | 4.13488 | 1.6186E+11 | 1.9241 | 1.632E+11 | 11.1 | 2.6 |
| P00441 | SODC_HUMAN | 1.76326 | 57227000000 | 5.00447 | 38850000000 | 4.9 | 6.9 |
| P00491 | PNPH_HUMAN | 2.93935 | 14692000000 | 1.47935 | 11787000000 | 4.4 | 6.6 |
| P04040 | CATA_HUMAN | 2.44642 | 36147000000 | 3.54501 | 27552000000 | 0.2 | 3.4 |
| P04792 | HSPB1_HUMAN | 2.21951 | 1.9046E+11 | 3.53421 | 1.4482E+11 | 4.1 | 3.7 |
| P04844 | RPN2_HUMAN | 2.38298 | 30221000000 | 2.23918 | 26033000000 | 9.0 | 2.8 |
| P06493 | CDK1_HUMAN | 2.11724 | 20124000000 | 1.96733 | 18998000000 | 12.2 | 3.2 |
| P06733 | ENOA_HUMAN | 1.99292 | 3.606E+11 | 1.85062 | 3.2841E+11 | 15.0 | 4.0 |
| P07099 | HYEP_HUMAN | 2.80076 | 2.0545E+11 | 3.23522 | 1.4694E+11 | 27.1 | 3.9 |
| P07195 | LDHB_HUMAN | 6.53083 | 35287000000 | 7.18006 | 31464000000 | 6.6 | 3.3 |
| P07237 | PDIA1_HUMAN | 2.89562 | 2.7349E+11 | 3.38331 | 2.1681E+11 | 9.1 | 3.9 |
| P07339 | CATD_HUMAN | 3.50701 | 40433000000 | 4.04765 | 26154000000 | 21.3 | 8.3 |
| P07686 | HEXB_HUMAN | 3.87015 | 11729000000 | 4.43869 | 8772900000 | 2.9 | 5.8 |
| P08133 | ANXA6_HUMAN | 3.86236 | 19602000000 | 7.61145 | 19367000000 | 6.8 | 6.8 |
| P08195-2 | 4F2_HUMAN | 2.68565 | 46440000000 | 4.84528 | 34765000000 | 6.3 | 4.5 |
| P08240 | SRPR_HUMAN | 4.77171 | 8591100000 | 2.99805 | 7499700000 | 9.7 | 4.5 |
| P08473 | NEP_HUMAN | 2.69914 | 49100000000 | 2.98532 | 42164000000 | 19.5 | 1.1 |
| P09874 | PARP1_HUMAN | 2.94692 | 1.152E+11 | 1.49971 | 89041000000 | 9.3 | 2.0 |
| P10599 | THIO_HUMAN | 1.8418 | 50318000000 | 1.99604 | 52329000000 | 26.4 | 8.8 |
| P11413 | G6PD_HUMAN | 2.58083 | 9650400000 | 1.9866 | 7707400000 | 11.7 | 8.6 |
| P12268 | IMDH2_HUMAN | 2.92761 | 54045000000 | 2.88949 | 44870000000 | 5.0 | 6.9 |
| P12814 | ACTN1_HUMAN | 1.56661 | 9612100000 | 2.41613 | 8164900000 | 6.2 | 6.0 |
| P12956 | XRCC6_HUMAN | 1.71226 | 1.0909E+11 | 3.54335 | 82392000000 | 4.6 | 2.6 |
| P13010 | XRCC5_HUMAN | 2.14636 | 89043000000 | 3.94579 | 70857000000 | 4.4 | 2.6 |
| P13804 | ETFA_HUMAN | 2.13258 | 79217000000 | 1.97939 | 53860000000 | 15.5 | 3.9 |
| P14324-2 | FPPS_HUMAN | 2.54062 | 11294000000 | 3.27858 | 8153400000 | 18.4 | 12.7 |
| P14625 | ENPL_HUMAN | 4.32175 | 3.026E+11 | 3.64344 | 2.7348E+11 | 2.8 | 3.1 |
| P16219 | ACADS_HUMAN | 2.26342 | 4558300000 | 2.15597 | 3574300000 | 13.3 | 8.4 |
| P16401 | H15_HUMAN | 3.33441 | 85557000000 | 3.68927 | 60619000000 | 19.2 | 3.1 |
| P17096 | HMGA1_HUMAN | 2.43295 | 5365100000 | 7.21589 | 4793300000 | 22.2 | 2.5 |
| P20020-6 | AT2B1_HUMAN | 2.08581 | 11249000000 | 1.66837 | 8708900000 | 6.6 | 7.6 |
| P20700 | LMNB1_HUMAN | 1.49792 | 1.2153E+11 | 1.35227 | 92938000000 | 13.0 | 0.2 |
| P21333-2 | FLNA_HUMAN | 3.67707 | 46778000000 | 5.20056 | 40883000000 | 9.2 | 3.3 |
| P21397 | AOFA_HUMAN | 1.50228 | 1.0294E+11 | 1.79326 | 77751000000 | 9.3 | 2.2 |
| P22392-2 | NDKB_HUMAN | 2.65768 | 1.4597E+11 | 4.67999 | 1.2379E+11 | 3.6 | 8.4 |
| P22830 | HEMH_HUMAN | 2.74219 | 20546000000 | 2.88345 | 13970000000 | 9.7 | 5.4 |
| P23526 | SAHH_HUMAN | 3.01231 | 55768000000 | 2.48198 | 40594000000 | 9.5 | 1.8 |
| P25685 | DNJB1_HUMAN | 1.78142 | 3004900000 | 2.51756 | 2904400000 | 20.8 | 3.8 |
| P25787 | PSA2_HUMAN | 1.35787 | 7319100000 | 1.44987 | 7699000000 | 19.4 | 6.3 |
| P26583 | HMGB2_HUMAN | 1.66628 | 30190000000 | 1.92941 | 22760000000 | 17.4 | 6.1 |
| P27797 | CALR_HUMAN | 1.95008 | 1.1022E+11 | 6.09588 | 87587000000 | 0.5 | 1.1 |
| P28290-2 | SSFA2_HUMAN | 5.21184 | 2331700000 | 4.71178 | 2577200000 | 15.7 | 13.9 |
| P29401 | TKT_HUMAN | 2.27906 | 86441000000 | 3.54658 | 64093000000 | 19.1 | 1.2 |
| P29992 | GNA11_HUMAN | 1.60383 | 1813300000 | 2.90479 | 1827900000 | 14.6 | 6.0 |
| P30041 | PRDX6_HUMAN | 2.46555 | 76341000000 | 1.44667 | 63320000000 | 23.6 | 9.5 |
| P30043 | BLVRB_HUMAN | 2.00367 | 8948100000 | 2.04323 | 6654300000 | 7.8 | 0.6 |
| P30101 | PDIA3_HUMAN | 2.04777 | 2.3903E+11 | 3.95535 | 1.8354E+11 | 4.7 | 1.8 |
| P30533 | AMRP_HUMAN | 2.62402 | 8303900000 | 3.46523 | 7835000000 | 18.2 | 8.5 |
| P30566 | PUR8_HUMAN | 2.23471 | 2980600000 | 2.36444 | 1859000000 | 16.0 | 3.6 |
| P30837 | AL1B1_HUMAN | 1.98648 | 11220000000 | 4.43626 | 6691500000 | 6.7 | 12.3 |
| P31689 | DNJA1_HUMAN | 1.64741 | 11707000000 | 1.41814 | 10850000000 | 8.9 | 10.2 |
| P31930 | QCR1_HUMAN | 2.64809 | 52323000000 | 2.70332 | 42192000000 | 3.6 | 2.0 |
| P33121 | ACSL1_HUMAN | 3.69433 | 30222000000 | 4.26265 | 24394000000 | 7.2 | 9.8 |
| P35237 | SPB6_HUMAN | 1.56938 | 10683000000 | 2.0095 | 7914900000 | 14.4 | 8.1 |
| P35573 | GDE_HUMAN | 4.46081 | 5183400000 | 5.58146 | 4476500000 | 13.1 | 11.3 |
| P35580 | MYH10_HUMAN | 2.43197 | 17132000000 | 2.9283 | 13735000000 | 4.7 | 3.8 |
| P38117 | ETFB_HUMAN | 3.45558 | 66067000000 | 6.01143 | 45869000000 | 10.0 | 1.8 |
| P41252 | SYIC_HUMAN | 1.97224 | 24201000000 | 2.75036 | 20555000000 | 3.7 | 7.2 |
| P42704 | LPPRC_HUMAN | 1.82992 | 85178000000 | 2.54099 | 71104000000 | 4.0 | 2.2 |
| P43304 | GPDM_HUMAN | 10.0708 | 15525000000 | 9.27342 | 12971000000 | 1.1 | 0.7 |
| P45954 | ACDSB_HUMAN | 2.12171 | 18508000000 | 1.35193 | 16901000000 | 9.8 | 5.2 |
| P46109 | CRKL_HUMAN | 2.08177 | 1826500000 | 1.39236 | 1985500000 | 6.0 | 18.8 |
| P46939 | UTRO_HUMAN | 7.86849 | 14108000000 | 4.51207 | 11201000000 | 7.0 | 9.1 |
| P46940 | IQGA1_HUMAN | 2.30875 | 27557000000 | 1.90452 | 24238000000 | 2.2 | 4.2 |
| P46977 | STT3A_HUMAN | 1.61207 | 11528000000 | 2.04732 | 8012000000 | 5.7 | 8.0 |
| P48163 | MAOX_HUMAN | 3.14144 | 5653300000 | 2.83902 | 5382000000 | 13.7 | 7.5 |
| P48637 | GSHB_HUMAN | 1.42081 | 12189000000 | 3.09315 | 9098400000 | 7.9 | 1.2 |
| P48735 | IDHP_HUMAN | 3.53741 | 1.0243E+11 | 2.39285 | 89450000000 | 2.5 | 9.9 |
| P49321 | NASP_HUMAN | 2.95282 | 11810000000 | 3.46637 | 8747100000 | 14.7 | 6.2 |
| P49773 | HINT1_HUMAN | 1.60309 | 23120000000 | 1.43145 | 17951000000 | 29.7 | 30.9 |
| P49915 | GUAA_HUMAN | 2.36113 | 22128000000 | 3.85575 | 20563000000 | 11.7 | 5.3 |
| P50851-2 | LRBA_HUMAN | 3.31391 | 3025700000 | 2.25062 | 1766000000 | 24.3 | 19.8 |
| P50995-2 | ANX11_HUMAN | 1.49523 | 9507700000 | 2.65673 | 10053000000 | 1.8 | 2.1 |
| P51114 | FXR1_HUMAN | 1.64063 | 3994900000 | 2.63543 | 3856200000 | 8.5 | 23.9 |
| P51572 | BAP31_HUMAN | 4.36476 | 14308000000 | 4.00171 | 11156000000 | 21.7 | 1.0 |
| P51648 | AL3A2_HUMAN | 2.72634 | 13806000000 | 3.5764 | 9225000000 | 12.7 | 2.9 |
| P53618 | COPB_HUMAN | 1.93747 | 25351000000 | 2.66605 | 23533000000 | 9.1 | 0.3 |
| P54920 | SNAA_HUMAN | 2.05565 | 10066000000 | 1.50019 | 7475400000 | 5.1 | 7.4 |
| P55060-3 | XPO2_HUMAN | 3.12938 | 2.532E+11 | 2.3501 | 1.0621E+11 | 6.2 | 8.7 |
| P60891 | PRPS1_HUMAN | 2.19388 | 3262900000 | 3.15464 | 8716900000 | 0.8 | 2.3 |
| P61604 | CH10_HUMAN | 1.76327 | 1.9499E+11 | 3.02393 | 1.6706E+11 | 6.5 | 6.9 |
| P62081 | RS7_HUMAN | 2.52098 | 53713000000 | 1.37241 | 40841000000 | 10.1 | 9.2 |
| P62249 | RS16_HUMAN | 1.78708 | 67371000000 | 2.15879 | 61577000000 | 4.4 | 6.4 |
| P63151 | 2ABA_HUMAN | 2.16841 | 7598400000 | 3.07421 | 6936000000 | 1.1 | 5.9 |
| P63261 | ACTG_HUMAN | 2.00739 | 1.5806E+12 | 2.02672 | 1.1824E+12 | 13.2 | 3.6 |
| P78527 | PRKDC_HUMAN | 2.15938 | 1.4741E+11 | 1.62191 | 1.2762E+11 | 14.7 | 2.3 |
| P80303-2 | NUCB2_HUMAN | 3.80063 | 23923000000 | 4.2082 | 18111000000 | 8.4 | 6.3 |
| Q00796 | DHSO_HUMAN | 2.44843 | 43506000000 | 2.55814 | 35496000000 | 3.9 | 2.0 |
| Q01105-2 | SET_HUMAN | 1.39877 | 45976000000 | 2.9367 | 37362000000 | 12.8 | 9.1 |
| Q01813 | K6PP_HUMAN | 2.00255 | 8040700000 | 2.85866 | 8708300000 | 10.2 | 4.0 |
| Q02218 | ODO1_HUMAN | 2.17672 | 28266000000 | 3.26874 | 21159000000 | 5.4 | 3.4 |
| Q03154 | ACY1_HUMAN | 1.94562 | 2971100000 | 3.6737 | 1897300000 | 16.8 | 9.1 |
| Q05639 | EF1A2_HUMAN | 3.84794 | 28172000000 | 5.90217 | 22521000000 | 18.1 | 16.1 |
| Q05655 | KPCD_HUMAN | 3.58935 | 1860700000 | 4.13162 | 1663500000 | 34.5 | 3.6 |
| Q08752 | PPID_HUMAN | 1.66285 | 3954400000 | 1.44228 | 3016600000 | 14.0 | 5.2 |
| Q08945 | SSRP1_HUMAN | 2.938 | 22596000000 | 2.01676 | 17877000000 | 10.1 | 6.2 |
| Q10471 | GALT2_HUMAN | 2.73287 | 5113500000 | 2.0774 | 3719800000 | 13.0 | 5.2 |
| Q10567-3 | AP1B1_HUMAN | 3.07871 | 30630000000 | 3.60567 | 27446000000 | 2.6 | 3.2 |
| Q12797 | ASPH_HUMAN | 7.71818 | 11926000000 | 7.28025 | 9225200000 | 9.9 | 16.6 |
| Q12907 | LMAN2_HUMAN | 2.18213 | 16501000000 | 1.61214 | 12324000000 | 9.4 | 4.6 |
| Q13126-4 | MTAP_HUMAN | 3.67011 | 3817000000 | 2.56904 | 2997200000 | 18.0 | 19.7 |
| Q13185 | CBX3_HUMAN | 1.6505 | 25922000000 | 1.81501 | 17832000000 | 10.4 | 1.9 |
| Q13228 | SBP1_HUMAN | 5.72583 | 1.1154E+11 | 6.2336 | 92795000000 | 5.9 | 8.0 |
| Q13409-2 | DC1I2_HUMAN | 1.34449 | 3887300000 | 1.58554 | 2512800000 | 11.3 | 24.8 |
| Q13425 | SNTB2_HUMAN | 4.36275 | 5215300000 | 4.03351 | 5751700000 | 11.7 | 8.2 |
| Q13451 | FKBP5_HUMAN | 2.02347 | 4606900000 | 3.95094 | 3417000000 | 12.8 | 9.9 |
| Q13813-3 | SPTN1_HUMAN | 7.06854 | 8302200000 | 5.62206 | 6906500000 | 6.4 | 7.2 |
| Q13823 | NOG2_HUMAN | 1.83534 | 4471300000 | 2.3478 | 2529600000 | 4.5 | 14.2 |
| Q14166 | TTL12_HUMAN | 4.12978 | 13526000000 | 4.20572 | 11222000000 | 13.8 | 4.2 |
| Q14204 | DYHC1_HUMAN | 1.92441 | 62575000000 | 2.5092 | 55433000000 | 3.0 | 1.9 |
| Q14435 | GALT3_HUMAN | 2.11769 | 6336900000 | 2.67276 | 5706000000 | 8.3 | 11.0 |
| Q14554 | PDIA5_HUMAN | 2.72703 | 3717500000 | 1.81628 | 2811600000 | 17.1 | 11.5 |
| Q14739 | LBR_HUMAN | 4.1927 | 6570200000 | 3.5485 | 6041500000 | 11.3 | 1.7 |
| Q14847 | LASP1_HUMAN | 3.94199 | 3263400000 | 5.11394 | 2630400000 | 24.0 | 5.7 |
| Q14938-5 | NFIX_HUMAN | 3.62855 | 6904900000 | 4.76566 | 5069000000 | 12.5 | 4.7 |
| Q15084-3 | PDIA6_HUMAN | 1.98111 | 63458000000 | 2.53403 | 53931000000 | 9.2 | 3.0 |
| Q15139 | KPCD1_HUMAN | 1.50634 | 6484100000 | 1.45934 | 3944100000 | 0.8 | 5.2 |
| Q15437 | SC23B_HUMAN | 1.96297 | 7494800000 | 1.51196 | 3576700000 | 4.4 | 10.2 |
| Q15691 | MARE1_HUMAN | 1.82042 | 12792000000 | 1.79355 | 10828000000 | 8.7 | 8.0 |
| Q16222-3 | UAP1_HUMAN | 7.58119 | 15631000000 | 8.34022 | 12809000000 | 10.0 | 4.3 |
| Q16625-4 | OCLN_HUMAN | 3.36191 | 8271400000 | 4.5436 | 4455100000 | 12.1 | 3.8 |
| Q16762 | THTR_HUMAN | 2.96609 | 4805400000 | 2.30865 | 3516300000 | 11.7 | 4.9 |
| Q16850 | CP51A_HUMAN | 1.47599 | 3989400000 | 2.64276 | 3365000000 | 10.5 | 23.2 |
| Q1KMD3 | HNRL2_HUMAN | 3.56244 | 23086000000 | 2.60829 | 17518000000 | 10.3 | 2.4 |
| Q53H82 | LACB2_HUMAN | 5.37218 | 10917000000 | 6.36019 | 7572100000 | 3.6 | 7.0 |
| Q53SF7-2 | COBL1_HUMAN | 2.1914 | 2935800000 | 2.61477 | 3304600000 | 18.5 | 11.7 |
| Q5VWZ2 | LYPL1_HUMAN | 1.571 | 7140400000 | 2.77593 | 6361700000 | 7.1 | 19.5 |
| Q5VYK3 | ECM29_HUMAN | 1.35107 | 5100800000 | 2.29886 | 5074000000 | 10.4 | 15.4 |
| Q6DD88 | ATLA3_HUMAN | 4.80591 | 19288000000 | 5.72729 | 15255000000 | 7.0 | 5.9 |
| Q6NVY1 | HIBCH_HUMAN | 4.26322 | 9978100000 | 4.33585 | 9041200000 | 5.1 | 2.4 |
| Q6PKG0 | LARP1_HUMAN | 3.47611 | 4284200000 | 3.50843 | 4226000000 | 12.9 | 5.3 |
| Q6UB35 | C1TM_HUMAN | 2.23157 | 2605000000 | 1.99256 | 2210900000 | 2.4 | 10.1 |
| Q7Z2W4 | ZCCHV_HUMAN | 2.42697 | 7430200000 | 3.36678 | 6126300000 | 8.5 | 9.0 |
| Q8IV36-2 | HID1_HUMAN | 2.20863 | 1985100000 | 4.72257 | 1594200000 | 16.1 | 7.4 |
| Q8IWX8 | CHERP_HUMAN | 1.62548 | 5839500000 | 1.72565 | 3863800000 | 27.9 | 5.7 |
| Q8IYB8 | SUV3_HUMAN | 2.12027 | 2829400000 | 1.8574 | 2065000000 | 30.4 | 4.2 |
| Q8N5K1 | CISD2_HUMAN | 2.57464 | 5254800000 | 2.17541 | 5304400000 | 18.4 | 6.4 |
| Q8NBS9 | TXND5_HUMAN | 4.49713 | 10106000000 | 5.77528 | 9315800000 | 6.1 | 13.3 |
| Q8NFT2-3 | STEA2_HUMAN | 1.88551 | 5784500000 | 2.4593 | 3207800000 | 2.1 | 26.7 |
| Q8TC12 | RDH11_HUMAN | 4.74987 | 19736000000 | 3.42707 | 15434000000 | 4.6 | 5.2 |
| Q8TEM1 | PO210_HUMAN | 1.55567 | 18775000000 | 3.30945 | 16757000000 | 6.0 | 2.6 |
| Q8WUY3-4 | PRUN2_HUMAN | 4.22363 | 5787900000 | 4.70754 | 5923500000 | 30.7 | 0.9 |
| Q8WVV9-5 | HNRLL_HUMAN | 2.56695 | 2838000000 | 2.38681 | 2533600000 | 4.4 | 13.5 |
| Q92820 | GGH_HUMAN | 6.69603 | 10226000000 | 7.75497 | 8783600000 | 1.0 | 7.4 |
| Q92979 | NEP1_HUMAN | 2.22665 | 2411400000 | 1.40629 | 2533700000 | 3.2 | 5.5 |
| Q96A26 | F162A_HUMAN | 1.656 | 22920000000 | 1.6511 | 18003000000 | 22.3 | 12.4 |
| Q96BJ3 | AIDA_HUMAN | 5.66374 | 6017600000 | 5.62787 | 4976200000 | 7.6 | 16.8 |
| Q96CP2 | FWCH2_HUMAN | 1.41589 | 1428600000 | 1.94312 | 1097500000 | 11.0 | 12.8 |
| Q96HC4 | PDLI5_HUMAN | 5.53299 | 11517000000 | 6.64015 | 10827000000 | 11.7 | 5.8 |
| Q96HS1 | PGAM5_HUMAN | 1.32852 | 5502500000 | 4.9649 | 4876200000 | 11.9 | 12.3 |
| Q96N67-4 | DOCK7_HUMAN | 1.48749 | 1836600000 | 1.85603 | 1307600000 | 13.3 | 18.8 |
| Q96P16 | RPR1A_HUMAN | 1.55308 | 2158600000 | 2.50125 | 1755400000 | 13.7 | 16.3 |
| Q96P70 | IPO9_HUMAN | 2.00688 | 5572800000 | 2.03037 | 5246700000 | 5.5 | 7.9 |
| Q96RP9-2 | EFGM_HUMAN | 2.8448 | 14756000000 | 4.62655 | 12170000000 | 4.7 | 3.6 |
| Q9BRA2 | TXD17_HUMAN | 1.6436 | 8686800000 | 3.2158 | 6244400000 | 17.1 | 10.3 |
| Q9BTE3-2 | MCMBP_HUMAN | 2.33872 | 2374200000 | 2.71249 | 2123900000 | 20.9 | 21.8 |
| Q9BVK6 | TMED9_HUMAN | 2.03847 | 7874500000 | 3.26885 | 6421500000 | 9.3 | 20.5 |
| Q9BVP2-2 | GNL3_HUMAN | 2.35634 | 15236000000 | 1.47605 | 12337000000 | 5.9 | 7.3 |
| Q9BWD1 | THIC_HUMAN | 1.46722 | 5559300000 | 1.61062 | 4883200000 | 5.0 | 4.9 |
| Q9BY77 | PDIP3_HUMAN | 1.37549 | 4487100000 | 2.26994 | 3992200000 | 10.3 | 3.5 |
| Q9BZE4 | NOG1_HUMAN | 1.94817 | 16421000000 | 1.31802 | 12340000000 | 20.5 | 0.9 |
| Q9BZF1 | OSBL8_HUMAN | 4.70875 | 5893400000 | 5.15239 | 4358200000 | 9.5 | 6.5 |
| Q9H2U1 | DHX36_HUMAN | 4.66344 | 16951000000 | 6.01559 | 13370000000 | 17.2 | 14.3 |
| Q9H2U2 | IPYR2_HUMAN | 4.6534 | 27408000000 | 5.48975 | 22750000000 | 10.0 | 5.2 |
| Q9H993 | CF211_HUMAN | 2.58181 | 3099500000 | 1.69769 | 2754500000 | 14.8 | 9.8 |
| Q9H9S4 | CB39L_HUMAN | 1.38027 | 2138100000 | 1.48584 | 1855500000 | 4.2 | 6.1 |
| Q9HAV7 | GRPE1_HUMAN | 1.88442 | 20258000000 | 1.501 | 17158000000 | 10.6 | 5.0 |
| Q9HCU5 | PREB_HUMAN | 3.31774 | 1984000000 | 2.80003 | 1395000000 | 24.4 | 4.7 |
| Q9NP61 | ARFG3_HUMAN | 4.10987 | 3625300000 | 4.83687 | 1184600000 | 1.6 | 17.5 |
| Q9NT62 | ATG3_HUMAN | 3.1744 | 4458700000 | 3.45769 | 5053000000 | 12.3 | 5.5 |
| Q9NTI5-2 | PDS5B_HUMAN | 1.30698 | 7747600000 | 2.66873 | 6420800000 | 6.4 | 6.7 |
| Q9NTJ5 | SAC1_HUMAN | 3.55948 | 8720200000 | 1.7807 | 7617000000 | 20.6 | 4.8 |
| Q9NUJ1 | ABHDA_HUMAN | 3.4798 | 34209000000 | 3.72472 | 26395000000 | 20.1 | 7.6 |
| Q9NX62 | IMPA3_HUMAN | 2.25324 | 10517000000 | 3.15747 | 3467700000 | 22.1 | 16.6 |
| Q9P258 | RCC2_HUMAN | 2.30334 | 5102800000 | 2.73137 | 4124500000 | 21.7 | 5.8 |
| Q9P2B2 | FPRP_HUMAN | 1.76074 | 4534700000 | 2.58421 | 3469700000 | 13.1 | 10.4 |
| Q9P2E9 | RRBP1_HUMAN | 1.64179 | 28678000000 | 3.92195 | 22057000000 | 8.0 | 17.6 |
| Q9UBP9 | GULP1_HUMAN | 2.03946 | 4253100000 | 2.201 | 3071900000 | 16.5 | 20.4 |
| Q9UEW8 | STK39_HUMAN | 6.27344 | 10244000000 | 5.68979 | 8056600000 | 21.5 | 8.5 |
| Q9UHB6 | LIMA1_HUMAN | 3.21227 | 7686200000 | 4.47751 | 6439100000 | 5.5 | 4.0 |
| Q9UHD8 | SEPT9_HUMAN | 1.45485 | 17344000000 | 1.92023 | 13855000000 | 19.3 | 4.7 |
| Q9UHQ9 | NB5R1_HUMAN | 2.5329 | 5801000000 | 3.66245 | 4298500000 | 30.3 | 4.1 |
| Q9UM54-6 | MYO6_HUMAN | 5.24597 | 61245000000 | 7.00786 | 51707000000 | 6.1 | 1.4 |
| Q9UN86-2 | G3BP2_HUMAN | 2.32265 | 12524000000 | 3.10182 | 6619000000 | 10.9 | 2.7 |
| Q9UNZ2 | NSF1C_HUMAN | 2.17369 | 5960000000 | 1.37583 | 4838000000 | 13.0 | 14.1 |
| Q9UPQ0-10 | LIMC1_HUMAN | 2.80445 | 11291000000 | 4.31279 | 8004800000 | 13.5 | 9.9 |
| Q9Y230 | RUVB2_HUMAN | 1.96655 | 18969000000 | 1.47915 | 17446000000 | 24.4 | 7.6 |
| Q9Y266 | NUDC_HUMAN | 1.66785 | 17834000000 | 1.83861 | 16553000000 | 14.9 | 2.8 |
| Q9Y394-2 | DHRS7_HUMAN | 1.5809 | 39426000000 | 2.53179 | 42044000000 | 13.5 | 2.6 |
| Q9Y3B8-3 | ORN_HUMAN | 2.32338 | 5300300000 | 2.95445 | 4195100000 | 21.4 | 19.7 |
| Q9Y5B9 | SP16H_HUMAN | 2.71016 | 27895000000 | 1.86858 | 22881000000 | 14.2 | 5.1 |
| Q9Y6E2 | BZW2_HUMAN | 2.79603 | 6078000000 | 3.87997 | 4529200000 | 15.8 | 16.1 |

Table S.3. MRM Transition List for Hx Protein Panel

| Compound Group | Compound Name | ISTD? | Precursor Ion | MS1 Res | Product Ion | MS2 Res | Dwell | Fragmentor | Collision Energy | Cell Accelerator Voltage | Polarity |
| --- | --- | --- | --- | --- | --- | --- | --- | --- | --- | --- | --- |
| sp\|P14174\|MIF_HUMAN | IGGAQNR.light | FALSE | 358.20 | Unit | 602.30 | Unit | 10 | 130 | 12.1 | 4 | Positive |
| sp\|P14174\|MIF_HUMAN | IGGAQNR.light | FALSE | 358.20 | Unit | 545.28 | Unit | 10 | 130 | 12.1 | 4 | Positive |
| sp\|P14174\|MIF_HUMAN | IGGAQNR.light | FALSE | 358.20 | Unit | 417.22 | Unit | 10 | 130 | 12.1 | 4 | Positive |
| sp\|P14174\|MIF_HUMAN | IGGAQNR.light | FALSE | 358.20 | Unit | 175.12 | Unit | 10 | 130 | 12.1 | 4 | Positive |
| sp\|P14174\|MIF_HUMAN | LLC[+57.0]GLLAER.light | FALSE | 522.80 | Unit | 818.42 | Unit | 10 | 130 | 17.2 | 4 | Positive |
| sp\|P14174\|MIF_HUMAN | LLC[+57.0]GLLAER.light | FALSE | 522.80 | Unit | 658.39 | Unit | 10 | 130 | 17.2 | 4 | Positive |
| sp\|P14174\|MIF_HUMAN | LLC[+57.0]GLLAER.light | FALSE | 522.80 | Unit | 488.28 | Unit | 10 | 130 | 17.2 | 4 | Positive |
| sp\|P14174\|MIF_HUMAN | LLC[+57.0]GLLAER.light | FALSE | 522.80 | Unit | 375.20 | Unit | 10 | 130 | 17.2 | 4 | Positive |
| sp\|Q969N2\|PIGT_HUMAN | GELSTLLYNTHPYR.light | FALSE | 832.43 | Unit | 1176.62 | Unit | 10 | 130 | 26.8 | 4 | Positive |
| sp\|Q969N2\|PIGT_HUMAN | GELSTLLYNTHPYR.light | FALSE | 832.43 | Unit | 1063.53 | Unit | 10 | 130 | 26.8 | 4 | Positive |
| sp\|Q969N2\|PIGT_HUMAN | GELSTLLYNTHPYR.light | FALSE | 832.43 | Unit | 950.45 | Unit | 10 | 130 | 26.8 | 4 | Positive |
| sp\|Q969N2\|PIGT_HUMAN | GELSTLLYNTHPYR.light | FALSE | 832.43 | Unit | 435.24 | Unit | 10 | 130 | 26.8 | 4 | Positive |
| sp\|Q969N2\|PIGT_HUMAN | LYVHTLTITSK.light | FALSE | 638.37 | Unit | 900.51 | Unit | 10 | 130 | 20.8 | 4 | Positive |
| sp\|Q969N2\|PIGT_HUMAN | LYVHTLTITSK.light | FALSE | 638.37 | Unit | 763.46 | Unit | 10 | 130 | 20.8 | 4 | Positive |
| sp\|Q969N2\|PIGT_HUMAN | LYVHTLTITSK.light | FALSE | 638.37 | Unit | 234.14 | Unit | 10 | 130 | 20.8 | 4 | Positive |
| sp\|Q969N2\|PIGT_HUMAN | LYVHTLTITSK.light | FALSE | 638.37 | Unit | 147.11 | Unit | 10 | 130 | 20.8 | 4 | Positive |
| sp\|P04075\|ALDOA_HUMAN | ALQASALK.light | FALSE | 401.25 | Unit | 617.36 | Unit | 10 | 130 | 13.4 | 4 | Positive |
| sp\|P04075\|ALDOA_HUMAN | ALQASALK.light | FALSE | 401.25 | Unit | 489.30 | Unit | 10 | 130 | 13.4 | 4 | Positive |
| sp\|P04075\|ALDOA_HUMAN | ALQASALK.light | FALSE | 401.25 | Unit | 260.20 | Unit | 10 | 130 | 13.4 | 4 | Positive |
| sp\|P04075\|ALDOA_HUMAN | ALQASALK.light | FALSE | 401.25 | Unit | 147.11 | Unit | 10 | 130 | 13.4 | 4 | Positive |
| sp\|P04075\|ALDOA_HUMAN | AAQEEYVK.light | FALSE | 469.23 | Unit | 795.39 | Unit | 10 | 130 | 15.5 | 4 | Positive |
| sp\|P04075\|ALDOA_HUMAN | AAQEEYVK.light | FALSE | 469.23 | Unit | 667.33 | Unit | 10 | 130 | 15.5 | 4 | Positive |
| sp\|P04075\|ALDOA_HUMAN | AAQEEYVK.light | FALSE | 469.23 | Unit | 246.18 | Unit | 10 | 130 | 15.5 | 4 | Positive |
| sp\|P04075\|ALDOA_HUMAN | AAQEEYVK.light | FALSE | 469.23 | Unit | 147.11 | Unit | 10 | 130 | 15.5 | 4 | Positive |
| sp\|P05114\|HMGN1_HUMAN | VSSAEGAAK.light | FALSE | 410.21 | Unit | 720.35 | Unit | 10 | 130 | 13.7 | 4 | Positive |
| sp\|P05114\|HMGN1_HUMAN | VSSAEGAAK.light | FALSE | 410.21 | Unit | 633.32 | Unit | 10 | 130 | 13.7 | 4 | Positive |
| sp\|P05114\|HMGN1_HUMAN | VSSAEGAAK.light | FALSE | 410.21 | Unit | 346.21 | Unit | 10 | 130 | 13.7 | 4 | Positive |
| sp\|P05114\|HMGN1_HUMAN | VSSAEGAAK.light | FALSE | 410.21 | Unit | 147.11 | Unit | 10 | 130 | 13.7 | 4 | Positive |
| sp\|P05114\|HMGN1_HUMAN | QAEVANQETK.light | FALSE | 559.28 | Unit | 789.41 | Unit | 10 | 130 | 18.3 | 4 | Positive |
| sp\|P05114\|HMGN1_HUMAN | QAEVANQETK.light | FALSE | 559.28 | Unit | 690.34 | Unit | 10 | 130 | 18.3 | 4 | Positive |
| sp\|P05114\|HMGN1_HUMAN | QAEVANQETK.light | FALSE | 559.28 | Unit | 619.30 | Unit | 10 | 130 | 18.3 | 4 | Positive |
| sp\|P05114\|HMGN1_HUMAN | QAEVANQETK.light | FALSE | 559.28 | Unit | 248.16 | Unit | 10 | 130 | 18.3 | 4 | Positive |
| sp\|Q14573\|ITPR3_HUMAN | IADVVLLQK.light | FALSE | 499.82 | Unit | 885.54 | Unit | 10 | 130 | 16.5 | 4 | Positive |
| sp\|Q14573\|ITPR3_HUMAN | IADVVLLQK.light | FALSE | 499.82 | Unit | 814.50 | Unit | 10 | 130 | 16.5 | 4 | Positive |
| sp\|Q14573\|ITPR3_HUMAN | IADVVLLQK.light | FALSE | 499.82 | Unit | 388.26 | Unit | 10 | 130 | 16.5 | 4 | Positive |
| sp\|Q14573\|ITPR3_HUMAN | IADVVLLQK.light | FALSE | 499.82 | Unit | 147.11 | Unit | 10 | 130 | 16.5 | 4 | Positive |
| sp\|Q14573\|ITPR3_HUMAN | LWTEIPTAITIK.light | FALSE | 693.41 | Unit | 1086.64 | Unit | 10 | 130 | 22.5 | 4 | Positive |
| sp\|Q14573\|ITPR3_HUMAN | LWTEIPTAITIK.light | FALSE | 693.41 | Unit | 856.55 | Unit | 10 | 130 | 22.5 | 4 | Positive |
| sp\|Q14573\|ITPR3_HUMAN | LWTEIPTAITIK.light | FALSE | 693.41 | Unit | 743.47 | Unit | 10 | 130 | 22.5 | 4 | Positive |
| sp\|Q14573\|ITPR3_HUMAN | LWTEIPTAITIK.light | FALSE | 693.41 | Unit | 147.11 | Unit | 10 | 130 | 22.5 | 4 | Positive |
| sp\|O00468\|AGRIN_HUMAN | EAAC[+57.0]LQQTQIEEAR.light | FALSE | 823.89 | Unit | 1102.55 | Unit | 10 | 130 | 26.5 | 4 | Positive |
| sp\|O00468\|AGRIN_HUMAN | EAAC[+57.0]LQQTQIEEAR.light | FALSE | 823.89 | Unit | 974.49 | Unit | 10 | 130 | 26.5 | 4 | Positive |
| sp\|O00468\|AGRIN_HUMAN | EAAC[+57.0]LQQTQIEEAR.light | FALSE | 823.89 | Unit | 846.43 | Unit | 10 | 130 | 26.5 | 4 | Positive |
| sp\|O00468\|AGRIN_HUMAN | EAAC[+57.0]LQQTQIEEAR.light | FALSE | 823.89 | Unit | 504.24 | Unit | 10 | 130 | 26.5 | 4 | Positive |
| sp\|O00468\|AGRIN_HUMAN | SAGDVDTLAFDGR.light | FALSE | 662.31 | Unit | 894.43 | Unit | 10 | 130 | 21.5 | 4 | Positive |
| sp\|O00468\|AGRIN_HUMAN | SAGDVDTLAFDGR.light | FALSE | 662.31 | Unit | 779.40 | Unit | 10 | 130 | 21.5 | 4 | Positive |
| sp\|O00468\|AGRIN_HUMAN | SAGDVDTLAFDGR.light | FALSE | 662.31 | Unit | 565.27 | Unit | 10 | 130 | 21.5 | 4 | Positive |
| sp\|O00468\|AGRIN_HUMAN | SAGDVDTLAFDGR.light | FALSE | 662.31 | Unit | 494.24 | Unit | 10 | 130 | 21.5 | 4 | Positive |
| sp\|Q9H488\|OFUT1_HUMAN | LEPLQAYHR.light | FALSE | 563.80 | Unit | 884.47 | Unit | 10 | 130 | 18.5 | 4 | Positive |
| sp\|Q9H488\|OFUT1_HUMAN | LEPLQAYHR.light | FALSE | 563.80 | Unit | 787.42 | Unit | 10 | 130 | 18.5 | 4 | Positive |
| sp\|Q9H488\|OFUT1_HUMAN | LEPLQAYHR.light | FALSE | 563.80 | Unit | 674.34 | Unit | 10 | 130 | 18.5 | 4 | Positive |
| sp\|Q9H488\|OFUT1_HUMAN | LEPLQAYHR.light | FALSE | 563.80 | Unit | 546.28 | Unit | 10 | 130 | 18.5 | 4 | Positive |
| sp\|Q9H488\|OFUT1_HUMAN | DGTAGSHFMASPQC[+57.0]VGYSR.light | FALSE | 1014.44 | Unit | 1124.52 | Unit | 10 | 130 | 32.4 | 4 | Positive |
| sp\|Q9H488\|OFUT1_HUMAN | DGTAGSHFMASPQC[+57.0]VGYSR.light | FALSE | 1014.44 | Unit | 1053.48 | Unit | 10 | 130 | 32.4 | 4 | Positive |
| sp\|Q9H488\|OFUT1_HUMAN | DGTAGSHFMASPQC[+57.0]VGYSR.light | FALSE | 1014.44 | Unit | 966.45 | Unit | 10 | 130 | 32.4 | 4 | Positive |
| sp\|Q9H488\|OFUT1_HUMAN | DGTAGSHFMASPQC[+57.0]VGYSR.light | FALSE | 1014.44 | Unit | 482.24 | Unit | 10 | 130 | 32.4 | 4 | Positive |
| sp\|O43291\|SPIT2_HUMAN | SIHDFC[+57.0]LVSK.light | FALSE | 603.30 | Unit | 147.11 | Unit | 10 | 130 | 19.7 | 4 | Positive |
| sp\|Q02809\|PLOD1_HUMAN | IFQNLDGALDEVVLK.light | FALSE | 837.46 | Unit | 1058.57 | Unit | 10 | 130 | 27 | 4 | Positive |
| sp\|Q02809\|PLOD1_HUMAN | IFQNLDGALDEVVLK.light | FALSE | 837.46 | Unit | 943.55 | Unit | 10 | 130 | 27 | 4 | Positive |
| sp\|Q02809\|PLOD1_HUMAN | IFQNLDGALDEVVLK.light | FALSE | 837.46 | Unit | 260.20 | Unit | 10 | 130 | 27 | 4 | Positive |
| sp\|Q02809\|PLOD1_HUMAN | IFQNLDGALDEVVLK.light | FALSE | 837.46 | Unit | 147.11 | Unit | 10 | 130 | 27 | 4 | Positive |
| sp\|Q02809\|PLOD1_HUMAN | LTHYHEGLPTTR.light | FALSE | 712.87 | Unit | 910.47 | Unit | 10 | 130 | 23.1 | 4 | Positive |
| sp\|Q02809\|PLOD1_HUMAN | LTHYHEGLPTTR.light | FALSE | 712.87 | Unit | 773.42 | Unit | 10 | 130 | 23.1 | 4 | Positive |
| sp\|Q02809\|PLOD1_HUMAN | LTHYHEGLPTTR.light | FALSE | 712.87 | Unit | 644.37 | Unit | 10 | 130 | 23.1 | 4 | Positive |
| sp\|Q02809\|PLOD1_HUMAN | LTHYHEGLPTTR.light | FALSE | 712.87 | Unit | 175.12 | Unit | 10 | 130 | 23.1 | 4 | Positive |
| sp\|Q10471\|GALT2_HUMAN | AAEVWMDEYK.light | FALSE | 621.28 | Unit | 970.43 | Unit | 10 | 130 | 20.3 | 4 | Positive |
| sp\|Q10471\|GALT2_HUMAN | AAEVWMDEYK.light | FALSE | 621.28 | Unit | 871.37 | Unit | 10 | 130 | 20.3 | 4 | Positive |
| sp\|Q10471\|GALT2_HUMAN | AAEVWMDEYK.light | FALSE | 621.28 | Unit | 685.29 | Unit | 10 | 130 | 20.3 | 4 | Positive |
| sp\|Q10471\|GALT2_HUMAN | AAEVWMDEYK.light | FALSE | 621.28 | Unit | 147.11 | Unit | 10 | 130 | 20.3 | 4 | Positive |
| sp\|Q10471\|GALT2_HUMAN | NFYYAAVPSAR.light | FALSE | 629.81 | Unit | 997.51 | Unit | 10 | 130 | 20.5 | 4 | Positive |
| sp\|Q10471\|GALT2_HUMAN | NFYYAAVPSAR.light | FALSE | 629.81 | Unit | 834.45 | Unit | 10 | 130 | 20.5 | 4 | Positive |
| sp\|Q10471\|GALT2_HUMAN | NFYYAAVPSAR.light | FALSE | 629.81 | Unit | 671.38 | Unit | 10 | 130 | 20.5 | 4 | Positive |
| sp\|Q10471\|GALT2_HUMAN | NFYYAAVPSAR.light | FALSE | 629.81 | Unit | 430.24 | Unit | 10 | 130 | 20.5 | 4 | Positive |
| sp\|Q7L5N1\|CSN6_HUMAN | IGVDHVAR.light | FALSE | 433.75 | Unit | 753.40 | Unit | 10 | 130 | 14.4 | 4 | Positive |
| sp\|Q7L5N1\|CSN6_HUMAN | IGVDHVAR.light | FALSE | 433.75 | Unit | 696.38 | Unit | 10 | 130 | 14.4 | 4 | Positive |
| sp\|Q7L5N1\|CSN6_HUMAN | IGVDHVAR.light | FALSE | 433.75 | Unit | 597.31 | Unit | 10 | 130 | 14.4 | 4 | Positive |
| sp\|Q7L5N1\|CSN6_HUMAN | IGVDHVAR.light | FALSE | 433.75 | Unit | 482.28 | Unit | 10 | 130 | 14.4 | 4 | Positive |
| sp\|Q7L5N1\|CSN6_HUMAN | FNVLYDR.light | FALSE | 463.74 | Unit | 665.36 | Unit | 10 | 130 | 15.4 | 4 | Positive |
| sp\|Q7L5N1\|CSN6_HUMAN | FNVLYDR.light | FALSE | 463.74 | Unit | 566.29 | Unit | 10 | 130 | 15.4 | 4 | Positive |
| sp\|Q7L5N1\|CSN6_HUMAN | FNVLYDR.light | FALSE | 463.74 | Unit | 453.21 | Unit | 10 | 130 | 15.4 | 4 | Positive |
| sp\|Q7L5N1\|CSN6_HUMAN | FNVLYDR.light | FALSE | 463.74 | Unit | 175.12 | Unit | 10 | 130 | 15.4 | 4 | Positive |
| sp\|P13987\|CD59_HUMAN | AGLQVYNK.light | FALSE | 446.75 | Unit | 523.29 | Unit | 10 | 130 | 14.8 | 4 | Positive |
| sp\|P13987\|CD59_HUMAN | AGLQVYNK.light | FALSE | 446.75 | Unit | 424.22 | Unit | 10 | 130 | 14.8 | 4 | Positive |
| sp\|P13987\|CD59_HUMAN | AGLQVYNK.light | FALSE | 446.75 | Unit | 261.16 | Unit | 10 | 130 | 14.8 | 4 | Positive |
| sp\|P13987\|CD59_HUMAN | AGLQVYNK.light | FALSE | 446.75 | Unit | 147.11 | Unit | 10 | 130 | 14.8 | 4 | Positive |
| sp\|P13987\|CD59_HUMAN | FEHC[+57.0]NFNDVTTR.light | FALSE | 770.34 | Unit | 1126.49 | Unit | 10 | 130 | 24.9 | 4 | Positive |
| sp\|P13987\|CD59_HUMAN | FEHC[+57.0]NFNDVTTR.light | FALSE | 770.34 | Unit | 966.46 | Unit | 10 | 130 | 24.9 | 4 | Positive |
| sp\|P13987\|CD59_HUMAN | FEHC[+57.0]NFNDVTTR.light | FALSE | 770.34 | Unit | 852.42 | Unit | 10 | 130 | 24.9 | 4 | Positive |
| sp\|P13987\|CD59_HUMAN | FEHC[+57.0]NFNDVTTR.light | FALSE | 770.34 | Unit | 476.28 | Unit | 10 | 130 | 24.9 | 4 | Positive |
| sp\|Q03252\|LMNB2_HUMAN | ALYESELADAR.light | FALSE | 619.31 | Unit | 1053.48 | Unit | 10 | 130 | 20.2 | 4 | Positive |
| sp\|Q03252\|LMNB2_HUMAN | ALYESELADAR.light | FALSE | 619.31 | Unit | 890.42 | Unit | 10 | 130 | 20.2 | 4 | Positive |
| sp\|Q03252\|LMNB2_HUMAN | ALYESELADAR.light | FALSE | 619.31 | Unit | 761.38 | Unit | 10 | 130 | 20.2 | 4 | Positive |
| sp\|Q03252\|LMNB2_HUMAN | ALYESELADAR.light | FALSE | 619.31 | Unit | 246.16 | Unit | 10 | 130 | 20.2 | 4 | Positive |
| sp\|Q03252\|LMNB2_HUMAN | LELEQTYQAK.light | FALSE | 611.82 | Unit | 980.50 | Unit | 10 | 130 | 20 | 4 | Positive |
| sp\|Q03252\|LMNB2_HUMAN | LELEQTYQAK.light | FALSE | 611.82 | Unit | 867.42 | Unit | 10 | 130 | 20 | 4 | Positive |
| sp\|Q03252\|LMNB2_HUMAN | LELEQTYQAK.light | FALSE | 611.82 | Unit | 738.38 | Unit | 10 | 130 | 20 | 4 | Positive |
| sp\|Q03252\|LMNB2_HUMAN | LELEQTYQAK.light | FALSE | 611.82 | Unit | 147.11 | Unit | 10 | 130 | 20 | 4 | Positive |
| sp\|Q5T653\|RM02_HUMAN | VHGIGGGHK.light | FALSE | 431.24 | Unit | 762.40 | Unit | 10 | 130 | 14.4 | 4 | Positive |
| sp\|Q5T653\|RM02_HUMAN | VHGIGGGHK.light | FALSE | 431.24 | Unit | 625.34 | Unit | 10 | 130 | 14.4 | 4 | Positive |
| sp\|Q5T653\|RM02_HUMAN | VHGIGGGHK.light | FALSE | 431.24 | Unit | 455.24 | Unit | 10 | 130 | 14.4 | 4 | Positive |
| sp\|Q5T653\|RM02_HUMAN | VHGIGGGHK.light | FALSE | 431.24 | Unit | 147.11 | Unit | 10 | 130 | 14.4 | 4 | Positive |
| sp\|Q5T653\|RM02_HUMAN | VSNVDHNK.light | FALSE | 456.73 | Unit | 813.38 | Unit | 10 | 130 | 15.2 | 4 | Positive |
| sp\|Q5T653\|RM02_HUMAN | VSNVDHNK.light | FALSE | 456.73 | Unit | 726.35 | Unit | 10 | 130 | 15.2 | 4 | Positive |
| sp\|Q5T653\|RM02_HUMAN | VSNVDHNK.light | FALSE | 456.73 | Unit | 398.21 | Unit | 10 | 130 | 15.2 | 4 | Positive |
| sp\|Q5T653\|RM02_HUMAN | VSNVDHNK.light | FALSE | 456.73 | Unit | 147.11 | Unit | 10 | 130 | 15.2 | 4 | Positive |
| sp\|Q969H8\|MYDGF_HUMAN | SYLYFTQFK.light | FALSE | 598.80 | Unit | 946.50 | Unit | 10 | 130 | 19.6 | 4 | Positive |
| sp\|Q969H8\|MYDGF_HUMAN | SYLYFTQFK.light | FALSE | 598.80 | Unit | 833.42 | Unit | 10 | 130 | 19.6 | 4 | Positive |
| sp\|Q969H8\|MYDGF_HUMAN | SYLYFTQFK.light | FALSE | 598.80 | Unit | 670.36 | Unit | 10 | 130 | 19.6 | 4 | Positive |
| sp\|Q969H8\|MYDGF_HUMAN | SYLYFTQFK.light | FALSE | 598.80 | Unit | 523.29 | Unit | 10 | 130 | 19.6 | 4 | Positive |
| sp\|Q969H8\|MYDGF_HUMAN | GAEIEYAMAYSK.light | FALSE | 666.81 | Unit | 962.43 | Unit | 10 | 130 | 21.7 | 4 | Positive |
| sp\|Q969H8\|MYDGF_HUMAN | GAEIEYAMAYSK.light | FALSE | 666.81 | Unit | 833.39 | Unit | 10 | 130 | 21.7 | 4 | Positive |
| sp\|Q969H8\|MYDGF_HUMAN | GAEIEYAMAYSK.light | FALSE | 666.81 | Unit | 670.32 | Unit | 10 | 130 | 21.7 | 4 | Positive |
| sp\|Q969H8\|MYDGF_HUMAN | GAEIEYAMAYSK.light | FALSE | 666.81 | Unit | 234.14 | Unit | 10 | 130 | 21.7 | 4 | Positive |
| sp\|P43304\|GPDM_HUMAN | TVGLFLQGGK.light | FALSE | 510.30 | Unit | 819.47 | Unit | 10 | 130 | 16.8 | 4 | Positive |
| sp\|P43304\|GPDM_HUMAN | TVGLFLQGGK.light | FALSE | 510.30 | Unit | 649.37 | Unit | 10 | 130 | 16.8 | 4 | Positive |
| sp\|P43304\|GPDM_HUMAN | TVGLFLQGGK.light | FALSE | 510.30 | Unit | 502.30 | Unit | 10 | 130 | 16.8 | 4 | Positive |
| sp\|P43304\|GPDM_HUMAN | TVGLFLQGGK.light | FALSE | 510.30 | Unit | 147.11 | Unit | 10 | 130 | 16.8 | 4 | Positive |
| sp\|P43304\|GPDM_HUMAN | LAFLNVQAAEEALPR.light | FALSE | 821.45 | Unit | 1197.62 | Unit | 10 | 130 | 26.5 | 4 | Positive |
| sp\|P43304\|GPDM_HUMAN | LAFLNVQAAEEALPR.light | FALSE | 821.45 | Unit | 984.51 | Unit | 10 | 130 | 26.5 | 4 | Positive |
| sp\|P43304\|GPDM_HUMAN | LAFLNVQAAEEALPR.light | FALSE | 821.45 | Unit | 856.45 | Unit | 10 | 130 | 26.5 | 4 | Positive |
| sp\|P43304\|GPDM_HUMAN | LAFLNVQAAEEALPR.light | FALSE | 821.45 | Unit | 272.17 | Unit | 10 | 130 | 26.5 | 4 | Positive |
| sp\|P14314\|GLU2B_HUMAN | LIELQAGK.light | FALSE | 436.27 | Unit | 758.44 | Unit | 10 | 130 | 14.5 | 4 | Positive |
| sp\|P14314\|GLU2B_HUMAN | LIELQAGK.light | FALSE | 436.27 | Unit | 645.36 | Unit | 10 | 130 | 14.5 | 4 | Positive |
| sp\|P14314\|GLU2B_HUMAN | LIELQAGK.light | FALSE | 436.27 | Unit | 516.31 | Unit | 10 | 130 | 14.5 | 4 | Positive |
| sp\|P14314\|GLU2B_HUMAN | LIELQAGK.light | FALSE | 436.27 | Unit | 275.17 | Unit | 10 | 130 | 14.5 | 4 | Positive |
| sp\|P14314\|GLU2B_HUMAN | AQQEQELAADAFK.light | FALSE | 724.85 | Unit | 992.50 | Unit | 10 | 130 | 23.5 | 4 | Positive |
| sp\|P14314\|GLU2B_HUMAN | AQQEQELAADAFK.light | FALSE | 724.85 | Unit | 622.32 | Unit | 10 | 130 | 23.5 | 4 | Positive |
| sp\|P14314\|GLU2B_HUMAN | AQQEQELAADAFK.light | FALSE | 724.85 | Unit | 480.25 | Unit | 10 | 130 | 23.5 | 4 | Positive |
| sp\|P14314\|GLU2B_HUMAN | AQQEQELAADAFK.light | FALSE | 724.85 | Unit | 365.22 | Unit | 10 | 130 | 23.5 | 4 | Positive |
| sp\|Q7Z7H5\|TMED4_HUMAN | LTELQLR.light | FALSE | 436.76 | Unit | 759.44 | Unit | 10 | 130 | 14.5 | 4 | Positive |
| sp\|Q7Z7H5\|TMED4_HUMAN | LTELQLR.light | FALSE | 436.76 | Unit | 658.39 | Unit | 10 | 130 | 14.5 | 4 | Positive |
| sp\|Q7Z7H5\|TMED4_HUMAN | LTELQLR.light | FALSE | 436.76 | Unit | 529.35 | Unit | 10 | 130 | 14.5 | 4 | Positive |
| sp\|Q7Z7H5\|TMED4_HUMAN | LTELQLR.light | FALSE | 436.76 | Unit | 175.12 | Unit | 10 | 130 | 14.5 | 4 | Positive |
| sp\|Q7Z7H5\|TMED4_HUMAN | QLLDQVEQIQK.light | FALSE | 671.37 | Unit | 1100.59 | Unit | 10 | 130 | 21.8 | 4 | Positive |
| sp\|Q7Z7H5\|TMED4_HUMAN | QLLDQVEQIQK.light | FALSE | 671.37 | Unit | 987.51 | Unit | 10 | 130 | 21.8 | 4 | Positive |
| sp\|Q7Z7H5\|TMED4_HUMAN | QLLDQVEQIQK.light | FALSE | 671.37 | Unit | 645.36 | Unit | 10 | 130 | 21.8 | 4 | Positive |
| sp\|Q7Z7H5\|TMED4_HUMAN | QLLDQVEQIQK.light | FALSE | 671.37 | Unit | 147.11 | Unit | 10 | 130 | 21.8 | 4 | Positive |
| sp\|A0AVT1\|UBA6_HUMAN | AVTIHDTEK.light | FALSE | 507.27 | Unit | 843.42 | Unit | 10 | 130 | 16.7 | 4 | Positive |
| sp\|A0AVT1\|UBA6_HUMAN | AVTIHDTEK.light | FALSE | 507.27 | Unit | 629.29 | Unit | 10 | 130 | 16.7 | 4 | Positive |
| sp\|A0AVT1\|UBA6_HUMAN | AVTIHDTEK.light | FALSE | 507.27 | Unit | 492.23 | Unit | 10 | 130 | 16.7 | 4 | Positive |
| sp\|A0AVT1\|UBA6_HUMAN | AVTIHDTEK.light | FALSE | 507.27 | Unit | 147.11 | Unit | 10 | 130 | 16.7 | 4 | Positive |
| sp\|A0AVT1\|UBA6_HUMAN | LETGQFLTFR.light | FALSE | 606.32 | Unit | 969.52 | Unit | 10 | 130 | 19.8 | 4 | Positive |
| sp\|A0AVT1\|UBA6_HUMAN | LETGQFLTFR.light | FALSE | 606.32 | Unit | 868.47 | Unit | 10 | 130 | 19.8 | 4 | Positive |
| sp\|A0AVT1\|UBA6_HUMAN | LETGQFLTFR.light | FALSE | 606.32 | Unit | 683.39 | Unit | 10 | 130 | 19.8 | 4 | Positive |
| sp\|A0AVT1\|UBA6_HUMAN | LETGQFLTFR.light | FALSE | 606.32 | Unit | 423.24 | Unit | 10 | 130 | 19.8 | 4 | Positive |
| sp\|Q7KZF4\|SND1_HUMAN | DTPDEPWAFPAR.light | FALSE | 701.33 | Unit | 1185.57 | Unit | 10 | 130 | 22.7 | 4 | Positive |
| sp\|Q7KZF4\|SND1_HUMAN | DTPDEPWAFPAR.light | FALSE | 701.33 | Unit | 844.45 | Unit | 10 | 130 | 22.7 | 4 | Positive |
| sp\|Q7KZF4\|SND1_HUMAN | DTPDEPWAFPAR.light | FALSE | 701.33 | Unit | 561.31 | Unit | 10 | 130 | 22.7 | 4 | Positive |
| sp\|Q7KZF4\|SND1_HUMAN | DTPDEPWAFPAR.light | FALSE | 701.33 | Unit | 343.21 | Unit | 10 | 130 | 22.7 | 4 | Positive |
| sp\|Q7KZF4\|SND1_HUMAN | DYVAPTANLDQK.light | FALSE | 667.83 | Unit | 957.50 | Unit | 10 | 130 | 21.7 | 4 | Positive |
| sp\|Q7KZF4\|SND1_HUMAN | DYVAPTANLDQK.light | FALSE | 667.83 | Unit | 886.46 | Unit | 10 | 130 | 21.7 | 4 | Positive |
| sp\|Q7KZF4\|SND1_HUMAN | DYVAPTANLDQK.light | FALSE | 667.83 | Unit | 688.36 | Unit | 10 | 130 | 21.7 | 4 | Positive |
| sp\|Q7KZF4\|SND1_HUMAN | DYVAPTANLDQK.light | FALSE | 667.83 | Unit | 275.17 | Unit | 10 | 130 | 21.7 | 4 | Positive |
| sp\|Q12792\|TWF1_HUMAN | DYDSFVLPLLEDK.light | FALSE | 777.39 | Unit | 1073.62 | Unit | 10 | 130 | 25.1 | 4 | Positive |
| sp\|Q12792\|TWF1_HUMAN | DYDSFVLPLLEDK.light | FALSE | 777.39 | Unit | 926.56 | Unit | 10 | 130 | 25.1 | 4 | Positive |
| sp\|Q12792\|TWF1_HUMAN | DYDSFVLPLLEDK.light | FALSE | 777.39 | Unit | 827.49 | Unit | 10 | 130 | 25.1 | 4 | Positive |
| sp\|Q12792\|TWF1_HUMAN | DYDSFVLPLLEDK.light | FALSE | 777.39 | Unit | 714.40 | Unit | 10 | 130 | 25.1 | 4 | Positive |
| sp\|Q12792\|TWF1_HUMAN | INEVQTDVGVDTK.light | FALSE | 709.36 | Unit | 1061.55 | Unit | 10 | 130 | 23 | 4 | Positive |
| sp\|Q12792\|TWF1_HUMAN | INEVQTDVGVDTK.light | FALSE | 709.36 | Unit | 962.48 | Unit | 10 | 130 | 23 | 4 | Positive |
| sp\|Q12792\|TWF1_HUMAN | INEVQTDVGVDTK.light | FALSE | 709.36 | Unit | 834.42 | Unit | 10 | 130 | 23 | 4 | Positive |
| sp\|Q12792\|TWF1_HUMAN | INEVQTDVGVDTK.light | FALSE | 709.36 | Unit | 248.16 | Unit | 10 | 130 | 23 | 4 | Positive |
| sp\|P12277\|KCRB_HUMAN | VLTPELYAELR.light | FALSE | 652.37 | Unit | 1091.57 | Unit | 10 | 130 | 21.2 | 4 | Positive |
| sp\|P12277\|KCRB_HUMAN | VLTPELYAELR.light | FALSE | 652.37 | Unit | 990.53 | Unit | 10 | 130 | 21.2 | 4 | Positive |
| sp\|P12277\|KCRB_HUMAN | VLTPELYAELR.light | FALSE | 652.37 | Unit | 764.43 | Unit | 10 | 130 | 21.2 | 4 | Positive |
| sp\|P12277\|KCRB_HUMAN | VLTPELYAELR.light | FALSE | 652.37 | Unit | 651.35 | Unit | 10 | 130 | 21.2 | 4 | Positive |
| sp\|P12277\|KCRB_HUMAN | LAVEALSSLDGDLAGR.light | FALSE | 793.92 | Unit | 1103.57 | Unit | 10 | 130 | 25.6 | 4 | Positive |
| sp\|P12277\|KCRB_HUMAN | LAVEALSSLDGDLAGR.light | FALSE | 793.92 | Unit | 990.49 | Unit | 10 | 130 | 25.6 | 4 | Positive |
| sp\|P12277\|KCRB_HUMAN | LAVEALSSLDGDLAGR.light | FALSE | 793.92 | Unit | 903.45 | Unit | 10 | 130 | 25.6 | 4 | Positive |
| sp\|P12277\|KCRB_HUMAN | LAVEALSSLDGDLAGR.light | FALSE | 793.92 | Unit | 588.31 | Unit | 10 | 130 | 25.6 | 4 | Positive |
| sp\|P02144\|MYG_HUMAN | VEADIPGHGQEVLIR.light | FALSE | 816.94 | Unit | 1105.61 | Unit | 10 | 130 | 26.3 | 4 | Positive |
| sp\|P02144\|MYG_HUMAN | VEADIPGHGQEVLIR.light | FALSE | 816.94 | Unit | 1008.56 | Unit | 10 | 130 | 26.3 | 4 | Positive |
| sp\|P02144\|MYG_HUMAN | VEADIPGHGQEVLIR.light | FALSE | 816.94 | Unit | 814.48 | Unit | 10 | 130 | 26.3 | 4 | Positive |
| sp\|P02144\|MYG_HUMAN | VEADIPGHGQEVLIR.light | FALSE | 816.94 | Unit | 175.12 | Unit | 10 | 130 | 26.3 | 4 | Positive |
| sp\|P02144\|MYG_HUMAN | GHPETLEK.light | FALSE | 455.74 | Unit | 716.38 | Unit | 10 | 130 | 15.1 | 4 | Positive |
| sp\|P02144\|MYG_HUMAN | GHPETLEK.light | FALSE | 455.74 | Unit | 490.29 | Unit | 10 | 130 | 15.1 | 4 | Positive |
| sp\|P02144\|MYG_HUMAN | GHPETLEK.light | FALSE | 455.74 | Unit | 276.16 | Unit | 10 | 130 | 15.1 | 4 | Positive |
| sp\|P02144\|MYG_HUMAN | GHPETLEK.light | FALSE | 455.74 | Unit | 147.11 | Unit | 10 | 130 | 15.1 | 4 | Positive |
| sp\|P13861\|KAP2_HUMAN | DGGNQEVEIAR.light | FALSE | 594.29 | Unit | 716.39 | Unit | 10 | 130 | 19.4 | 4 | Positive |
| sp\|P13861\|KAP2_HUMAN | DGGNQEVEIAR.light | FALSE | 594.29 | Unit | 587.35 | Unit | 10 | 130 | 19.4 | 4 | Positive |
| sp\|P13861\|KAP2_HUMAN | DGGNQEVEIAR.light | FALSE | 594.29 | Unit | 488.28 | Unit | 10 | 130 | 19.4 | 4 | Positive |
| sp\|P13861\|KAP2_HUMAN | DGGNQEVEIAR.light | FALSE | 594.29 | Unit | 175.12 | Unit | 10 | 130 | 19.4 | 4 | Positive |
| sp\|P13861\|KAP2_HUMAN | LLGPC[+57.0]MDIMK.light | FALSE | 589.29 | Unit | 951.41 | Unit | 10 | 130 | 19.3 | 4 | Positive |
| sp\|P13861\|KAP2_HUMAN | LLGPC[+57.0]MDIMK.light | FALSE | 589.29 | Unit | 894.39 | Unit | 10 | 130 | 19.3 | 4 | Positive |
| sp\|P13861\|KAP2_HUMAN | LLGPC[+57.0]MDIMK.light | FALSE | 589.29 | Unit | 797.34 | Unit | 10 | 130 | 19.3 | 4 | Positive |
| sp\|P13861\|KAP2_HUMAN | LLGPC[+57.0]MDIMK.light | FALSE | 589.29 | Unit | 147.11 | Unit | 10 | 130 | 19.3 | 4 | Positive |
| sp\|Q9NP58\|ABCB6_HUMAN | AVDSLLNFETVK.light | FALSE | 668.36 | Unit | 1165.61 | Unit | 10 | 130 | 21.7 | 4 | Positive |
| sp\|Q9NP58\|ABCB6_HUMAN | AVDSLLNFETVK.light | FALSE | 668.36 | Unit | 850.47 | Unit | 10 | 130 | 21.7 | 4 | Positive |
| sp\|Q9NP58\|ABCB6_HUMAN | AVDSLLNFETVK.light | FALSE | 668.36 | Unit | 737.38 | Unit | 10 | 130 | 21.7 | 4 | Positive |
| sp\|Q9NP58\|ABCB6_HUMAN | AVDSLLNFETVK.light | FALSE | 668.36 | Unit | 147.11 | Unit | 10 | 130 | 21.7 | 4 | Positive |
| sp\|Q9NP58\|ABCB6_HUMAN | IDGQDISQVTQASLR.light | FALSE | 815.92 | Unit | 1102.62 | Unit | 10 | 130 | 26.3 | 4 | Positive |
| sp\|Q9NP58\|ABCB6_HUMAN | IDGQDISQVTQASLR.light | FALSE | 815.92 | Unit | 989.54 | Unit | 10 | 130 | 26.3 | 4 | Positive |
| sp\|Q9NP58\|ABCB6_HUMAN | IDGQDISQVTQASLR.light | FALSE | 815.92 | Unit | 902.51 | Unit | 10 | 130 | 26.3 | 4 | Positive |
| sp\|Q9NP58\|ABCB6_HUMAN | IDGQDISQVTQASLR.light | FALSE | 815.92 | Unit | 675.38 | Unit | 10 | 130 | 26.3 | 4 | Positive |
| sp\|P26358\|DNMT1_HUMAN | LAGVTLGQR.light | FALSE | 457.77 | Unit | 801.46 | Unit | 10 | 130 | 15.2 | 4 | Positive |
| sp\|P26358\|DNMT1_HUMAN | LAGVTLGQR.light | FALSE | 457.77 | Unit | 730.42 | Unit | 10 | 130 | 15.2 | 4 | Positive |
| sp\|P26358\|DNMT1_HUMAN | LAGVTLGQR.light | FALSE | 457.77 | Unit | 574.33 | Unit | 10 | 130 | 15.2 | 4 | Positive |
| sp\|P26358\|DNMT1_HUMAN | LAGVTLGQR.light | FALSE | 457.77 | Unit | 175.12 | Unit | 10 | 130 | 15.2 | 4 | Positive |
| sp\|P26358\|DNMT1_HUMAN | VLEQLEDLDSR.light | FALSE | 658.84 | Unit | 1104.52 | Unit | 10 | 130 | 21.4 | 4 | Positive |
| sp\|P26358\|DNMT1_HUMAN | VLEQLEDLDSR.light | FALSE | 658.84 | Unit | 847.42 | Unit | 10 | 130 | 21.4 | 4 | Positive |
| sp\|P26358\|DNMT1_HUMAN | VLEQLEDLDSR.light | FALSE | 658.84 | Unit | 734.33 | Unit | 10 | 130 | 21.4 | 4 | Positive |
| sp\|P26358\|DNMT1_HUMAN | VLEQLEDLDSR.light | FALSE | 658.84 | Unit | 262.15 | Unit | 10 | 130 | 21.4 | 4 | Positive |
| sp\|Q92597\|NDRG1_HUMAN | SIIGMGTGAGAYILTR.light | FALSE | 790.93 | Unit | 1079.58 | Unit | 10 | 130 | 25.5 | 4 | Positive |
| sp\|Q92597\|NDRG1_HUMAN | SIIGMGTGAGAYILTR.light | FALSE | 790.93 | Unit | 921.52 | Unit | 10 | 130 | 25.5 | 4 | Positive |
| sp\|Q92597\|NDRG1_HUMAN | SIIGMGTGAGAYILTR.light | FALSE | 790.93 | Unit | 793.46 | Unit | 10 | 130 | 25.5 | 4 | Positive |
| sp\|Q92597\|NDRG1_HUMAN | SIIGMGTGAGAYILTR.light | FALSE | 790.93 | Unit | 175.12 | Unit | 10 | 130 | 25.5 | 4 | Positive |
| sp\|Q92597\|NDRG1_HUMAN | TASGSSVTSLDGTR.light | FALSE | 669.83 | Unit | 1166.56 | Unit | 10 | 130 | 21.8 | 4 | Positive |
| sp\|Q92597\|NDRG1_HUMAN | TASGSSVTSLDGTR.light | FALSE | 669.83 | Unit | 935.48 | Unit | 10 | 130 | 21.8 | 4 | Positive |
| sp\|Q92597\|NDRG1_HUMAN | TASGSSVTSLDGTR.light | FALSE | 669.83 | Unit | 749.38 | Unit | 10 | 130 | 21.8 | 4 | Positive |
| sp\|Q92597\|NDRG1_HUMAN | TASGSSVTSLDGTR.light | FALSE | 669.83 | Unit | 333.19 | Unit | 10 | 130 | 21.8 | 4 | Positive |

Table S.4. MRM Transition List for AS Protein Panel

| Compound Group | Compound Name | ISTD? | Precursor Ion | MS1 Res | Product Ion | MS2 Res | Dwell | Fragmentor | Collision Energy | Cell Accelerator Voltage | Polarity |
| --- | --- | --- | --- | --- | --- | --- | --- | --- | --- | --- | --- |
| sp\|Q8NBS9\|TXND5_HUMAN | VDC[+57.0]TQHYELC[+57.0]SGNQVR.light | FALSE | 983.43 | Unit | 1062.50 | Unit | 10 | 130 | 31.5 | 4 | Positive |
| sp\|Q8NBS9\|TXND5_HUMAN | VDC[+57.0]TQHYELC[+57.0]SGNQVR.light | FALSE | 983.43 | Unit | 933.46 | Unit | 10 | 130 | 31.5 | 4 | Positive |
| sp\|Q8NBS9\|TXND5_HUMAN | VDC[+57.0]TQHYELC[+57.0]SGNQVR.light | FALSE | 983.43 | Unit | 820.37 | Unit | 10 | 130 | 31.5 | 4 | Positive |
| sp\|Q8NBS9\|TXND5_HUMAN | VDC[+57.0]TQHYELC[+57.0]SGNQVR.light | FALSE | 983.43 | Unit | 175.12 | Unit | 10 | 130 | 31.5 | 4 | Positive |
| sp\|Q8NBS9\|TXND5_HUMAN | IAEVDC[+57.0]TAER.light | FALSE | 582.27 | Unit | 1050.45 | Unit | 10 | 130 | 19.1 | 4 | Positive |
| sp\|Q8NBS9\|TXND5_HUMAN | IAEVDC[+57.0]TAER.light | FALSE | 582.27 | Unit | 979.41 | Unit | 10 | 130 | 19.1 | 4 | Positive |
| sp\|Q8NBS9\|TXND5_HUMAN | IAEVDC[+57.0]TAER.light | FALSE | 582.27 | Unit | 850.37 | Unit | 10 | 130 | 19.1 | 4 | Positive |
| sp\|Q8NBS9\|TXND5_HUMAN | IAEVDC[+57.0]TAER.light | FALSE | 582.27 | Unit | 751.30 | Unit | 10 | 130 | 19.1 | 4 | Positive |
| sp\|P27797\|CALR_HUMAN | FYALSASFEPFSNK.light | FALSE | 804.39 | Unit | 1113.52 | Unit | 10 | 130 | 25.9 | 4 | Positive |
| sp\|P27797\|CALR_HUMAN | FYALSASFEPFSNK.light | FALSE | 804.39 | Unit | 955.45 | Unit | 10 | 130 | 25.9 | 4 | Positive |
| sp\|P27797\|CALR_HUMAN | FYALSASFEPFSNK.light | FALSE | 804.39 | Unit | 592.31 | Unit | 10 | 130 | 25.9 | 4 | Positive |
| sp\|P27797\|CALR_HUMAN | FYALSASFEPFSNK.light | FALSE | 804.39 | Unit | 348.19 | Unit | 10 | 130 | 25.9 | 4 | Positive |
| sp\|P27797\|CALR_HUMAN | GQTLVVQFTVK.light | FALSE | 610.36 | Unit | 1034.62 | Unit | 10 | 130 | 19.9 | 4 | Positive |
| sp\|P27797\|CALR_HUMAN | GQTLVVQFTVK.light | FALSE | 610.36 | Unit | 933.58 | Unit | 10 | 130 | 19.9 | 4 | Positive |
| sp\|P27797\|CALR_HUMAN | GQTLVVQFTVK.light | FALSE | 610.36 | Unit | 820.49 | Unit | 10 | 130 | 19.9 | 4 | Positive |
| sp\|P27797\|CALR_HUMAN | GQTLVVQFTVK.light | FALSE | 610.36 | Unit | 721.42 | Unit | 10 | 130 | 19.9 | 4 | Positive |
| sp\|P11388\|TOP2A_HUMAN | IFDEILVNAADNK.light | FALSE | 731.38 | Unit | 844.45 | Unit | 10 | 130 | 23.7 | 4 | Positive |
| sp\|P11388\|TOP2A_HUMAN | IFDEILVNAADNK.light | FALSE | 731.38 | Unit | 731.37 | Unit | 10 | 130 | 23.7 | 4 | Positive |
| sp\|P11388\|TOP2A_HUMAN | IFDEILVNAADNK.light | FALSE | 731.38 | Unit | 376.18 | Unit | 10 | 130 | 23.7 | 4 | Positive |
| sp\|P11388\|TOP2A_HUMAN | IFDEILVNAADNK.light | FALSE | 731.38 | Unit | 261.16 | Unit | 10 | 130 | 23.7 | 4 | Positive |
| sp\|P11388\|TOP2A_HUMAN | SVVSDLEADDVK.light | FALSE | 638.82 | Unit | 1090.53 | Unit | 10 | 130 | 20.8 | 4 | Positive |
| sp\|P11388\|TOP2A_HUMAN | SVVSDLEADDVK.light | FALSE | 638.82 | Unit | 991.46 | Unit | 10 | 130 | 20.8 | 4 | Positive |
| sp\|P11388\|TOP2A_HUMAN | SVVSDLEADDVK.light | FALSE | 638.82 | Unit | 904.43 | Unit | 10 | 130 | 20.8 | 4 | Positive |
| sp\|P11388\|TOP2A_HUMAN | SVVSDLEADDVK.light | FALSE | 638.82 | Unit | 147.11 | Unit | 10 | 130 | 20.8 | 4 | Positive |
| sp\|Q05655\|KPCD_HUMAN | LLAEALNQVTQR.light | FALSE | 678.39 | Unit | 1129.60 | Unit | 10 | 130 | 22 | 4 | Positive |
| sp\|Q05655\|KPCD_HUMAN | LLAEALNQVTQR.light | FALSE | 678.39 | Unit | 929.52 | Unit | 10 | 130 | 22 | 4 | Positive |
| sp\|Q05655\|KPCD_HUMAN | LLAEALNQVTQR.light | FALSE | 678.39 | Unit | 858.48 | Unit | 10 | 130 | 22 | 4 | Positive |
| sp\|Q05655\|KPCD_HUMAN | LLAEALNQVTQR.light | FALSE | 678.39 | Unit | 745.40 | Unit | 10 | 130 | 22 | 4 | Positive |
| sp\|Q05655\|KPCD_HUMAN | VLLGELK.light | FALSE | 386.25 | Unit | 672.43 | Unit | 10 | 130 | 13 | 4 | Positive |
| sp\|Q05655\|KPCD_HUMAN | VLLGELK.light | FALSE | 386.25 | Unit | 559.34 | Unit | 10 | 130 | 13 | 4 | Positive |
| sp\|Q05655\|KPCD_HUMAN | VLLGELK.light | FALSE | 386.25 | Unit | 446.26 | Unit | 10 | 130 | 13 | 4 | Positive |
| sp\|Q05655\|KPCD_HUMAN | VLLGELK.light | FALSE | 386.25 | Unit | 260.20 | Unit | 10 | 130 | 13 | 4 | Positive |
| sp\|P07237\|PDIA1_HUMAN | VDATEESDLAQQYGVR.light | FALSE | 890.92 | Unit | 821.43 | Unit | 10 | 130 | 28.6 | 4 | Positive |
| sp\|P07237\|PDIA1_HUMAN | VDATEESDLAQQYGVR.light | FALSE | 890.92 | Unit | 750.39 | Unit | 10 | 130 | 28.6 | 4 | Positive |
| sp\|P07237\|PDIA1_HUMAN | VDATEESDLAQQYGVR.light | FALSE | 890.92 | Unit | 331.21 | Unit | 10 | 130 | 28.6 | 4 | Positive |
| sp\|P07237\|PDIA1_HUMAN | VDATEESDLAQQYGVR.light | FALSE | 890.92 | Unit | 175.12 | Unit | 10 | 130 | 28.6 | 4 | Positive |
| sp\|P07237\|PDIA1_HUMAN | ILEFFGLK.light | FALSE | 483.79 | Unit | 853.48 | Unit | 10 | 130 | 16 | 4 | Positive |
| sp\|P07237\|PDIA1_HUMAN | ILEFFGLK.light | FALSE | 483.79 | Unit | 740.40 | Unit | 10 | 130 | 16 | 4 | Positive |
| sp\|P07237\|PDIA1_HUMAN | ILEFFGLK.light | FALSE | 483.79 | Unit | 611.36 | Unit | 10 | 130 | 16 | 4 | Positive |
| sp\|P07237\|PDIA1_HUMAN | ILEFFGLK.light | FALSE | 483.79 | Unit | 464.29 | Unit | 10 | 130 | 16 | 4 | Positive |
| sp\|Q92820\|GGH_HUMAN | DYEILFK.light | FALSE | 464.24 | Unit | 649.39 | Unit | 10 | 130 | 15.4 | 4 | Positive |
| sp\|Q92820\|GGH_HUMAN | DYEILFK.light | FALSE | 464.24 | Unit | 407.27 | Unit | 10 | 130 | 15.4 | 4 | Positive |
| sp\|Q92820\|GGH_HUMAN | DYEILFK.light | FALSE | 464.24 | Unit | 294.18 | Unit | 10 | 130 | 15.4 | 4 | Positive |
| sp\|Q92820\|GGH_HUMAN | DYEILFK.light | FALSE | 464.24 | Unit | 147.11 | Unit | 10 | 130 | 15.4 | 4 | Positive |
| sp\|Q92820\|GGH_HUMAN | NLDGISHAPNAVK.light | FALSE | 668.35 | Unit | 1108.57 | Unit | 10 | 130 | 21.7 | 4 | Positive |
| sp\|Q92820\|GGH_HUMAN | NLDGISHAPNAVK.light | FALSE | 668.35 | Unit | 993.55 | Unit | 10 | 130 | 21.7 | 4 | Positive |
| sp\|Q92820\|GGH_HUMAN | NLDGISHAPNAVK.light | FALSE | 668.35 | Unit | 823.44 | Unit | 10 | 130 | 21.7 | 4 | Positive |
| sp\|Q92820\|GGH_HUMAN | NLDGISHAPNAVK.light | FALSE | 668.35 | Unit | 528.31 | Unit | 10 | 130 | 21.7 | 4 | Positive |
| sp\|P07099\|HYEP_HUMAN | DVELLYPVK.light | FALSE | 538.31 | Unit | 861.51 | Unit | 10 | 130 | 17.7 | 4 | Positive |
| sp\|P07099\|HYEP_HUMAN | DVELLYPVK.light | FALSE | 538.31 | Unit | 506.30 | Unit | 10 | 130 | 17.7 | 4 | Positive |
| sp\|P07099\|HYEP_HUMAN | DVELLYPVK.light | FALSE | 538.31 | Unit | 343.23 | Unit | 10 | 130 | 17.7 | 4 | Positive |
| sp\|P07099\|HYEP_HUMAN | DVELLYPVK.light | FALSE | 538.31 | Unit | 147.11 | Unit | 10 | 130 | 17.7 | 4 | Positive |
| sp\|P07099\|HYEP_HUMAN | FSTWTNTEFR.light | FALSE | 644.80 | Unit | 1054.50 | Unit | 10 | 130 | 21 | 4 | Positive |
| sp\|P07099\|HYEP_HUMAN | FSTWTNTEFR.light | FALSE | 644.80 | Unit | 953.45 | Unit | 10 | 130 | 21 | 4 | Positive |
| sp\|P07099\|HYEP_HUMAN | FSTWTNTEFR.light | FALSE | 644.80 | Unit | 767.37 | Unit | 10 | 130 | 21 | 4 | Positive |
| sp\|P07099\|HYEP_HUMAN | FSTWTNTEFR.light | FALSE | 644.80 | Unit | 666.32 | Unit | 10 | 130 | 21 | 4 | Positive |
| sp\|P49321\|NASP_HUMAN | EAQLYAAQAHLK.light | FALSE | 671.86 | Unit | 1014.57 | Unit | 10 | 130 | 21.8 | 4 | Positive |
| sp\|P49321\|NASP_HUMAN | EAQLYAAQAHLK.light | FALSE | 671.86 | Unit | 901.49 | Unit | 10 | 130 | 21.8 | 4 | Positive |
| sp\|P49321\|NASP_HUMAN | EAQLYAAQAHLK.light | FALSE | 671.86 | Unit | 738.43 | Unit | 10 | 130 | 21.8 | 4 | Positive |
| sp\|P49321\|NASP_HUMAN | EAQLYAAQAHLK.light | FALSE | 671.86 | Unit | 667.39 | Unit | 10 | 130 | 21.8 | 4 | Positive |
| sp\|P49321\|NASP_HUMAN | SGNVAELALK.light | FALSE | 501.28 | Unit | 743.47 | Unit | 10 | 130 | 16.5 | 4 | Positive |
| sp\|P49321\|NASP_HUMAN | SGNVAELALK.light | FALSE | 501.28 | Unit | 644.40 | Unit | 10 | 130 | 16.5 | 4 | Positive |
| sp\|P49321\|NASP_HUMAN | SGNVAELALK.light | FALSE | 501.28 | Unit | 573.36 | Unit | 10 | 130 | 16.5 | 4 | Positive |
| sp\|P49321\|NASP_HUMAN | SGNVAELALK.light | FALSE | 501.28 | Unit | 147.11 | Unit | 10 | 130 | 16.5 | 4 | Positive |
| sp\|O00116\|ADAS_HUMAN | NIYGNIEDLVVHIK.light | FALSE | 813.95 | Unit | 952.55 | Unit | 10 | 130 | 26.2 | 4 | Positive |
| sp\|O00116\|ADAS_HUMAN | NIYGNIEDLVVHIK.light | FALSE | 813.95 | Unit | 496.32 | Unit | 10 | 130 | 26.2 | 4 | Positive |
| sp\|O00116\|ADAS_HUMAN | NIYGNIEDLVVHIK.light | FALSE | 813.95 | Unit | 397.26 | Unit | 10 | 130 | 26.2 | 4 | Positive |
| sp\|O00116\|ADAS_HUMAN | NIYGNIEDLVVHIK.light | FALSE | 813.95 | Unit | 147.11 | Unit | 10 | 130 | 26.2 | 4 | Positive |
| sp\|O00116\|ADAS_HUMAN | YGSVAFPNFEQGVAC[+57.0]LR.light | FALSE | 957.96 | Unit | 1079.53 | Unit | 10 | 130 | 30.7 | 4 | Positive |
| sp\|O00116\|ADAS_HUMAN | YGSVAFPNFEQGVAC[+57.0]LR.light | FALSE | 957.96 | Unit | 803.42 | Unit | 10 | 130 | 30.7 | 4 | Positive |
| sp\|O00116\|ADAS_HUMAN | YGSVAFPNFEQGVAC[+57.0]LR.light | FALSE | 957.96 | Unit | 675.36 | Unit | 10 | 130 | 30.7 | 4 | Positive |
| sp\|O00116\|ADAS_HUMAN | YGSVAFPNFEQGVAC[+57.0]LR.light | FALSE | 957.96 | Unit | 175.12 | Unit | 10 | 130 | 30.7 | 4 | Positive |
| sp\|Q8TEM1\|PO210_HUMAN | ELYLEDSPLELK.light | FALSE | 724.88 | Unit | 1043.56 | Unit | 10 | 130 | 23.5 | 4 | Positive |
| sp\|Q8TEM1\|PO210_HUMAN | ELYLEDSPLELK.light | FALSE | 724.88 | Unit | 930.48 | Unit | 10 | 130 | 23.5 | 4 | Positive |
| sp\|Q8TEM1\|PO210_HUMAN | ELYLEDSPLELK.light | FALSE | 724.88 | Unit | 801.44 | Unit | 10 | 130 | 23.5 | 4 | Positive |
| sp\|Q8TEM1\|PO210_HUMAN | ELYLEDSPLELK.light | FALSE | 724.88 | Unit | 686.41 | Unit | 10 | 130 | 23.5 | 4 | Positive |
| sp\|Q8TEM1\|PO210_HUMAN | ITIAAYLPLK.light | FALSE | 551.85 | Unit | 888.56 | Unit | 10 | 130 | 18.1 | 4 | Positive |
| sp\|Q8TEM1\|PO210_HUMAN | ITIAAYLPLK.light | FALSE | 551.85 | Unit | 775.47 | Unit | 10 | 130 | 18.1 | 4 | Positive |
| sp\|Q8TEM1\|PO210_HUMAN | ITIAAYLPLK.light | FALSE | 551.85 | Unit | 704.43 | Unit | 10 | 130 | 18.1 | 4 | Positive |
| sp\|Q8TEM1\|PO210_HUMAN | ITIAAYLPLK.light | FALSE | 551.85 | Unit | 357.25 | Unit | 10 | 130 | 18.1 | 4 | Positive |
| sp\|O75795\|UDB17_HUMAN | LEVYPTSLTK.light | FALSE | 575.82 | Unit | 908.51 | Unit | 10 | 130 | 18.9 | 4 | Positive |
| sp\|O75795\|UDB17_HUMAN | LEVYPTSLTK.light | FALSE | 575.82 | Unit | 809.44 | Unit | 10 | 130 | 18.9 | 4 | Positive |
| sp\|O75795\|UDB17_HUMAN | LEVYPTSLTK.light | FALSE | 575.82 | Unit | 646.38 | Unit | 10 | 130 | 18.9 | 4 | Positive |
| sp\|O75795\|UDB17_HUMAN | LEVYPTSLTK.light | FALSE | 575.82 | Unit | 147.11 | Unit | 10 | 130 | 18.9 | 4 | Positive |
| sp\|O75795\|UDB17_HUMAN | NDLEDFFMK.light | FALSE | 579.76 | Unit | 929.44 | Unit | 10 | 130 | 19 | 4 | Positive |
| sp\|O75795\|UDB17_HUMAN | NDLEDFFMK.light | FALSE | 579.76 | Unit | 816.36 | Unit | 10 | 130 | 19 | 4 | Positive |
| sp\|O75795\|UDB17_HUMAN | NDLEDFFMK.light | FALSE | 579.76 | Unit | 278.15 | Unit | 10 | 130 | 19 | 4 | Positive |
| sp\|O75795\|UDB17_HUMAN | NDLEDFFMK.light | FALSE | 579.76 | Unit | 147.11 | Unit | 10 | 130 | 19 | 4 | Positive |
| sp\|P30533\|AMRP_HUMAN | LAELHADLK.light | FALSE | 505.29 | Unit | 896.48 | Unit | 10 | 130 | 16.7 | 4 | Positive |
| sp\|P30533\|AMRP_HUMAN | LAELHADLK.light | FALSE | 505.29 | Unit | 583.32 | Unit | 10 | 130 | 16.7 | 4 | Positive |
| sp\|P30533\|AMRP_HUMAN | LAELHADLK.light | FALSE | 505.29 | Unit | 260.20 | Unit | 10 | 130 | 16.7 | 4 | Positive |
| sp\|P30533\|AMRP_HUMAN | LAELHADLK.light | FALSE | 505.29 | Unit | 147.11 | Unit | 10 | 130 | 16.7 | 4 | Positive |
| sp\|P30533\|AMRP_HUMAN | FSGEELDK.light | FALSE | 462.72 | Unit | 777.36 | Unit | 10 | 130 | 15.3 | 4 | Positive |
| sp\|P30533\|AMRP_HUMAN | FSGEELDK.light | FALSE | 462.72 | Unit | 690.33 | Unit | 10 | 130 | 15.3 | 4 | Positive |
| sp\|P30533\|AMRP_HUMAN | FSGEELDK.light | FALSE | 462.72 | Unit | 262.14 | Unit | 10 | 130 | 15.3 | 4 | Positive |
| sp\|P30533\|AMRP_HUMAN | FSGEELDK.light | FALSE | 462.72 | Unit | 147.11 | Unit | 10 | 130 | 15.3 | 4 | Positive |
| sp\|Q9UM54\|MYO6_HUMAN | TVYSHLFDHVVNR.light | FALSE | 793.91 | Unit | 1136.60 | Unit | 10 | 130 | 25.6 | 4 | Positive |
| sp\|Q9UM54\|MYO6_HUMAN | TVYSHLFDHVVNR.light | FALSE | 793.91 | Unit | 999.54 | Unit | 10 | 130 | 25.6 | 4 | Positive |
| sp\|Q9UM54\|MYO6_HUMAN | TVYSHLFDHVVNR.light | FALSE | 793.91 | Unit | 886.45 | Unit | 10 | 130 | 25.6 | 4 | Positive |
| sp\|Q9UM54\|MYO6_HUMAN | TVYSHLFDHVVNR.light | FALSE | 793.91 | Unit | 624.36 | Unit | 10 | 130 | 25.6 | 4 | Positive |
| sp\|Q9UM54\|MYO6_HUMAN | EEQELYQK.light | FALSE | 533.76 | Unit | 680.36 | Unit | 10 | 130 | 17.5 | 4 | Positive |
| sp\|Q9UM54\|MYO6_HUMAN | EEQELYQK.light | FALSE | 533.76 | Unit | 551.32 | Unit | 10 | 130 | 17.5 | 4 | Positive |
| sp\|Q9UM54\|MYO6_HUMAN | EEQELYQK.light | FALSE | 533.76 | Unit | 438.23 | Unit | 10 | 130 | 17.5 | 4 | Positive |
| sp\|Q9UM54\|MYO6_HUMAN | EEQELYQK.light | FALSE | 533.76 | Unit | 147.11 | Unit | 10 | 130 | 17.5 | 4 | Positive |
| sp\|P27144\|KAD4_HUMAN | AVILGPPGSGK.light | FALSE | 498.30 | Unit | 712.40 | Unit | 10 | 130 | 16.4 | 4 | Positive |
| sp\|P27144\|KAD4_HUMAN | AVILGPPGSGK.light | FALSE | 498.30 | Unit | 599.31 | Unit | 10 | 130 | 16.4 | 4 | Positive |
| sp\|P27144\|KAD4_HUMAN | AVILGPPGSGK.light | FALSE | 498.30 | Unit | 542.29 | Unit | 10 | 130 | 16.4 | 4 | Positive |
| sp\|P27144\|KAD4_HUMAN | AVILGPPGSGK.light | FALSE | 498.30 | Unit | 445.24 | Unit | 10 | 130 | 16.4 | 4 | Positive |
| sp\|P27144\|KAD4_HUMAN | ASTEVGEMAK.light | FALSE | 511.74 | Unit | 763.37 | Unit | 10 | 130 | 16.9 | 4 | Positive |
| sp\|P27144\|KAD4_HUMAN | ASTEVGEMAK.light | FALSE | 511.74 | Unit | 634.32 | Unit | 10 | 130 | 16.9 | 4 | Positive |
| sp\|P27144\|KAD4_HUMAN | ASTEVGEMAK.light | FALSE | 511.74 | Unit | 535.25 | Unit | 10 | 130 | 16.9 | 4 | Positive |
| sp\|P27144\|KAD4_HUMAN | ASTEVGEMAK.light | FALSE | 511.74 | Unit | 147.11 | Unit | 10 | 130 | 16.9 | 4 | Positive |
| sp\|P13807\|GYS1_HUMAN | VGGIYTVLQTK.light | FALSE | 589.84 | Unit | 852.48 | Unit | 10 | 130 | 19.3 | 4 | Positive |
| sp\|P13807\|GYS1_HUMAN | VGGIYTVLQTK.light | FALSE | 589.84 | Unit | 689.42 | Unit | 10 | 130 | 19.3 | 4 | Positive |
| sp\|P13807\|GYS1_HUMAN | VGGIYTVLQTK.light | FALSE | 589.84 | Unit | 489.30 | Unit | 10 | 130 | 19.3 | 4 | Positive |
| sp\|P13807\|GYS1_HUMAN | VGGIYTVLQTK.light | FALSE | 589.84 | Unit | 147.11 | Unit | 10 | 130 | 19.3 | 4 | Positive |
| sp\|P13807\|GYS1_HUMAN | TQVELLEAPTPALK.light | FALSE | 755.43 | Unit | 939.55 | Unit | 10 | 130 | 24.4 | 4 | Positive |
| sp\|P13807\|GYS1_HUMAN | TQVELLEAPTPALK.light | FALSE | 755.43 | Unit | 826.47 | Unit | 10 | 130 | 24.4 | 4 | Positive |
| sp\|P13807\|GYS1_HUMAN | TQVELLEAPTPALK.light | FALSE | 755.43 | Unit | 626.39 | Unit | 10 | 130 | 24.4 | 4 | Positive |
| sp\|P13807\|GYS1_HUMAN | TQVELLEAPTPALK.light | FALSE | 755.43 | Unit | 428.29 | Unit | 10 | 130 | 24.4 | 4 | Positive |
| sp\|Q08209\|PP2BA_HUMAN | GFSPQHK.light | FALSE | 400.71 | Unit | 596.32 | Unit | 10 | 130 | 13.4 | 4 | Positive |
| sp\|Q08209\|PP2BA_HUMAN | GFSPQHK.light | FALSE | 400.71 | Unit | 509.28 | Unit | 10 | 130 | 13.4 | 4 | Positive |
| sp\|Q08209\|PP2BA_HUMAN | GFSPQHK.light | FALSE | 400.71 | Unit | 284.17 | Unit | 10 | 130 | 13.4 | 4 | Positive |
| sp\|Q08209\|PP2BA_HUMAN | GFSPQHK.light | FALSE | 400.71 | Unit | 147.11 | Unit | 10 | 130 | 13.4 | 4 | Positive |
| sp\|Q08209\|PP2BA_HUMAN | ITSFEEAK.light | FALSE | 462.74 | Unit | 811.38 | Unit | 10 | 130 | 15.3 | 4 | Positive |
| sp\|Q08209\|PP2BA_HUMAN | ITSFEEAK.light | FALSE | 462.74 | Unit | 710.34 | Unit | 10 | 130 | 15.3 | 4 | Positive |
| sp\|Q08209\|PP2BA_HUMAN | ITSFEEAK.light | FALSE | 462.74 | Unit | 218.15 | Unit | 10 | 130 | 15.3 | 4 | Positive |
| sp\|Q08209\|PP2BA_HUMAN | ITSFEEAK.light | FALSE | 462.74 | Unit | 147.11 | Unit | 10 | 130 | 15.3 | 4 | Positive |
| sp\|P02786\|TFR1_HUMAN | LAVDEEENADNNTK.light | FALSE | 781.35 | Unit | 1163.48 | Unit | 10 | 130 | 25.2 | 4 | Positive |
| sp\|P02786\|TFR1_HUMAN | LAVDEEENADNNTK.light | FALSE | 781.35 | Unit | 1034.44 | Unit | 10 | 130 | 25.2 | 4 | Positive |
| sp\|P02786\|TFR1_HUMAN | LAVDEEENADNNTK.light | FALSE | 781.35 | Unit | 905.40 | Unit | 10 | 130 | 25.2 | 4 | Positive |
| sp\|P02786\|TFR1_HUMAN | LAVDEEENADNNTK.light | FALSE | 781.35 | Unit | 776.35 | Unit | 10 | 130 | 25.2 | 4 | Positive |
| sp\|P02786\|TFR1_HUMAN | LTTDFGNAEK.light | FALSE | 548.27 | Unit | 982.45 | Unit | 10 | 130 | 18 | 4 | Positive |
| sp\|P02786\|TFR1_HUMAN | LTTDFGNAEK.light | FALSE | 548.27 | Unit | 881.40 | Unit | 10 | 130 | 18 | 4 | Positive |
| sp\|P02786\|TFR1_HUMAN | LTTDFGNAEK.light | FALSE | 548.27 | Unit | 780.35 | Unit | 10 | 130 | 18 | 4 | Positive |
| sp\|P02786\|TFR1_HUMAN | LTTDFGNAEK.light | FALSE | 548.27 | Unit | 665.33 | Unit | 10 | 130 | 18 | 4 | Positive |
| sp\|O95573\|ACSL3_HUMAN | NLFILAYNYK.light | FALSE | 629.85 | Unit | 884.49 | Unit | 10 | 130 | 20.5 | 4 | Positive |
| sp\|O95573\|ACSL3_HUMAN | NLFILAYNYK.light | FALSE | 629.85 | Unit | 771.40 | Unit | 10 | 130 | 20.5 | 4 | Positive |
| sp\|O95573\|ACSL3_HUMAN | NLFILAYNYK.light | FALSE | 629.85 | Unit | 658.32 | Unit | 10 | 130 | 20.5 | 4 | Positive |
| sp\|O95573\|ACSL3_HUMAN | NLFILAYNYK.light | FALSE | 629.85 | Unit | 424.22 | Unit | 10 | 130 | 20.5 | 4 | Positive |
| sp\|O95573\|ACSL3_HUMAN | VLSEAAISASLEK.light | FALSE | 659.37 | Unit | 1105.57 | Unit | 10 | 130 | 21.4 | 4 | Positive |
| sp\|O95573\|ACSL3_HUMAN | VLSEAAISASLEK.light | FALSE | 659.37 | Unit | 889.50 | Unit | 10 | 130 | 21.4 | 4 | Positive |
| sp\|O95573\|ACSL3_HUMAN | VLSEAAISASLEK.light | FALSE | 659.37 | Unit | 818.46 | Unit | 10 | 130 | 21.4 | 4 | Positive |
| sp\|O95573\|ACSL3_HUMAN | VLSEAAISASLEK.light | FALSE | 659.37 | Unit | 634.34 | Unit | 10 | 130 | 21.4 | 4 | Positive |
| sp\|O60313\|OPA1_HUMAN | IDQLQEELLHTQLK.light | FALSE | 854.47 | Unit | 1110.62 | Unit | 10 | 130 | 27.5 | 4 | Positive |
| sp\|O60313\|OPA1_HUMAN | IDQLQEELLHTQLK.light | FALSE | 854.47 | Unit | 626.36 | Unit | 10 | 130 | 27.5 | 4 | Positive |
| sp\|O60313\|OPA1_HUMAN | IDQLQEELLHTQLK.light | FALSE | 854.47 | Unit | 260.20 | Unit | 10 | 130 | 27.5 | 4 | Positive |
| sp\|O60313\|OPA1_HUMAN | IDQLQEELLHTQLK.light | FALSE | 854.47 | Unit | 147.11 | Unit | 10 | 130 | 27.5 | 4 | Positive |
| sp\|O60313\|OPA1_HUMAN | LDAFIEALHQEK.light | FALSE | 707.37 | Unit | 1114.59 | Unit | 10 | 130 | 22.9 | 4 | Positive |
| sp\|O60313\|OPA1_HUMAN | LDAFIEALHQEK.light | FALSE | 707.37 | Unit | 967.52 | Unit | 10 | 130 | 22.9 | 4 | Positive |
| sp\|O60313\|OPA1_HUMAN | LDAFIEALHQEK.light | FALSE | 707.37 | Unit | 854.44 | Unit | 10 | 130 | 22.9 | 4 | Positive |
| sp\|O60313\|OPA1_HUMAN | LDAFIEALHQEK.light | FALSE | 707.37 | Unit | 541.27 | Unit | 10 | 130 | 22.9 | 4 | Positive |
| sp\|P12956\|XRCC6_HUMAN | DSLIFLVDASK.light | FALSE | 604.33 | Unit | 892.51 | Unit | 10 | 130 | 19.7 | 4 | Positive |
| sp\|P12956\|XRCC6_HUMAN | DSLIFLVDASK.light | FALSE | 604.33 | Unit | 779.43 | Unit | 10 | 130 | 19.7 | 4 | Positive |
| sp\|P12956\|XRCC6_HUMAN | DSLIFLVDASK.light | FALSE | 604.33 | Unit | 632.36 | Unit | 10 | 130 | 19.7 | 4 | Positive |
| sp\|P12956\|XRCC6_HUMAN | DSLIFLVDASK.light | FALSE | 604.33 | Unit | 519.28 | Unit | 10 | 130 | 19.7 | 4 | Positive |
| sp\|P12956\|XRCC6_HUMAN | VEYSEEELK.light | FALSE | 563.27 | Unit | 1026.46 | Unit | 10 | 130 | 18.5 | 4 | Positive |
| sp\|P12956\|XRCC6_HUMAN | VEYSEEELK.light | FALSE | 563.27 | Unit | 897.42 | Unit | 10 | 130 | 18.5 | 4 | Positive |
| sp\|P12956\|XRCC6_HUMAN | VEYSEEELK.light | FALSE | 563.27 | Unit | 734.36 | Unit | 10 | 130 | 18.5 | 4 | Positive |
| sp\|P12956\|XRCC6_HUMAN | VEYSEEELK.light | FALSE | 563.27 | Unit | 147.11 | Unit | 10 | 130 | 18.5 | 4 | Positive |
| sp\|Q16762\|THTR_HUMAN | TYEQVLENLESK.light | FALSE | 726.86 | Unit | 931.51 | Unit | 10 | 130 | 23.5 | 4 | Positive |
| sp\|Q16762\|THTR_HUMAN | TYEQVLENLESK.light | FALSE | 726.86 | Unit | 832.44 | Unit | 10 | 130 | 23.5 | 4 | Positive |
| sp\|Q16762\|THTR_HUMAN | TYEQVLENLESK.light | FALSE | 726.86 | Unit | 719.36 | Unit | 10 | 130 | 23.5 | 4 | Positive |
| sp\|Q16762\|THTR_HUMAN | TYEQVLENLESK.light | FALSE | 726.86 | Unit | 147.11 | Unit | 10 | 130 | 23.5 | 4 | Positive |
| sp\|Q16762\|THTR_HUMAN | FLGTEPEPDAVGLDSGHIR.light | FALSE | 1005.50 | Unit | 1024.55 | Unit | 10 | 130 | 32.2 | 4 | Positive |
| sp\|Q16762\|THTR_HUMAN | FLGTEPEPDAVGLDSGHIR.light | FALSE | 1005.50 | Unit | 854.45 | Unit | 10 | 130 | 32.2 | 4 | Positive |
| sp\|Q16762\|THTR_HUMAN | FLGTEPEPDAVGLDSGHIR.light | FALSE | 1005.50 | Unit | 569.32 | Unit | 10 | 130 | 32.2 | 4 | Positive |
| sp\|Q16762\|THTR_HUMAN | FLGTEPEPDAVGLDSGHIR.light | FALSE | 1005.50 | Unit | 175.12 | Unit | 10 | 130 | 32.2 | 4 | Positive |
| sp\|Q16222\|UAP1_HUMAN | VAVLLLAGGQGTR.light | FALSE | 627.88 | Unit | 872.49 | Unit | 10 | 130 | 20.5 | 4 | Positive |
| sp\|Q16222\|UAP1_HUMAN | VAVLLLAGGQGTR.light | FALSE | 627.88 | Unit | 759.41 | Unit | 10 | 130 | 20.5 | 4 | Positive |
| sp\|Q16222\|UAP1_HUMAN | VAVLLLAGGQGTR.light | FALSE | 627.88 | Unit | 646.33 | Unit | 10 | 130 | 20.5 | 4 | Positive |
| sp\|Q16222\|UAP1_HUMAN | VAVLLLAGGQGTR.light | FALSE | 627.88 | Unit | 575.29 | Unit | 10 | 130 | 20.5 | 4 | Positive |
| sp\|Q16222\|UAP1_HUMAN | ALAAQNIVEDMEQR.light | FALSE | 794.39 | Unit | 1133.53 | Unit | 10 | 130 | 25.6 | 4 | Positive |
| sp\|Q16222\|UAP1_HUMAN | ALAAQNIVEDMEQR.light | FALSE | 794.39 | Unit | 1019.48 | Unit | 10 | 130 | 25.6 | 4 | Positive |
| sp\|Q16222\|UAP1_HUMAN | ALAAQNIVEDMEQR.light | FALSE | 794.39 | Unit | 906.40 | Unit | 10 | 130 | 25.6 | 4 | Positive |
| sp\|Q16222\|UAP1_HUMAN | ALAAQNIVEDMEQR.light | FALSE | 794.39 | Unit | 807.33 | Unit | 10 | 130 | 25.6 | 4 | Positive |
| sp\|Q14739\|LBR_HUMAN | TFEVTPIR.light | FALSE | 481.77 | Unit | 861.48 | Unit | 10 | 130 | 15.9 | 4 | Positive |
| sp\|Q8WVV9\|HNRLL_HUMAN | SFSQPEAGGSHHK.light | FALSE | 684.82 | Unit | 919.44 | Unit | 10 | 130 | 22.2 | 4 | Positive |
| sp\|Q8WVV9\|HNRLL_HUMAN | SFSQPEAGGSHHK.light | FALSE | 684.82 | Unit | 693.34 | Unit | 10 | 130 | 22.2 | 4 | Positive |
| sp\|Q8WVV9\|HNRLL_HUMAN | SFSQPEAGGSHHK.light | FALSE | 684.82 | Unit | 284.17 | Unit | 10 | 130 | 22.2 | 4 | Positive |
| sp\|Q8WVV9\|HNRLL_HUMAN | SFSQPEAGGSHHK.light | FALSE | 684.82 | Unit | 147.11 | Unit | 10 | 130 | 22.2 | 4 | Positive |
| sp\|Q8WVV9\|HNRLL_HUMAN | VFNLFC[+57.0]LYGNIEK.light | FALSE | 808.91 | Unit | 1143.55 | Unit | 10 | 130 | 26.1 | 4 | Positive |
| sp\|Q8WVV9\|HNRLL_HUMAN | VFNLFC[+57.0]LYGNIEK.light | FALSE | 808.91 | Unit | 996.48 | Unit | 10 | 130 | 26.1 | 4 | Positive |
| sp\|Q8WVV9\|HNRLL_HUMAN | VFNLFC[+57.0]LYGNIEK.light | FALSE | 808.91 | Unit | 723.37 | Unit | 10 | 130 | 26.1 | 4 | Positive |
| sp\|Q8WVV9\|HNRLL_HUMAN | VFNLFC[+57.0]LYGNIEK.light | FALSE | 808.91 | Unit | 147.11 | Unit | 10 | 130 | 26.1 | 4 | Positive |
| sp\|P21333\|FLNA_HUMAN | ANLPQSFQVDTSK.light | FALSE | 717.86 | Unit | 1136.56 | Unit | 10 | 130 | 23.3 | 4 | Positive |
| sp\|P21333\|FLNA_HUMAN | ANLPQSFQVDTSK.light | FALSE | 717.86 | Unit | 911.45 | Unit | 10 | 130 | 23.3 | 4 | Positive |
| sp\|P21333\|FLNA_HUMAN | ANLPQSFQVDTSK.light | FALSE | 717.86 | Unit | 824.41 | Unit | 10 | 130 | 23.3 | 4 | Positive |
| sp\|P21333\|FLNA_HUMAN | ANLPQSFQVDTSK.light | FALSE | 717.86 | Unit | 450.22 | Unit | 10 | 130 | 23.3 | 4 | Positive |
| sp\|P21333\|FLNA_HUMAN | DAGEGLLAVQITDPEGKPK.light | FALSE | 969.51 | Unit | 1112.59 | Unit | 10 | 130 | 31.1 | 4 | Positive |
| sp\|P21333\|FLNA_HUMAN | DAGEGLLAVQITDPEGKPK.light | FALSE | 969.51 | Unit | 871.45 | Unit | 10 | 130 | 31.1 | 4 | Positive |
| sp\|P21333\|FLNA_HUMAN | DAGEGLLAVQITDPEGKPK.light | FALSE | 969.51 | Unit | 655.38 | Unit | 10 | 130 | 31.1 | 4 | Positive |
| sp\|P21333\|FLNA_HUMAN | DAGEGLLAVQITDPEGKPK.light | FALSE | 969.51 | Unit | 244.17 | Unit | 10 | 130 | 31.1 | 4 | Positive |
| sp\|Q9H2U2\|IPYR2_HUMAN | LIAINANDPEASK.light | FALSE | 678.36 | Unit | 1129.55 | Unit | 10 | 130 | 22 | 4 | Positive |
| sp\|Q9H2U2\|IPYR2_HUMAN | LIAINANDPEASK.light | FALSE | 678.36 | Unit | 945.43 | Unit | 10 | 130 | 22 | 4 | Positive |
| sp\|Q9H2U2\|IPYR2_HUMAN | LIAINANDPEASK.light | FALSE | 678.36 | Unit | 760.35 | Unit | 10 | 130 | 22 | 4 | Positive |
| sp\|Q9H2U2\|IPYR2_HUMAN | LIAINANDPEASK.light | FALSE | 678.36 | Unit | 531.28 | Unit | 10 | 130 | 22 | 4 | Positive |
| sp\|Q9H2U2\|IPYR2_HUMAN | SLVESVSSSPNK.light | FALSE | 617.32 | Unit | 934.45 | Unit | 10 | 130 | 20.1 | 4 | Positive |
| sp\|Q9H2U2\|IPYR2_HUMAN | SLVESVSSSPNK.light | FALSE | 617.32 | Unit | 805.41 | Unit | 10 | 130 | 20.1 | 4 | Positive |
| sp\|Q9H2U2\|IPYR2_HUMAN | SLVESVSSSPNK.light | FALSE | 617.32 | Unit | 619.30 | Unit | 10 | 130 | 20.1 | 4 | Positive |
| sp\|Q9H2U2\|IPYR2_HUMAN | SLVESVSSSPNK.light | FALSE | 617.32 | Unit | 358.21 | Unit | 10 | 130 | 20.1 | 4 | Positive |
| sp\|P17858\|PFKAL_HUMAN | IMEVIDAITTTAQSHQR.light | FALSE | 957.49 | Unit | 1142.59 | Unit | 10 | 130 | 30.7 | 4 | Positive |
| sp\|P17858\|PFKAL_HUMAN | IMEVIDAITTTAQSHQR.light | FALSE | 957.49 | Unit | 1029.51 | Unit | 10 | 130 | 30.7 | 4 | Positive |
| sp\|P17858\|PFKAL_HUMAN | IMEVIDAITTTAQSHQR.light | FALSE | 957.49 | Unit | 928.46 | Unit | 10 | 130 | 30.7 | 4 | Positive |
| sp\|P17858\|PFKAL_HUMAN | IMEVIDAITTTAQSHQR.light | FALSE | 957.49 | Unit | 527.27 | Unit | 10 | 130 | 30.7 | 4 | Positive |
| sp\|P17858\|PFKAL_HUMAN | GQVQEVGWHDVAGWLGR.light | FALSE | 947.47 | Unit | 1010.52 | Unit | 10 | 130 | 30.4 | 4 | Positive |
| sp\|P17858\|PFKAL_HUMAN | GQVQEVGWHDVAGWLGR.light | FALSE | 947.47 | Unit | 873.46 | Unit | 10 | 130 | 30.4 | 4 | Positive |
| sp\|P17858\|PFKAL_HUMAN | GQVQEVGWHDVAGWLGR.light | FALSE | 947.47 | Unit | 758.43 | Unit | 10 | 130 | 30.4 | 4 | Positive |
| sp\|P17858\|PFKAL_HUMAN | GQVQEVGWHDVAGWLGR.light | FALSE | 947.47 | Unit | 175.12 | Unit | 10 | 130 | 30.4 | 4 | Positive |
| sp\|P08473\|NEP_HUMAN | ALYGTTSETATWR.light | FALSE | 728.86 | Unit | 1052.50 | Unit | 10 | 130 | 23.6 | 4 | Positive |
| sp\|P08473\|NEP_HUMAN | ALYGTTSETATWR.light | FALSE | 728.86 | Unit | 951.45 | Unit | 10 | 130 | 23.6 | 4 | Positive |
| sp\|P08473\|NEP_HUMAN | ALYGTTSETATWR.light | FALSE | 728.86 | Unit | 634.33 | Unit | 10 | 130 | 23.6 | 4 | Positive |
| sp\|P08473\|NEP_HUMAN | ALYGTTSETATWR.light | FALSE | 728.86 | Unit | 462.25 | Unit | 10 | 130 | 23.6 | 4 | Positive |
| sp\|P08473\|NEP_HUMAN | DGDLVDWWTQQSASNFK.light | FALSE | 998.96 | Unit | 1196.57 | Unit | 10 | 130 | 32 | 4 | Positive |
| sp\|P08473\|NEP_HUMAN | DGDLVDWWTQQSASNFK.light | FALSE | 998.96 | Unit | 1010.49 | Unit | 10 | 130 | 32 | 4 | Positive |
| sp\|P08473\|NEP_HUMAN | DGDLVDWWTQQSASNFK.light | FALSE | 998.96 | Unit | 653.33 | Unit | 10 | 130 | 32 | 4 | Positive |
| sp\|P08473\|NEP_HUMAN | DGDLVDWWTQQSASNFK.light | FALSE | 998.96 | Unit | 495.26 | Unit | 10 | 130 | 32 | 4 | Positive |
| sp\|Q6PKG0\|LARP1_HUMAN | ENGFTQHVYHK.light | FALSE | 680.33 | Unit | 1116.56 | Unit | 10 | 130 | 22.1 | 4 | Positive |
| sp\|Q6PKG0\|LARP1_HUMAN | ENGFTQHVYHK.light | FALSE | 680.33 | Unit | 447.24 | Unit | 10 | 130 | 22.1 | 4 | Positive |
| sp\|Q6PKG0\|LARP1_HUMAN | ENGFTQHVYHK.light | FALSE | 680.33 | Unit | 284.17 | Unit | 10 | 130 | 22.1 | 4 | Positive |
| sp\|Q6PKG0\|LARP1_HUMAN | ENGFTQHVYHK.light | FALSE | 680.33 | Unit | 147.11 | Unit | 10 | 130 | 22.1 | 4 | Positive |
| sp\|Q6PKG0\|LARP1_HUMAN | DYEAGQLYGLEK.light | FALSE | 693.33 | Unit | 978.53 | Unit | 10 | 130 | 22.5 | 4 | Positive |
| sp\|Q6PKG0\|LARP1_HUMAN | DYEAGQLYGLEK.light | FALSE | 693.33 | Unit | 907.49 | Unit | 10 | 130 | 22.5 | 4 | Positive |
| sp\|Q6PKG0\|LARP1_HUMAN | DYEAGQLYGLEK.light | FALSE | 693.33 | Unit | 609.32 | Unit | 10 | 130 | 22.5 | 4 | Positive |
| sp\|Q6PKG0\|LARP1_HUMAN | DYEAGQLYGLEK.light | FALSE | 693.33 | Unit | 446.26 | Unit | 10 | 130 | 22.5 | 4 | Positive |
| sp\|Q9Y678\|COPG1_HUMAN | GLGPLFK.light | FALSE | 366.23 | Unit | 561.34 | Unit | 10 | 130 | 12.4 | 4 | Positive |
| sp\|Q9Y678\|COPG1_HUMAN | GLGPLFK.light | FALSE | 366.23 | Unit | 504.32 | Unit | 10 | 130 | 12.4 | 4 | Positive |
| sp\|Q9Y678\|COPG1_HUMAN | GLGPLFK.light | FALSE | 366.23 | Unit | 294.18 | Unit | 10 | 130 | 12.4 | 4 | Positive |
| sp\|Q9Y678\|COPG1_HUMAN | GLGPLFK.light | FALSE | 366.23 | Unit | 147.11 | Unit | 10 | 130 | 12.4 | 4 | Positive |
| sp\|Q9Y678\|COPG1_HUMAN | NTHTLLLAGVFR.light | FALSE | 671.39 | Unit | 1126.67 | Unit | 10 | 130 | 21.8 | 4 | Positive |
| sp\|Q9Y678\|COPG1_HUMAN | NTHTLLLAGVFR.light | FALSE | 671.39 | Unit | 989.61 | Unit | 10 | 130 | 21.8 | 4 | Positive |
| sp\|Q9Y678\|COPG1_HUMAN | NTHTLLLAGVFR.light | FALSE | 671.39 | Unit | 888.57 | Unit | 10 | 130 | 21.8 | 4 | Positive |
| sp\|Q9Y678\|COPG1_HUMAN | NTHTLLLAGVFR.light | FALSE | 671.39 | Unit | 775.48 | Unit | 10 | 130 | 21.8 | 4 | Positive |
| sp\|P33121\|ACSL1_HUMAN | GIQVSNNGPC[+57.0]LGSR.light | FALSE | 729.86 | Unit | 1160.55 | Unit | 10 | 130 | 23.6 | 4 | Positive |
| sp\|P33121\|ACSL1_HUMAN | GIQVSNNGPC[+57.0]LGSR.light | FALSE | 729.86 | Unit | 1061.48 | Unit | 10 | 130 | 23.6 | 4 | Positive |
| sp\|P33121\|ACSL1_HUMAN | GIQVSNNGPC[+57.0]LGSR.light | FALSE | 729.86 | Unit | 746.36 | Unit | 10 | 130 | 23.6 | 4 | Positive |
| sp\|P33121\|ACSL1_HUMAN | GIQVSNNGPC[+57.0]LGSR.light | FALSE | 729.86 | Unit | 319.17 | Unit | 10 | 130 | 23.6 | 4 | Positive |
| sp\|P33121\|ACSL1_HUMAN | AELSLVFVDKPEK.light | FALSE | 737.91 | Unit | 1161.65 | Unit | 10 | 130 | 23.9 | 4 | Positive |
| sp\|P33121\|ACSL1_HUMAN | AELSLVFVDKPEK.light | FALSE | 737.91 | Unit | 961.54 | Unit | 10 | 130 | 23.9 | 4 | Positive |
| sp\|P33121\|ACSL1_HUMAN | AELSLVFVDKPEK.light | FALSE | 737.91 | Unit | 862.47 | Unit | 10 | 130 | 23.9 | 4 | Positive |
| sp\|P33121\|ACSL1_HUMAN | AELSLVFVDKPEK.light | FALSE | 737.91 | Unit | 373.21 | Unit | 10 | 130 | 23.9 | 4 | Positive |
| sp\|O75607\|NPM3_HUMAN | NHDHQEIAVPVANLK.light | FALSE | 842.94 | Unit | 641.40 | Unit | 10 | 130 | 27.1 | 4 | Positive |
| sp\|O75607\|NPM3_HUMAN | NHDHQEIAVPVANLK.light | FALSE | 842.94 | Unit | 445.28 | Unit | 10 | 130 | 27.1 | 4 | Positive |
| sp\|O75607\|NPM3_HUMAN | NHDHQEIAVPVANLK.light | FALSE | 842.94 | Unit | 374.24 | Unit | 10 | 130 | 27.1 | 4 | Positive |
| sp\|O75607\|NPM3_HUMAN | NHDHQEIAVPVANLK.light | FALSE | 842.94 | Unit | 147.11 | Unit | 10 | 130 | 27.1 | 4 | Positive |
| sp\|P55060\|XPO2_HUMAN | LLQTDDEEEAGLLELLK.light | FALSE | 965.01 | Unit | 1114.64 | Unit | 10 | 130 | 30.9 | 4 | Positive |
| sp\|P55060\|XPO2_HUMAN | LLQTDDEEEAGLLELLK.light | FALSE | 965.01 | Unit | 985.59 | Unit | 10 | 130 | 30.9 | 4 | Positive |
| sp\|P55060\|XPO2_HUMAN | LLQTDDEEEAGLLELLK.light | FALSE | 965.01 | Unit | 856.55 | Unit | 10 | 130 | 30.9 | 4 | Positive |
| sp\|P55060\|XPO2_HUMAN | LLQTDDEEEAGLLELLK.light | FALSE | 965.01 | Unit | 785.51 | Unit | 10 | 130 | 30.9 | 4 | Positive |
| sp\|P55060\|XPO2_HUMAN | NLFEDQNTLTSIC[+57.0]EK.light | FALSE | 906.43 | Unit | 951.48 | Unit | 10 | 130 | 29.1 | 4 | Positive |
| sp\|P55060\|XPO2_HUMAN | NLFEDQNTLTSIC[+57.0]EK.light | FALSE | 906.43 | Unit | 737.35 | Unit | 10 | 130 | 29.1 | 4 | Positive |
| sp\|P55060\|XPO2_HUMAN | NLFEDQNTLTSIC[+57.0]EK.light | FALSE | 906.43 | Unit | 636.30 | Unit | 10 | 130 | 29.1 | 4 | Positive |
| sp\|P55060\|XPO2_HUMAN | NLFEDQNTLTSIC[+57.0]EK.light | FALSE | 906.43 | Unit | 436.19 | Unit | 10 | 130 | 29.1 | 4 | Positive |
| sp\|Q53H82\|LACB2_HUMAN | SINNDTTYC[+57.0]IK.light | FALSE | 664.81 | Unit | 1128.50 | Unit | 10 | 130 | 21.6 | 4 | Positive |
| sp\|Q53H82\|LACB2_HUMAN | SINNDTTYC[+57.0]IK.light | FALSE | 664.81 | Unit | 785.39 | Unit | 10 | 130 | 21.6 | 4 | Positive |
| sp\|Q53H82\|LACB2_HUMAN | SINNDTTYC[+57.0]IK.light | FALSE | 664.81 | Unit | 420.23 | Unit | 10 | 130 | 21.6 | 4 | Positive |
| sp\|Q53H82\|LACB2_HUMAN | SINNDTTYC[+57.0]IK.light | FALSE | 664.81 | Unit | 147.11 | Unit | 10 | 130 | 21.6 | 4 | Positive |
| sp\|Q53H82\|LACB2_HUMAN | SFTVMELVK.light | FALSE | 527.29 | Unit | 819.46 | Unit | 10 | 130 | 17.3 | 4 | Positive |
| sp\|Q53H82\|LACB2_HUMAN | SFTVMELVK.light | FALSE | 527.29 | Unit | 718.42 | Unit | 10 | 130 | 17.3 | 4 | Positive |
| sp\|Q53H82\|LACB2_HUMAN | SFTVMELVK.light | FALSE | 527.29 | Unit | 619.35 | Unit | 10 | 130 | 17.3 | 4 | Positive |
| sp\|Q53H82\|LACB2_HUMAN | SFTVMELVK.light | FALSE | 527.29 | Unit | 147.11 | Unit | 10 | 130 | 17.3 | 4 | Positive |
| sp\|Q6DD88\|ATLA3_HUMAN | DQHSFELDEK.light | FALSE | 624.28 | Unit | 1004.47 | Unit | 10 | 130 | 20.4 | 4 | Positive |
| sp\|Q6DD88\|ATLA3_HUMAN | DQHSFELDEK.light | FALSE | 624.28 | Unit | 867.41 | Unit | 10 | 130 | 20.4 | 4 | Positive |
| sp\|Q6DD88\|ATLA3_HUMAN | DQHSFELDEK.light | FALSE | 624.28 | Unit | 276.16 | Unit | 10 | 130 | 20.4 | 4 | Positive |
| sp\|Q6DD88\|ATLA3_HUMAN | DQHSFELDEK.light | FALSE | 624.28 | Unit | 147.11 | Unit | 10 | 130 | 20.4 | 4 | Positive |
| sp\|Q6DD88\|ATLA3_HUMAN | EQLQALIPYVLNPSK.light | FALSE | 856.98 | Unit | 1143.68 | Unit | 10 | 130 | 27.6 | 4 | Positive |
| sp\|Q6DD88\|ATLA3_HUMAN | EQLQALIPYVLNPSK.light | FALSE | 856.98 | Unit | 1030.59 | Unit | 10 | 130 | 27.6 | 4 | Positive |
| sp\|Q6DD88\|ATLA3_HUMAN | EQLQALIPYVLNPSK.light | FALSE | 856.98 | Unit | 917.51 | Unit | 10 | 130 | 27.6 | 4 | Positive |
| sp\|Q6DD88\|ATLA3_HUMAN | EQLQALIPYVLNPSK.light | FALSE | 856.98 | Unit | 331.20 | Unit | 10 | 130 | 27.6 | 4 | Positive |
| sp\|P07195\|LDHB_HUMAN | DYSVTANSK.light | FALSE | 492.74 | Unit | 706.37 | Unit | 10 | 130 | 16.3 | 4 | Positive |
| sp\|P07195\|LDHB_HUMAN | DYSVTANSK.light | FALSE | 492.74 | Unit | 520.27 | Unit | 10 | 130 | 16.3 | 4 | Positive |
| sp\|P07195\|LDHB_HUMAN | DYSVTANSK.light | FALSE | 492.74 | Unit | 348.19 | Unit | 10 | 130 | 16.3 | 4 | Positive |
| sp\|P07195\|LDHB_HUMAN | DYSVTANSK.light | FALSE | 492.74 | Unit | 147.11 | Unit | 10 | 130 | 16.3 | 4 | Positive |
| sp\|P07195\|LDHB_HUMAN | FIIPQIVK.light | FALSE | 479.31 | Unit | 810.54 | Unit | 10 | 130 | 15.9 | 4 | Positive |
| sp\|P07195\|LDHB_HUMAN | FIIPQIVK.light | FALSE | 479.31 | Unit | 697.46 | Unit | 10 | 130 | 15.9 | 4 | Positive |
| sp\|P07195\|LDHB_HUMAN | FIIPQIVK.light | FALSE | 479.31 | Unit | 584.38 | Unit | 10 | 130 | 15.9 | 4 | Positive |
| sp\|P07195\|LDHB_HUMAN | FIIPQIVK.light | FALSE | 479.31 | Unit | 147.11 | Unit | 10 | 130 | 15.9 | 4 | Positive |
| sp\|Q13228\|SBP1_HUMAN | LVLPSLISSR.light | FALSE | 542.84 | Unit | 872.52 | Unit | 10 | 130 | 17.8 | 4 | Positive |
| sp\|Q13228\|SBP1_HUMAN | LVLPSLISSR.light | FALSE | 542.84 | Unit | 759.44 | Unit | 10 | 130 | 17.8 | 4 | Positive |
| sp\|Q13228\|SBP1_HUMAN | LVLPSLISSR.light | FALSE | 542.84 | Unit | 662.38 | Unit | 10 | 130 | 17.8 | 4 | Positive |
| sp\|Q13228\|SBP1_HUMAN | LVLPSLISSR.light | FALSE | 542.84 | Unit | 462.27 | Unit | 10 | 130 | 17.8 | 4 | Positive |
| sp\|Q13228\|SBP1_HUMAN | GGPVQVLEDEELK.light | FALSE | 706.87 | Unit | 1102.56 | Unit | 10 | 130 | 22.9 | 4 | Positive |
| sp\|Q13228\|SBP1_HUMAN | GGPVQVLEDEELK.light | FALSE | 706.87 | Unit | 974.50 | Unit | 10 | 130 | 22.9 | 4 | Positive |
| sp\|Q13228\|SBP1_HUMAN | GGPVQVLEDEELK.light | FALSE | 706.87 | Unit | 875.44 | Unit | 10 | 130 | 22.9 | 4 | Positive |
| sp\|Q13228\|SBP1_HUMAN | GGPVQVLEDEELK.light | FALSE | 706.87 | Unit | 633.31 | Unit | 10 | 130 | 22.9 | 4 | Positive |
| sp\|Q9UBQ7\|GRHPR_HUMAN | AADC[+57.0]EVEQWDSDEPIPAK.light | FALSE | 1030.45 | Unit | 971.47 | Unit | 10 | 130 | 32.9 | 4 | Positive |
| sp\|Q9UBQ7\|GRHPR_HUMAN | AADC[+57.0]EVEQWDSDEPIPAK.light | FALSE | 1030.45 | Unit | 525.34 | Unit | 10 | 130 | 32.9 | 4 | Positive |
| sp\|Q9UBQ7\|GRHPR_HUMAN | AADC[+57.0]EVEQWDSDEPIPAK.light | FALSE | 1030.45 | Unit | 315.20 | Unit | 10 | 130 | 32.9 | 4 | Positive |
| sp\|Q9UBQ7\|GRHPR_HUMAN | AADC[+57.0]EVEQWDSDEPIPAK.light | FALSE | 1030.45 | Unit | 147.11 | Unit | 10 | 130 | 32.9 | 4 | Positive |
| sp\|Q13813\|SPTN1_HUMAN | DLASVNNLLK.light | FALSE | 543.81 | Unit | 858.50 | Unit | 10 | 130 | 17.9 | 4 | Positive |
| sp\|Q13813\|SPTN1_HUMAN | DLASVNNLLK.light | FALSE | 543.81 | Unit | 787.47 | Unit | 10 | 130 | 17.9 | 4 | Positive |
| sp\|Q13813\|SPTN1_HUMAN | DLASVNNLLK.light | FALSE | 543.81 | Unit | 700.44 | Unit | 10 | 130 | 17.9 | 4 | Positive |
| sp\|Q13813\|SPTN1_HUMAN | DLASVNNLLK.light | FALSE | 543.81 | Unit | 601.37 | Unit | 10 | 130 | 17.9 | 4 | Positive |
| sp\|Q13813\|SPTN1_HUMAN | DLSSVQTLLTK.light | FALSE | 602.84 | Unit | 976.57 | Unit | 10 | 130 | 19.7 | 4 | Positive |
| sp\|Q13813\|SPTN1_HUMAN | DLSSVQTLLTK.light | FALSE | 602.84 | Unit | 889.54 | Unit | 10 | 130 | 19.7 | 4 | Positive |
| sp\|Q13813\|SPTN1_HUMAN | DLSSVQTLLTK.light | FALSE | 602.84 | Unit | 703.43 | Unit | 10 | 130 | 19.7 | 4 | Positive |
| sp\|Q13813\|SPTN1_HUMAN | DLSSVQTLLTK.light | FALSE | 602.84 | Unit | 248.16 | Unit | 10 | 130 | 19.7 | 4 | Positive |
| sp\|Q1KMD3\|HNRL2_HUMAN | FPTLWSGAR.light | FALSE | 517.77 | Unit | 790.42 | Unit | 10 | 130 | 17.1 | 4 | Positive |
| sp\|Q1KMD3\|HNRL2_HUMAN | FPTLWSGAR.light | FALSE | 517.77 | Unit | 689.37 | Unit | 10 | 130 | 17.1 | 4 | Positive |
| sp\|Q1KMD3\|HNRL2_HUMAN | FPTLWSGAR.light | FALSE | 517.77 | Unit | 576.29 | Unit | 10 | 130 | 17.1 | 4 | Positive |
| sp\|Q1KMD3\|HNRL2_HUMAN | FPTLWSGAR.light | FALSE | 517.77 | Unit | 390.21 | Unit | 10 | 130 | 17.1 | 4 | Positive |
| sp\|Q1KMD3\|HNRL2_HUMAN | NYYGYQGYR.light | FALSE | 592.26 | Unit | 906.41 | Unit | 10 | 130 | 19.4 | 4 | Positive |
| sp\|Q1KMD3\|HNRL2_HUMAN | NYYGYQGYR.light | FALSE | 592.26 | Unit | 743.35 | Unit | 10 | 130 | 19.4 | 4 | Positive |
| sp\|Q1KMD3\|HNRL2_HUMAN | NYYGYQGYR.light | FALSE | 592.26 | Unit | 686.33 | Unit | 10 | 130 | 19.4 | 4 | Positive |
| sp\|Q1KMD3\|HNRL2_HUMAN | NYYGYQGYR.light | FALSE | 592.26 | Unit | 175.12 | Unit | 10 | 130 | 19.4 | 4 | Positive |
| sp\|O15020\|SPTN2_HUMAN | DLTSVNILLK.light | FALSE | 558.34 | Unit | 887.56 | Unit | 10 | 130 | 18.3 | 4 | Positive |
| sp\|O15020\|SPTN2_HUMAN | DLTSVNILLK.light | FALSE | 558.34 | Unit | 786.51 | Unit | 10 | 130 | 18.3 | 4 | Positive |
| sp\|O15020\|SPTN2_HUMAN | DLTSVNILLK.light | FALSE | 558.34 | Unit | 699.48 | Unit | 10 | 130 | 18.3 | 4 | Positive |
| sp\|O15020\|SPTN2_HUMAN | DLTSVNILLK.light | FALSE | 558.34 | Unit | 600.41 | Unit | 10 | 130 | 18.3 | 4 | Positive |
| sp\|O15020\|SPTN2_HUMAN | AASAGVPYHGEVPVSLAR.light | FALSE | 890.97 | Unit | 1064.58 | Unit | 10 | 130 | 28.6 | 4 | Positive |
| sp\|O15020\|SPTN2_HUMAN | AASAGVPYHGEVPVSLAR.light | FALSE | 890.97 | Unit | 927.53 | Unit | 10 | 130 | 28.6 | 4 | Positive |
| sp\|O15020\|SPTN2_HUMAN | AASAGVPYHGEVPVSLAR.light | FALSE | 890.97 | Unit | 642.39 | Unit | 10 | 130 | 28.6 | 4 | Positive |
| sp\|O15020\|SPTN2_HUMAN | AASAGVPYHGEVPVSLAR.light | FALSE | 890.97 | Unit | 175.12 | Unit | 10 | 130 | 28.6 | 4 | Positive |
| sp\|Q6NVY1\|HIBCH_HUMAN | FLNALTLNMIR.light | FALSE | 653.37 | Unit | 1045.58 | Unit | 10 | 130 | 21.3 | 4 | Positive |
| sp\|Q6NVY1\|HIBCH_HUMAN | FLNALTLNMIR.light | FALSE | 653.37 | Unit | 931.54 | Unit | 10 | 130 | 21.3 | 4 | Positive |
| sp\|Q6NVY1\|HIBCH_HUMAN | FLNALTLNMIR.light | FALSE | 653.37 | Unit | 860.50 | Unit | 10 | 130 | 21.3 | 4 | Positive |
| sp\|Q6NVY1\|HIBCH_HUMAN | FLNALTLNMIR.light | FALSE | 653.37 | Unit | 747.42 | Unit | 10 | 130 | 21.3 | 4 | Positive |
| sp\|Q6NVY1\|HIBCH_HUMAN | IAPVFFR.light | FALSE | 425.25 | Unit | 665.38 | Unit | 10 | 130 | 14.2 | 4 | Positive |
| sp\|Q6NVY1\|HIBCH_HUMAN | IAPVFFR.light | FALSE | 425.25 | Unit | 568.32 | Unit | 10 | 130 | 14.2 | 4 | Positive |
| sp\|Q6NVY1\|HIBCH_HUMAN | IAPVFFR.light | FALSE | 425.25 | Unit | 469.26 | Unit | 10 | 130 | 14.2 | 4 | Positive |
| sp\|Q6NVY1\|HIBCH_HUMAN | IAPVFFR.light | FALSE | 425.25 | Unit | 175.12 | Unit | 10 | 130 | 14.2 | 4 | Positive |
| sp\|P49915\|GUAA_HUMAN | SGNIVAGIANESK.light | FALSE | 630.33 | Unit | 888.48 | Unit | 10 | 130 | 20.5 | 4 | Positive |
| sp\|P49915\|GUAA_HUMAN | SGNIVAGIANESK.light | FALSE | 630.33 | Unit | 789.41 | Unit | 10 | 130 | 20.5 | 4 | Positive |
| sp\|P49915\|GUAA_HUMAN | SGNIVAGIANESK.light | FALSE | 630.33 | Unit | 718.37 | Unit | 10 | 130 | 20.5 | 4 | Positive |
| sp\|P49915\|GUAA_HUMAN | SGNIVAGIANESK.light | FALSE | 630.33 | Unit | 548.27 | Unit | 10 | 130 | 20.5 | 4 | Positive |
| sp\|P49915\|GUAA_HUMAN | IMYDLTSKPPGTTEWE.light | FALSE | 934.44 | Unit | 1131.53 | Unit | 10 | 130 | 30 | 4 | Positive |
| sp\|P49915\|GUAA_HUMAN | IMYDLTSKPPGTTEWE.light | FALSE | 934.44 | Unit | 1044.50 | Unit | 10 | 130 | 30 | 4 | Positive |
| sp\|P49915\|GUAA_HUMAN | IMYDLTSKPPGTTEWE.light | FALSE | 934.44 | Unit | 916.40 | Unit | 10 | 130 | 30 | 4 | Positive |
| sp\|P49915\|GUAA_HUMAN | IMYDLTSKPPGTTEWE.light | FALSE | 934.44 | Unit | 334.14 | Unit | 10 | 130 | 30 | 4 | Positive |
| sp\|Q96HC4\|PDLI5_HUMAN | ILAQITGTEHLK.light | FALSE | 662.39 | Unit | 1097.59 | Unit | 10 | 130 | 21.5 | 4 | Positive |
| sp\|Q96HC4\|PDLI5_HUMAN | ILAQITGTEHLK.light | FALSE | 662.39 | Unit | 898.50 | Unit | 10 | 130 | 21.5 | 4 | Positive |
| sp\|Q96HC4\|PDLI5_HUMAN | ILAQITGTEHLK.light | FALSE | 662.39 | Unit | 785.42 | Unit | 10 | 130 | 21.5 | 4 | Positive |
| sp\|Q96HC4\|PDLI5_HUMAN | ILAQITGTEHLK.light | FALSE | 662.39 | Unit | 684.37 | Unit | 10 | 130 | 21.5 | 4 | Positive |
| sp\|Q96HC4\|PDLI5_HUMAN | GPFLVALGK.light | FALSE | 451.28 | Unit | 747.48 | Unit | 10 | 130 | 15 | 4 | Positive |
| sp\|Q96HC4\|PDLI5_HUMAN | GPFLVALGK.light | FALSE | 451.28 | Unit | 600.41 | Unit | 10 | 130 | 15 | 4 | Positive |
| sp\|Q96HC4\|PDLI5_HUMAN | GPFLVALGK.light | FALSE | 451.28 | Unit | 487.32 | Unit | 10 | 130 | 15 | 4 | Positive |
| sp\|Q96HC4\|PDLI5_HUMAN | GPFLVALGK.light | FALSE | 451.28 | Unit | 204.13 | Unit | 10 | 130 | 15 | 4 | Positive |
| sp\|P08133\|ANXA6_HUMAN | EAILDIITSR.light | FALSE | 565.82 | Unit | 817.48 | Unit | 10 | 130 | 18.5 | 4 | Positive |
| sp\|P08133\|ANXA6_HUMAN | EAILDIITSR.light | FALSE | 565.82 | Unit | 704.39 | Unit | 10 | 130 | 18.5 | 4 | Positive |
| sp\|P08133\|ANXA6_HUMAN | EAILDIITSR.light | FALSE | 565.82 | Unit | 589.37 | Unit | 10 | 130 | 18.5 | 4 | Positive |
| sp\|P08133\|ANXA6_HUMAN | EAILDIITSR.light | FALSE | 565.82 | Unit | 476.28 | Unit | 10 | 130 | 18.5 | 4 | Positive |
| sp\|P08133\|ANXA6_HUMAN | ALIEILATR.light | FALSE | 500.31 | Unit | 815.50 | Unit | 10 | 130 | 16.5 | 4 | Positive |
| sp\|P08133\|ANXA6_HUMAN | ALIEILATR.light | FALSE | 500.31 | Unit | 702.41 | Unit | 10 | 130 | 16.5 | 4 | Positive |
| sp\|P08133\|ANXA6_HUMAN | ALIEILATR.light | FALSE | 500.31 | Unit | 573.37 | Unit | 10 | 130 | 16.5 | 4 | Positive |
| sp\|P08133\|ANXA6_HUMAN | ALIEILATR.light | FALSE | 500.31 | Unit | 460.29 | Unit | 10 | 130 | 16.5 | 4 | Positive |
| sp\|P35580\|MYH10_HUMAN | HGFEAASIK.light | FALSE | 480.25 | Unit | 822.44 | Unit | 10 | 130 | 15.9 | 4 | Positive |
| sp\|P35580\|MYH10_HUMAN | HGFEAASIK.light | FALSE | 480.25 | Unit | 765.41 | Unit | 10 | 130 | 15.9 | 4 | Positive |
| sp\|P35580\|MYH10_HUMAN | HGFEAASIK.light | FALSE | 480.25 | Unit | 260.20 | Unit | 10 | 130 | 15.9 | 4 | Positive |
| sp\|P35580\|MYH10_HUMAN | HGFEAASIK.light | FALSE | 480.25 | Unit | 147.11 | Unit | 10 | 130 | 15.9 | 4 | Positive |
| sp\|P35580\|MYH10_HUMAN | HATALEELSEQLEQAK.light | FALSE | 898.95 | Unit | 1045.55 | Unit | 10 | 130 | 28.9 | 4 | Positive |
| sp\|P35580\|MYH10_HUMAN | HATALEELSEQLEQAK.light | FALSE | 898.95 | Unit | 932.47 | Unit | 10 | 130 | 28.9 | 4 | Positive |
| sp\|P35580\|MYH10_HUMAN | HATALEELSEQLEQAK.light | FALSE | 898.95 | Unit | 475.25 | Unit | 10 | 130 | 28.9 | 4 | Positive |
| sp\|P35580\|MYH10_HUMAN | HATALEELSEQLEQAK.light | FALSE | 898.95 | Unit | 147.11 | Unit | 10 | 130 | 28.9 | 4 | Positive |
| sp\|P35573\|GDE_HUMAN | HLSLGSVQLC[+57.0]GVGK.light | FALSE | 727.89 | Unit | 1004.52 | Unit | 10 | 130 | 23.6 | 4 | Positive |
| sp\|P35573\|GDE_HUMAN | HLSLGSVQLC[+57.0]GVGK.light | FALSE | 727.89 | Unit | 520.25 | Unit | 10 | 130 | 23.6 | 4 | Positive |
| sp\|P35573\|GDE_HUMAN | HLSLGSVQLC[+57.0]GVGK.light | FALSE | 727.89 | Unit | 204.13 | Unit | 10 | 130 | 23.6 | 4 | Positive |
| sp\|P35573\|GDE_HUMAN | HLSLGSVQLC[+57.0]GVGK.light | FALSE | 727.89 | Unit | 147.11 | Unit | 10 | 130 | 23.6 | 4 | Positive |
| sp\|P35573\|GDE_HUMAN | DGSAVEIVGLSK.light | FALSE | 587.82 | Unit | 844.51 | Unit | 10 | 130 | 19.2 | 4 | Positive |
| sp\|P35573\|GDE_HUMAN | DGSAVEIVGLSK.light | FALSE | 587.82 | Unit | 745.45 | Unit | 10 | 130 | 19.2 | 4 | Positive |
| sp\|P35573\|GDE_HUMAN | DGSAVEIVGLSK.light | FALSE | 587.82 | Unit | 404.25 | Unit | 10 | 130 | 19.2 | 4 | Positive |
| sp\|P35573\|GDE_HUMAN | DGSAVEIVGLSK.light | FALSE | 587.82 | Unit | 147.11 | Unit | 10 | 130 | 19.2 | 4 | Positive |
| sp\|Q14938\|NFIX_HUMAN | AFSYTWFNLQAR.light | FALSE | 752.37 | Unit | 1035.54 | Unit | 10 | 130 | 24.3 | 4 | Positive |
| sp\|Q14938\|NFIX_HUMAN | AFSYTWFNLQAR.light | FALSE | 752.37 | Unit | 934.49 | Unit | 10 | 130 | 24.3 | 4 | Positive |
| sp\|Q14938\|NFIX_HUMAN | AFSYTWFNLQAR.light | FALSE | 752.37 | Unit | 748.41 | Unit | 10 | 130 | 24.3 | 4 | Positive |
| sp\|Q14938\|NFIX_HUMAN | AFSYTWFNLQAR.light | FALSE | 752.37 | Unit | 175.12 | Unit | 10 | 130 | 24.3 | 4 | Positive |
| sp\|Q14938\|NFIX_HUMAN | GIPLESTDGERLYK.light | FALSE | 789.41 | Unit | 1197.57 | Unit | 10 | 130 | 25.5 | 4 | Positive |
| sp\|Q14938\|NFIX_HUMAN | GIPLESTDGERLYK.light | FALSE | 789.41 | Unit | 1068.53 | Unit | 10 | 130 | 25.5 | 4 | Positive |
| sp\|Q14938\|NFIX_HUMAN | GIPLESTDGERLYK.light | FALSE | 789.41 | Unit | 765.43 | Unit | 10 | 130 | 25.5 | 4 | Positive |
| sp\|Q14938\|NFIX_HUMAN | GIPLESTDGERLYK.light | FALSE | 789.41 | Unit | 147.11 | Unit | 10 | 130 | 25.5 | 4 | Positive |
| sp\|Q9H2U1\|DHX36_HUMAN | DPFVIPLGK.light | FALSE | 493.29 | Unit | 527.36 | Unit | 10 | 130 | 16.3 | 4 | Positive |
| sp\|Q9H2U1\|DHX36_HUMAN | DPFVIPLGK.light | FALSE | 493.29 | Unit | 414.27 | Unit | 10 | 130 | 16.3 | 4 | Positive |
| sp\|Q9H2U1\|DHX36_HUMAN | DPFVIPLGK.light | FALSE | 493.29 | Unit | 204.13 | Unit | 10 | 130 | 16.3 | 4 | Positive |
| sp\|Q9H2U1\|DHX36_HUMAN | DPFVIPLGK.light | FALSE | 493.29 | Unit | 147.11 | Unit | 10 | 130 | 16.3 | 4 | Positive |
| sp\|Q9H2U1\|DHX36_HUMAN | TDGLVAVHPK.light | FALSE | 518.79 | Unit | 820.50 | Unit | 10 | 130 | 17.1 | 4 | Positive |
| sp\|Q9H2U1\|DHX36_HUMAN | TDGLVAVHPK.light | FALSE | 518.79 | Unit | 650.40 | Unit | 10 | 130 | 17.1 | 4 | Positive |
| sp\|Q9H2U1\|DHX36_HUMAN | TDGLVAVHPK.light | FALSE | 518.79 | Unit | 551.33 | Unit | 10 | 130 | 17.1 | 4 | Positive |
| sp\|Q9H2U1\|DHX36_HUMAN | TDGLVAVHPK.light | FALSE | 518.79 | Unit | 244.17 | Unit | 10 | 130 | 17.1 | 4 | Positive |
| sp\|O15355\|PPM1G_HUMAN | QLIVANAGDSR.light | FALSE | 572.31 | Unit | 902.47 | Unit | 10 | 130 | 18.7 | 4 | Positive |
| sp\|O15355\|PPM1G_HUMAN | QLIVANAGDSR.light | FALSE | 572.31 | Unit | 789.38 | Unit | 10 | 130 | 18.7 | 4 | Positive |
| sp\|O15355\|PPM1G_HUMAN | QLIVANAGDSR.light | FALSE | 572.31 | Unit | 690.32 | Unit | 10 | 130 | 18.7 | 4 | Positive |
| sp\|O15355\|PPM1G_HUMAN | QLIVANAGDSR.light | FALSE | 572.31 | Unit | 262.15 | Unit | 10 | 130 | 18.7 | 4 | Positive |
| sp\|O15355\|PPM1G_HUMAN | NTAELQPESGK.light | FALSE | 587.29 | Unit | 958.48 | Unit | 10 | 130 | 19.2 | 4 | Positive |
| sp\|O15355\|PPM1G_HUMAN | NTAELQPESGK.light | FALSE | 587.29 | Unit | 887.45 | Unit | 10 | 130 | 19.2 | 4 | Positive |
| sp\|O15355\|PPM1G_HUMAN | NTAELQPESGK.light | FALSE | 587.29 | Unit | 758.40 | Unit | 10 | 130 | 19.2 | 4 | Positive |
| sp\|O15355\|PPM1G_HUMAN | NTAELQPESGK.light | FALSE | 587.29 | Unit | 517.26 | Unit | 10 | 130 | 19.2 | 4 | Positive |
| sp\|P30837\|AL1B1_HUMAN | VAFTGSTEVGHLIQK.light | FALSE | 793.93 | Unit | 1168.63 | Unit | 10 | 130 | 25.6 | 4 | Positive |
| sp\|P30837\|AL1B1_HUMAN | VAFTGSTEVGHLIQK.light | FALSE | 793.93 | Unit | 695.42 | Unit | 10 | 130 | 25.6 | 4 | Positive |
| sp\|P30837\|AL1B1_HUMAN | VAFTGSTEVGHLIQK.light | FALSE | 793.93 | Unit | 275.17 | Unit | 10 | 130 | 25.6 | 4 | Positive |
| sp\|P30837\|AL1B1_HUMAN | VAFTGSTEVGHLIQK.light | FALSE | 793.93 | Unit | 147.11 | Unit | 10 | 130 | 25.6 | 4 | Positive |
| sp\|P30837\|AL1B1_HUMAN | EEIFGPVQPLFK.light | FALSE | 702.38 | Unit | 1032.59 | Unit | 10 | 130 | 22.8 | 4 | Positive |
| sp\|P30837\|AL1B1_HUMAN | EEIFGPVQPLFK.light | FALSE | 702.38 | Unit | 885.52 | Unit | 10 | 130 | 22.8 | 4 | Positive |
| sp\|P30837\|AL1B1_HUMAN | EEIFGPVQPLFK.light | FALSE | 702.38 | Unit | 828.50 | Unit | 10 | 130 | 22.8 | 4 | Positive |
| sp\|P30837\|AL1B1_HUMAN | EEIFGPVQPLFK.light | FALSE | 702.38 | Unit | 504.32 | Unit | 10 | 130 | 22.8 | 4 | Positive |
| sp\|Q05639\|EF1A2_HUMAN | YYITIIDAPGHR.light | FALSE | 709.87 | Unit | 979.53 | Unit | 10 | 130 | 23 | 4 | Positive |
| sp\|Q05639\|EF1A2_HUMAN | YYITIIDAPGHR.light | FALSE | 709.87 | Unit | 765.40 | Unit | 10 | 130 | 23 | 4 | Positive |
| sp\|Q05639\|EF1A2_HUMAN | YYITIIDAPGHR.light | FALSE | 709.87 | Unit | 652.32 | Unit | 10 | 130 | 23 | 4 | Positive |
| sp\|Q05639\|EF1A2_HUMAN | YYITIIDAPGHR.light | FALSE | 709.87 | Unit | 537.29 | Unit | 10 | 130 | 23 | 4 | Positive |
| sp\|Q05639\|EF1A2_HUMAN | EVSAYIK.light | FALSE | 405.22 | Unit | 581.33 | Unit | 10 | 130 | 13.6 | 4 | Positive |
| sp\|Q05639\|EF1A2_HUMAN | EVSAYIK.light | FALSE | 405.22 | Unit | 423.26 | Unit | 10 | 130 | 13.6 | 4 | Positive |
| sp\|Q05639\|EF1A2_HUMAN | EVSAYIK.light | FALSE | 405.22 | Unit | 260.20 | Unit | 10 | 130 | 13.6 | 4 | Positive |
| sp\|Q05639\|EF1A2_HUMAN | EVSAYIK.light | FALSE | 405.22 | Unit | 147.11 | Unit | 10 | 130 | 13.6 | 4 | Positive |
